# Geochemical Decoupling of Iron and Zinc during Transformation of Zn-Bearing Ferrihydrite in Reducing Sediments

**DOI:** 10.1021/acs.est.4c09261

**Published:** 2024-11-04

**Authors:** Pierre Lefebvre, Andrew R. C. Grigg, Ruben Kretzschmar

**Affiliations:** Soil Chemistry Group, Institute of Biogeochemistry and Pollutant Dynamics, Department of Environmental Systems Science, ETH Zürich, Universitätstrasse 16, CHN, CH-8092 Zürich, Switzerland

**Keywords:** mineral transformation, Mössbauer spectroscopy, X-ray absorption spectroscopy, environmental speciation, green rust, zinc sulfide, zinc carbonate

## Abstract

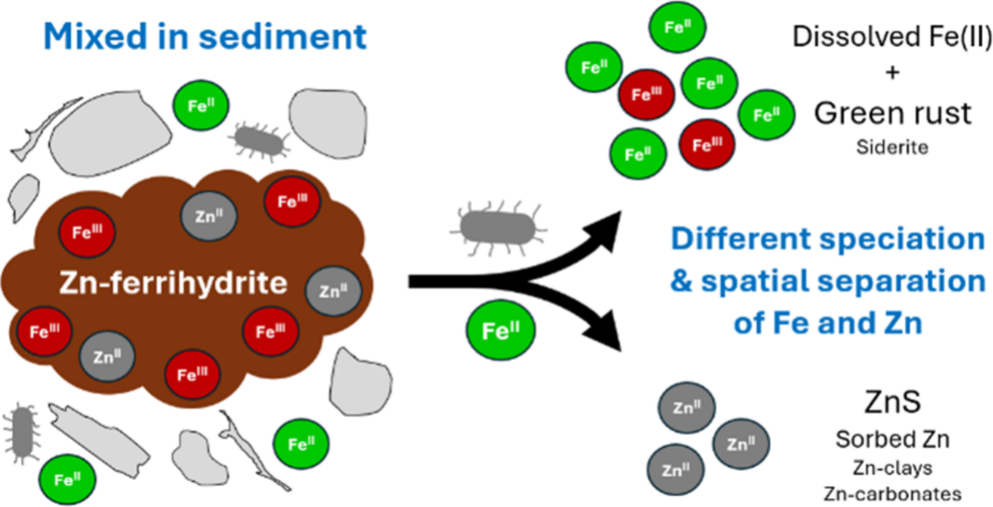

The transformation of the mineral ferrihydrite in reducing environments, and its impact on the mobility of incorporated trace metals, has been investigated in model laboratory studies, but studies using complex soil or sediment matrices are lacking. Here, we studied the transformation of zinc (Zn)-bearing ferrihydrite labeled with ^57^Fe and mixed with natural sediments, incubated in reducing conditions for up to six months. We tracked the evolution of Fe and Zn speciation with ^57^Fe Mössbauer spectroscopy and with bulk and micro-X-ray absorption spectroscopy. We show that Fe was readily reduced and incorporated into a poorly crystalline mixed-valence Fe(II)–Fe(III) phase resembling green rust. In parallel, Zn was released in the surrounding porewater and scavenged by precipitation with available ligands, particularly as zinc sulfide (ZnS) or Zn-carbonates. Early in the mineral transformation process, the chemical behavior of Fe was decoupled from Zn, suppressing the impact of Zn on the rates and products of the ferrihydrite transformation. Our results underline the discrepancy between model experiments and complex field-like conditions and highlight the importance of sediment and soil geochemistry and ligand competition on the fate of divalent metal contaminants in the environment.

## Abstract 2

We investigated the in situ mineral transformation of Zn-bearing ferrihydrite in natural sediments, showing that Zn and Fe behave differently and incorporate into different mineral products, in contrast with model laboratory studies.

## Introduction

In natural soils and sediments, iron (Fe) minerals often control the mobility of nutrients and contaminants, including trace metals.^1−3^ Ferrihydrite (herein abbreviated Fh) is a poorly crystalline Fe oxyhydroxide commonly found in the environment which has a very large specific surface area, providing a large sorption capacity. In natural systems, Fh usually contains many trace element impurities incorporated into its structure and/or adsorbed onto its surface.^4−7^ However, Fh is also metastable in reducing (sub)surface environmental conditions where dissimilatory microbial Fe(III) reduction occurs, and can transform into more crystalline Fe minerals, a reaction that may be catalyzed by dissolved Fe(II) through dissolution-reprecipitation processes.^8−12^ The presence of impurities can affect the Fh transformation rate, mainly by hindering the nucleation and/or growth or more crystalline phases. This has been demonstrated for sorbed organic matter,^11,13,14^ but also sorbed or coprecipitated (oxy)anions and cations such as trace metals.^13,15−20^ Among these, zinc (Zn) is of particular interest as it can be both an essential nutrient for plants and bacteria, and a contaminant if present in high concentrations in soils.^21−25^ The geochemical behavior of Zn may also be comparable in some ways to several other divalent transition metals such as cobalt (Co) and nickel (Ni) or even copper (Cu) and cadmium (Cd).^26−29^

The control exerted by ferrihydrite on the mobility and availability of Zn has been explored in model laboratory systems where mixed mineral suspensions of ferrihydrite with adsorbed or coprecipitated Zn were exposed to dissolved Fe(II). These experiments showed that Fh transforms into a variety of mineral products depending on the conditions (pH, Fe(II) concentrations, temperature, biotic/abiotic conditions), which were dominated by crystalline Fe (oxyhydr)oxides such as goethite, hematite or magnetite.^18,29−33^ Additionally, a few studies have investigated the transformation of Zn-adsorbed Fe (oxyhydr)oxides in sediment slurries.^29,31^ The transformation of Zn-bearing Fh can release Zn into the surrounding aqueous phase, which is often followed by reincorporation into the crystalline Fe mineral transformation products depending on their structure and affinity toward Zn.^18,29−33^ However, studies using complex, static sediment or soil matrices are lacking to better constrain the fate of Zn-bearing ferrihydrite in natural environments.

Here, we add a layer of complexity to controlled Fe(II)-catalyzed Fh transformation studies by investigating the transformation of Zn-bearing ferrihydrite (further referred to as Fh–Zn) within flooded intertidal sediments in laboratory mesocosms mimicking field conditions. We used a novel approach developed by our team which consists of incubating ^57^Fe-labeled minerals diluted in a complex matrix and applying ^57^Fe–Mössbauer spectroscopy which specifically targets the labeled mineral and its transformation products.^34−38^ Our objectives were to assess the impact of Zn on the rates and mineral products of Fh–Zn transformation within natural sediments, as compared to model laboratory experiments, and to evaluate the fate of Zn during the mineral transformation. We hypothesized that the presence of Zn would slow down the Fh transformation,^18,32^ in addition to other factors that slow Fh transformation in static mesocosms, such as diffusion limitations and multiple possible interferences from the complex sediment matrix, including organic matter.^34−37,39^ We also expected possible impacts on the products of transformation, because of the influence of dissolved components of the porewater such as organic matter, metals or anions, and of the solid soil matrix.^34−37^ During the transformation, Zn could either be reincorporated into the newly formed Fe mineral products,^29−33^ scavenged in other minerals through precipitation or adsorption^27^ or stabilized in the porewater. To investigate these hypotheses, we combined ^57^Fe Mössbauer spectroscopy with bulk and micro-X-ray absorption spectroscopy (XAS) at the Zn and Fe K-edges to analyze Fh–Zn and its transformation products within reducing sediments over several months of incubation.

## Materials and Methods

### Mesocosm Setup

We prepared two laboratory mesocosms using natural intertidal flat sediments collected from two sites from the Wadden Sea (Northern Germany): in Friedrichskoog (noted as FKS), at the mouth of the Elbe River estuary,^38^ and in Hollerwettern (noted as HW), on the shores of the estuary. Both sediments are similar in elemental composition (Table S1), the main differences being slightly higher Fe and Zn contents in HW (11 g/kg and 76 mg/kg respectively) compared to FKS (8.2 g/kg and 45 mg/kg) and a higher salinity in FKS (higher Na and Cl contents) due to its location closer to the sea. The texture of HW sediment is more clayey while FKS is more sandy. We used a mesocosm setup very similar to the one described in Schulz et al.^35^ (Figure S1). Briefly, 20 L plastic boxes were filled with sediment up to 12 cm, and initially flooded with ultrapure water (Milli-Q, >18.2 MΩ·cm) for 1 week. The boxes were drained for 4 weeks and then flooded again. Although reflooded with oxygenated water, the mesocosms remained in reducing conditions (Figure S7d). We consequently chose to apply permanent flooding to the sediments for the rest of the experiment, using artificial seawater that was diluted to match the concentrations measured at the field sites (Table S2). There was no need to replenish flooding water throughout the experiment. The two mesocosm boxes were monitored throughout the experiment for major physicochemical parameters (pH, Eh, matric potential, temperature). Further details on the mesocosms setup are provided in the Supporting Information.

### Sample Preparation

Three batches of ^57^Fe-labeled ferrihydrite containing 0, 0.5, and 5 wt % of coprecipitated Zn (further referred to as Fh–Zn 0, 0.5, and 5%) were synthesized from ^57^Fe(0) powder (96.14% ^57^Fe, Isoflex) and ZnCl_2_ (Merck) using a method adapted from Schwertmann and Cornell.^40^ The identity and purity of synthesized 2-line Fh–Zn was verified by X-ray diffraction (XRD, Bruker D8 Advance) (Figure S2). The Zn contents, which are equivalent to Zn/(Fe + Zn) molar ratios of 0, 0.78 and 7.8% respectively, were confirmed by acid digestion and measurement with an Agilent 5100 inductively coupled plasma optical emission spectrometer (ICP-OES). Previous analyses of Zn-bearing ferrihydrite have shown a homogeneous Zn distribution.^41^ The synthesis and characterization procedures are further detailed in the Supporting Information.

Mixes of 10 mg Fh–Zn and 200 mg sediment (further referred to as Fh-sediment mixes) were then prepared for each mesocosm with duplicates for each of the four time steps and three Zn contents. One additional mix with Fh–Zn 5% was prepared for each mesocosm, for the evaluation of Zn and ^57^Fe diffusion out of the mesh bag. In total, 50 Fh-sediment mixes were prepared. The mineral:sediment ratio was higher than in comparable previous studies^34−37^ to ensure sufficient Zn content for Zn K-edge X-ray absorption spectroscopy (XAS) while keeping the Fe addition as low as possible. This resulted in >99% ^57^Fe coming from the added Fh–Zn, with 3 to 4 times increase of the initial sediment Fe content, and 4 to 60 times increase of the Zn content (Table S3). Although such additions are substantial, they may be compared to local hotspots in the sediment. The Fh-sediment mixes were transferred into porous mesh bags (approximate internal dimensions ∼10 × 30 × 2 mm^3^) custom-made with two layers of PETE mesh fabric (pore size of 51 μm that allows exchange with the external sediment), carefully sealed with a heat-sealer. The mesh bags were then placed in three-dimensional (3D)-printed sample holders (Clear Photopolymer Resin, Formlabs) with large windows, allowing contact with the mesocosm sediment and equipped with a rod that facilitates sample insertion and retrieval in/from the sediment (Figure S1).^37^ All samples were inserted at a depth of 8 cm in the sediment at the same time (*t* = 0), 2 days after sediment reflooding.

### Sampling

Porewater was collected in triplicate at sample depth before sample insertion and before each mesh bag sampling, using three permanently installed Rhizon samplers (Rhizosphere Research Products, The Netherlands) per mesocosm. Separate aliquots were collected for the analyses of major, minor and trace cations, dissolved organic carbon (DOC), Fe speciation, sulfides and alkalinity.

After 2, 4, 9, and 16 weeks of incubation, the mesh bags (in duplicate) were collected from each mesocosm with care to prevent their exposure to air. These incubation times are suitable for tracking the progressive transformation of ferrihydrite mixed with sediment.^34−37^ The samples were opened and stored in an anaerobic glovebox (N_2_ atmosphere, MBraun). Each transformed Fh-sediment mix was named using the mesocosm abbreviation (HW or FKS), the Zn content (0, 0.5 or 5), the number of incubation weeks (2, 4, 9 or 16) and a letter for the replicate (A or B): for example, HW 0 16A refers to Fh–Zn 0% reacted for 16 weeks in the HW mesocosm (replicate A).

After 18 weeks of incubation, aliquots of reduced sediment were sampled in both mesocosms at 3–4 cm depth. At 26 weeks, larger sediment cores were collected and subsampled to evaluate the diffusion of Zn and ^57^Fe from a mesh bag containing Fh–Zn 5% (Figure S3). All sediment samples were dried, ground, homogenized and stored inside the glovebox. The mesh bag inside each core (reacted for 26 weeks) was recovered and treated the same way as other mesh bags.

We performed chemical extractions on mesh bag samples as well as sediment samples with 0.5 M HCl at room temperature for 1 h in the glovebox^42^ (Figure S4), to evaluate the fraction of poorly crystalline Fe phases and associated Zn. Although the exact identity of the phases targeted by such extractions is poorly constrained, it is expected that such a concentration of HCl will mainly dissolve poorly ordered Fe (oxyhydr)oxide phases such as ferrihydrite,^43^ allowing at least a cross-comparison between samples. We determined the Fe oxidation state and ^57^Fe isotope fraction (*f*^57^Fe, relative to the sum of isotopes ^54^Fe, ^56^Fe, ^57^Fe and ^58^Fe) of HCl-dissolved Fe. Mass balance equations were used to quantify the fraction of HCl-extracted Fe coming from transformed Fh–Zn (with ^57^Fe-enriched composition) compared to Fe coming from the sediment. Additional information on sampling and HCl extraction procedures are shown in the Supporting Information.

### ^57^Fe Mössbauer Spectroscopy

The Fe mineralogy of initial and transformed Fh-sediment mixes and some sediment samples was analyzed by ^57^Fe Mössbauer spectroscopy in transmission mode with a ^57^Co source and a constant acceleration drive system over a −12 to 12 mm/s velocity range in a standard setup (WissEl, Wissenschaftliche Elektronik GmbH) equipped with a closed-cycle He cryostat (SHI-850, Janis Research Co.). All samples were analyzed at 77 and 5 K, with additional temperatures (140, 60, 50, 40, 30, 20, and 10 K) for a selection of samples. The experimental spectra were calibrated with an α-Fe(0) foil regularly measured at room temperature, then folded and fitted with the Recoil software.^44^

### X-ray Absorption Spectroscopy

Bulk X-ray absorption near-edge structure (XANES) and extended X-ray absorption fine structure (EXAFS) analysis was performed on selected samples at the Zn and Fe K-edges at the BM23 beamline of the European Synchrotron Radiation Facility (ESRF, Grenoble, France). All measurements were performed at 10 K, either in transmission (with ion chambers) or fluorescence mode (with a Vortex Si-drift fluorescence detector). Reference metal foils (Zn or Fe) were used for calibration in double-transmission setup.

Selected samples were chemically probed by synchrotron micro-X-ray fluorescence (XRF) imaging, complemented by micro-XANES measurements on selected points, at the BM23 beamline. The samples were exposed to air, at room temperature. First, micro-XRF maps were acquired with a beam energy of 12 keV (5 μm steps). The XRF spectra were calibrated and converted to element-specific fluorescence intensity maps with the PyMca software.^45^ Based on visual evaluation of the chemical maps, selected points were targeted for micro-XANES analyses at the Zn and/or Fe K-edges. Successive measurements on several spots did not show any significant beam-induced damage.^46^ All XAS spectra were examined, merged, calibrated and normalized using the Athena software.^47^ The XANES and EXAFS spectra were then analyzed by linear combination-least-squares fitting (further abbreviated as LCF) on Athena, using a large set of reference spectra from our databases covering all expected Zn and Fe species (Figures S5 and S6). More details are provided in the Supporting Information.

## Results and Discussion

### Sediment and Porewater Conditions

**Figure 1:**
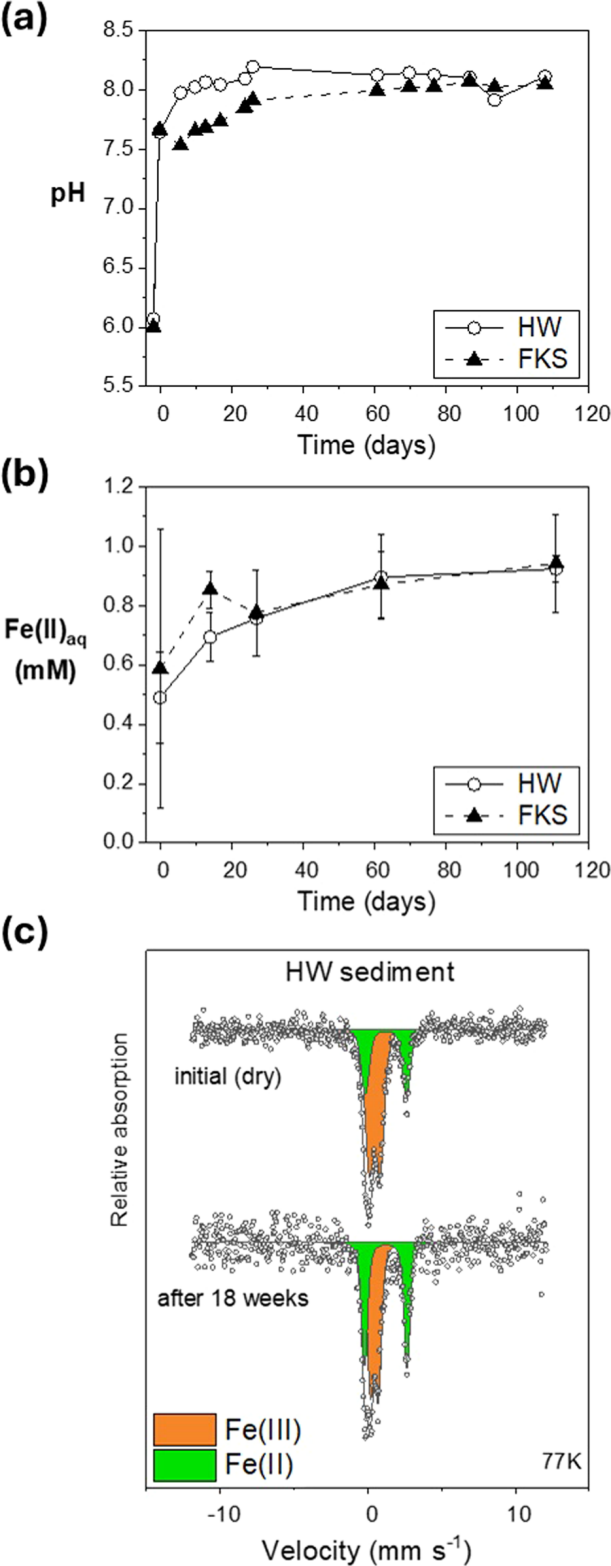
Evolution over the incubation of (a) pH in the mesocosm overlying waters and (b) the aqueous concentrations of Fe(II) in porewaters. Measurements from the HW mesocosm are shown with open circles and solid lines, from FKS with plain triangles and dashed lines. The error bars show 2SD uncertainties from triplicate porewater samples. (c) Mössbauer spectra at 77 K of initial dry HW sediment (top) and HW sediment incubated for 18 weeks (bottom). The experimental data is shown as open circles, the calculated fit as a black line, and the fitted components as filled areas: orange for the Fe(III) doublet and green for the Fe(II) doublet.

The flooding had an immediate effect on the geochemical conditions in the incubation mesocosms. The pH of overlying water increased to >7.6 within 2 days and stabilized within a few weeks at pH 8.0–8.1 in both HW and FKS mesocosms (Figures 1a and S7), similar to seawater and consistent with field measurements.^38^ The redox potential (Eh) consistently decreased from the first flooding on and was relatively stable from the time of sample insertion to the end of the experiment; in FKS, the last sampling at week 26 occurred at the onset of reoxidation (Figure S7). Strongly reducing conditions were established in both mesocosms (Eh from −300 to −250 mV in HW, −290 to −230 mV in FKS), at values that are favorable to microbially mediated Fe(III) reduction and even sulfate reduction.^48,49^

The establishment of Fe-reducing conditions was visible in Mössbauer and Fe K-edge XAS measurements of the sediments before flooding (dry) and after 18 weeks of incubation. Mössbauer spectra acquired at 77 K show that the contribution of Fe(II) increased by 15–20% of total Fe (Figures 1c and S8 and Table S4). Fe XANES and EXAFS show a similar increase of the Fe(II) proportions by 20–30% of total Fe upon flooding, and indicate that Fe was mainly present in clay minerals (fitted as illite and smectite), along with small amounts in Fe (oxyhydr-)oxides and traces of pyrite (4–10%) (Figures S9–S11 and Table S5). The observation of 20–30% Fe oxyhydroxides fitted as ferrihydrite in dry sediments justifies the choice of Fh–Zn as starting material for our experiments. Comparable proportions of total Fe were chemically extracted by 0.5 M HCl before and after flooding (33–35% from HW sediment, 23–24% from FKS), but the Fe(II)–Fe(III) proportions of extracted Fe were reversed after incubation, with 85 to 95% Fe(II) in HW and FKS respectively (Figure S12 and Table S6). This shows that “available Fe” (estimated here as HCl-extractable Fe) was easily reduced, likely by microbial activity, even though a large fraction of Fe remained as Fe(III) likely in the structure of minerals and kinetically protected from microbial reduction. Dissolved Fe in the porewaters was consistently dominated by Fe(II) (76–96%), with concentrations increasing from 0.5–0.6 to 0.9 mM in both mesocosms, corresponding to 1.1–1.2% of solid-phase available Fe(II) after 16 weeks (Figures 1b and S13). Such Fe(II) concentrations are known to be favorable to ferrihydrite mineral transformation, e.g., ref (8).

The aqueous concentrations of most elements in porewaters tended to stabilize after 2–4 weeks of incubation (Figure S13). Apart from DOC concentrations that are approximately 70 mg/L in both HW and FKS mesocosms, we observed clear differences in porewater compositions that are attributed either to salinity contrasts (much higher Na and Mg in FKS) and/or to sediment composition (higher Ca, Mn and S in HW, higher P in FKS). HW and FKS porewaters showed comparable levels of alkalinity (approximated by dissolved (bi)carbonate) at 12.4 ± 1.2 mM and 9.7 ± 1.9 mM respectively. In both mesocosms, dissolved sulfides were undetectable (<10 μM). Aqueous concentrations of trace metals were often below ICP-OES detection limit, including dissolved Zn (<0.1 mg/L).

### Characterization of Initial Fh-Sediment Mixes

Mössbauer spectra acquired at 140 and 77 K of the initial mixes of dry sediment with Fh–Zn 0, 0.5 and 5% showed unique paramagnetic Fe(III) doublets that are consistent with ferrihydrite (Figure S14 and Table S7), as expected since >99% of ^57^Fe comes from added Fh–Zn (Table S3). Using an extended Voigt-based fitting (xVBF) model,^50^ all ferrihydrite doublets were fit with a similar center shift (CS) at 0.46 mm/s and with a quadrupole shift (QS) widening between 140 and 77 K, from 0.92–0.93 to 1.25–1.28 mm/s for Fh–Zn 0 and 0.5% and from 0.87 to 1.02 mm/s for Fh–Zn 5% (Table S7), consistent with the effect of decreasing temperature.^34^ Temperature profiles (Figure S15) show that the Fh doublets start transforming into sextets between 60 and 50 K for Fh–Zn 0 and 0.5% (fully ordered around 30 K), and between 50 and 40 K for Fh–Zn 5% (fully ordered around 20 K).

An initial Fh-sediment mix with HW sediment and Fh–Zn 5% (HW5 ini) was probed by micro-XRF mapping (Figures S16 and S17), showing a homogeneous dispersion of Fh–Zn within the soil matrix, ranging from <5 to 30 μm sizes (within the 0.8 × 0.8 mm^2^ probed area). Micro-XANES spectra at the Fe and Zn K-edges confirmed the identity of Fh–Zn 5% particles as well as the presence of Fe-rich clays and pyrite, but no Zn-rich particles that did not contain Fe (Figures S18 and S19 and Tables S8 and S9). HCl extractions of the initial Fh-sediment mixes resulted in dissolution of most Fh–Zn (78–88%), with no resolvable influence of the Zn content or sediment type (Figure S20).

### Iron Mineral Transformation of Fh–Zn

After incubation, chemical extractions of the transformed Fh-sediment mixes showed that only 17 to 42% of Fe initially present as Fh–Zn (i.e., Fe in mineral products of Fh–Zn transformation) was dissolved by 0.5 M HCl (Figure S20), meaning that a substantial proportion of Fe was either lost from the mesh bag to the surrounding sediment or incorporated into HCl-resistant crystalline minerals. The Mössbauer spectra of solid residues after HCl extractions (Figure S21) were noisy and contained features that were consistent with phases in the original sediment (Figures 1c and S8). In addition, excess ^57^Fe was measured in sediments collected around a mesh bag containing Fh–Zn 5% in concentrations consistent with the amount of “missing” ^57^Fe (Figure S12 and associated discussion). Altogether, these observations indicate that most Fe in Fh–Zn transformation products was dissolved by 0.5 M HCl, and therefore that 58 to 83% of Fe originating from Fh–Zn was lost from the mesh bag to surrounding sediment within the first 2 weeks, likely by reductive dissolution followed by diffusion. A similar process was also reported in other studies using similar mesh bags.^35−38^ Fe losses were slightly higher in the FKS mesocosm (66–83% lost Fe) than in HW (58–74%) (Figure S20), which may be linked to the coarser texture of FKS sediment, favoring diffusion of dissolved Fe in the porewater.^35^

**Figure 2:**
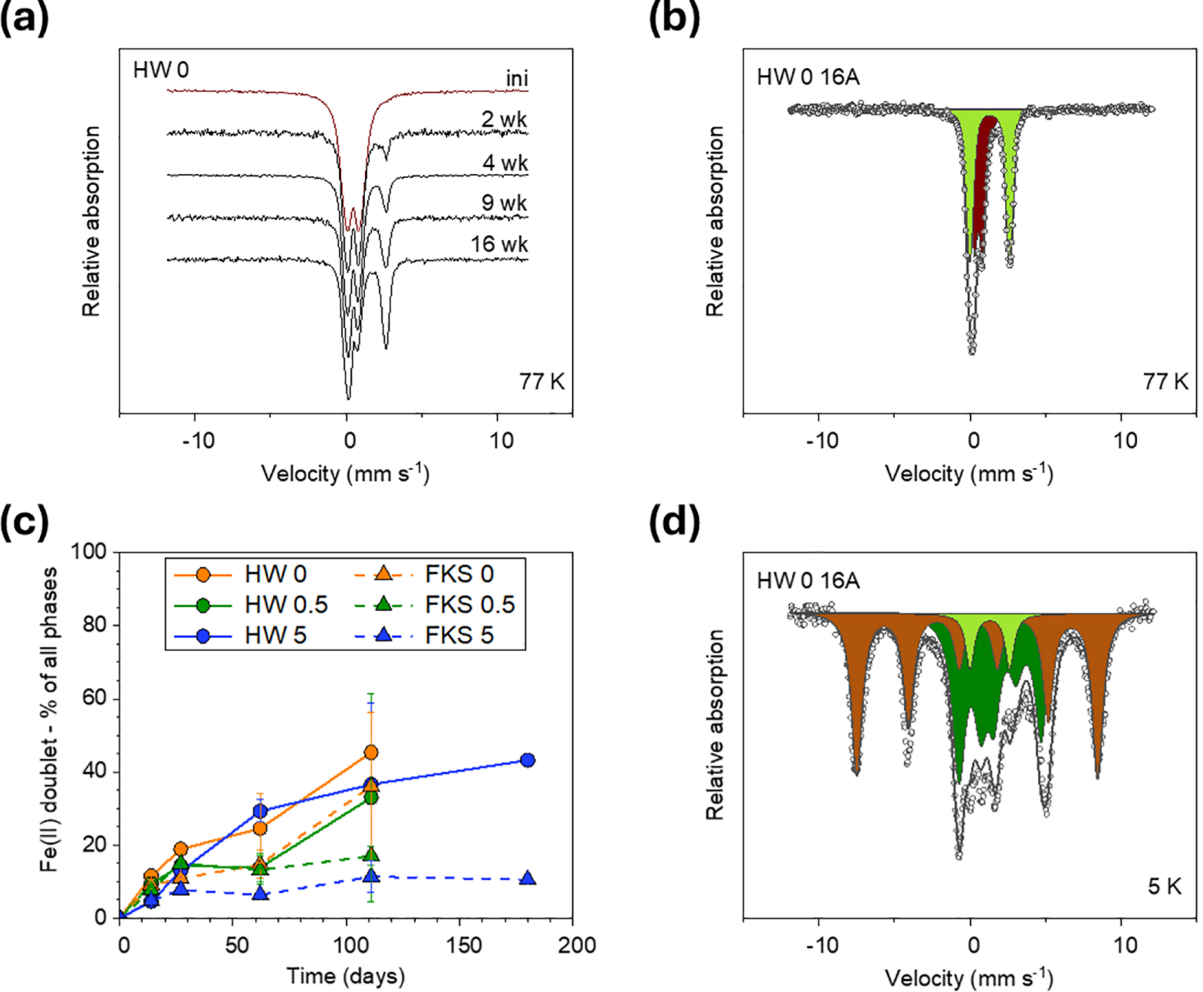
(a) Mössbauer spectra acquired at 77 K of transformed Fh–Zn:sediment mixes containing Fh–Zn 0% incubated in the HW mesocosm (HW 0, replicates A) for 0 (ini), 2, 4, 9, and 16 weeks; the spectra were normalized to maximum absorption for visual comparison. (b) Mössbauer spectrum at 77 K and fit solution of sample HW 0 16A (16 weeks); the experimental data is shown as open circles, the calculated fit as a black line, and the two main components as filled areas: green for the Fe(II) doublet, dark red for the Fe(III) doublet. (c) Evolution over time of the Fe(II) proportions (Mössbauer Fe(II) doublet) of transformed Fh–Zn:sediment mixes in HW (circles, solid line) and FKS (triangles, dashed line) mesocosms and each of the Fh–Zn 0% (orange), 0.5% (green) and 5% (blue). (d) Mössbauer spectrum at 5 K and proposed fit solution of sample HW 0 16A; the experimental data is shown as open circles, the calculated fit as a black line, and the three main components as filled areas: light green for the Fe(II) doublet, dark green for the Fe(II) octet, brown for the Fe(III) sextet.

As expected, the mineral products of Fh–Zn transformation could not be detected by XRD because of their dilution in the sediment matrix (data not shown). Despite the ^57^Fe losses, Fe in transformed Fh–Zn still makes >96.5% of total ^57^Fe, so that the Mössbauer signal is still largely dominated by the solid products of Fh–Zn mineral transformation. The Mössbauer spectra at 77 K and associated fits and parameters of all analyzed transformed Fh-sediment mixes are presented in Figures S24–S30 and Tables S10–S12, with average parameters reported in Table S13. Apart from one outlier (Figure S27 and Table S10), all samples transformed similarly, as exemplified in Figure 2a with 77 K Mössbauer spectra of samples HW 0 (Fh–Zn 0% in HW) incubated for 0 to 16 weeks: in addition to a paramagnetic Fe(III) doublet (initially corresponding to Fh–Zn), an Fe(II) doublet already appears after 2 weeks of incubation and grows between sampling time points. The position of the Fe(II) doublet relative to the Fe(III) doublet as shown in Figure 2b was determined using spectra acquired at lower temperatures, especially at 30 K where the Fe(III) doublet has transformed into a sextet and enables confident fitting of the Fe(II) doublet (Figure S22). On average, the Fe(III) doublet has a CS of 0.48 mm/s and a QS of 0.71 mm/s, and the Fe(II) doublet is fit with a CS of 1.25 mm/s and a QS of 2.78 mm/s (Table S13). It should be noted that more complex solutions involving multiple doublets were possible (Figure S23) but yielded unstable fits with high uncertainties, so the simplest solution was retained, keeping in mind that the Fe(II) and Fe(III) components may be a mixture of several phases with similar Mössbauer features.

The Fe(II) component contribution to the Mössbauer signal over the 26-week incubation period is shown in Figure 2c. Overall, the Fe(II) proportions in the solid phase never exceed 50%, and the rate of transformation seems slower in FKS than in HW. However, if we consider all Fe(II) originating from transformed Fh–Zn, i.e., solid-phase ^57^Fe(II) analyzed by Mössbauer and dissolved Fe lost by diffusion (approximated as 100% Fe(II)), it appears that 60–76% of Fe from Fh–Zn was reduced after 2 weeks, and even 80–87% after 16 weeks, without any significant difference between Zn contents or between HW and FKS sediments (Figure S31), which is consistent with the similar porewater Fe(II) concentrations in both mesocosms (Figure 1). Although the low temporal resolution limits comparisons with Fh–Zn transformation rates measured in laboratory model experiments, we can state that the rate of Fe(III) reduction is not much slower here than in model experiments.^18,29−33^

There is also no clear impact of the Zn content on the evolution of the Mössbauer fit parameters (Table S13 and Figures S32 and S33). The Mössbauer parameters of transformed Fh-sediment samples provide some indications of the identity of the Fe(III) and Fe(II) phases, with additional uncertainties due to the possibility of a complex mixture (Figure S23). The Fe(III) parameters at 77 K are still consistent with poorly ordered ferrihydrite^51^ but may also correspond to other Fe(III) phases such as lepidocrocite^52^ or Fe(III) associated with silicates or organic matter.^53^ The absence of Fe(III) sextets or octets at 77 K rules out any goethite, hematite or magnetite.^54^ The Fe phases differ slightly between HW and FKS (Table S13 and Figure S33). The phase identification in the Fe(II) doublet component could only be carried out at lower temperatures.

We therefore acquired spectra at several temperatures between 77 and 5 K for a selection of samples. The blocking temperature of the Fe(III) phase (at which 50% of the mineral is magnetically ordered^55^) decreases significantly and ultimately reaches similar values for Fh–Zn 0 and 5% (30–20 K) (Figures S34–S36), indicating that the Fe(III) phase becomes less ordered over the mineral transformation. The blocking temperature of the Fe(II) phase is less discernible as the signal is often dominated by the Fe(III) sextet, but it seems that Fe(II) starts ordering around 10 K and may still be not completely ordered at 5 K. The Fe(II) doublet progressively gives way to an octet (Figure S22d). Such a low blocking temperature rules out significant proportions of crystalline siderite (FeCO_3_) or ferrous hydroxide, which have higher Néel ordering temperatures of ∼37 and ∼34 K, respectively,^54,56^ and is more consistent with either green rust (GR) or vivianite (Néel temperatures of ∼5.2 and ∼12 K respectively^57,58^). However, vivianite is less plausible since we did not observe its characteristic high QS (3.2–3.3 mm/s) at 77 K.^38,59^

To fit selected Mössbauer spectra measured at 5 K, we applied a full static Hamiltonian (FSH) fitting model.^60^ All analyzed 5 K spectra and their corresponding fits are shown in Figures S37 and S38 and Table S14, with the example of sample HW 0 16A displayed in Figure 2d. The Fe(II) octet could be fit with parameters corresponding to green rust,^34,58,61,62^ i.e., an average CS of 1.57 mm/s, an electric quadrupole interaction parameter (*e*^2^*qQ*/2, equivalent to QS) of −2.98 mm/s and a hyperfine field (*H*) of 12.0 T, although the resulting feature did not match the data perfectly. On average, the Fe(III) sextet could be fit with a CS of 0.49 mm/s, *e*^2^*qQ*/2 of −0.05 mm/s and a *H* of 48.8 T. These parameters exclude lepidocrocite^52^ and are consistent with ferrihydrite,^51,63^ but may also correspond to the Fe(III) component of nonstoichiometric green rust.^58^ Overall, given the absence of a clear phase identification, we postulate that our samples could be made of poorly crystalline intermediate phases where Fe progressively transforms from ferrihydrite to a "green rust-like" phase.^34^ At this stage, the counteranion composition (carbonate, chloride or sulfate) of this putative “green rust-like” phase is unknown. Note that minor poorly crystalline Fe(II) phases may be indistinguishable from the main green rust-like feature. Overall, the main process affecting Fh–Zn is reductive dissolution followed by reprecipitation rather than Fe(II)-catalyzed transformation to crystalline Fe(III) minerals.

No Fe sulfides were detected with Mössbauer spectroscopy, although minor amounts of pyrite could not be excluded as they would appear as a doublet similar to Fe(III) at 77 K, e.g., ref (64). The exception is for the outlier sample HW 0 16B which might contain iron sulfide of unknown stoichiometry (FeS_*x*_) visible as a sextet at 77 K with a low hyperfine field of 28 T^65,66^ (Figure S27 and Table S10).

A deeper look into the identity of Fh–Zn transformation products was provided by bulk and micro-XAS at the Fe K-edge on samples reacted for 16 weeks. The bulk XAS signals of transformed Fh-sediment samples are dominated by the contribution from Fe from sediments (∼55–60% of total Fe after ^57^Fe losses) which mainly consists of Fe-bearing clays (Figures S9–S11 and Table S5). Linear combination fitting of XANES and EXAFS spectra provided consistent results, with small disparities inherent to the difference between both techniques (Figures S39–S41 and Table S15). In addition to the sedimentary Fe components (clays and pyrite), we observe the presence of siderite and carbonated green rust (GR(CO_3_)) in sample HW 5 16A, siderite in HW 0.5 16A, and only Fe (oxyhydr-)oxides in FKS 5 16A. In an attempt to remove the contribution from sedimentary Fe, we subtracted the spectrum of reduced HW sediment (55% of total Fe) from the XANES and EXAFS signal of sample HW 5 16A. LCF of the difference spectra seems to confirm the presence of siderite and GR(CO_3_), along with a small contribution from Fe(III) in illite which may come from the sediment or from the Fe(III) component of transformed Fh–Zn (Figures S39–S41 and Table S15).

**Figure 3:**
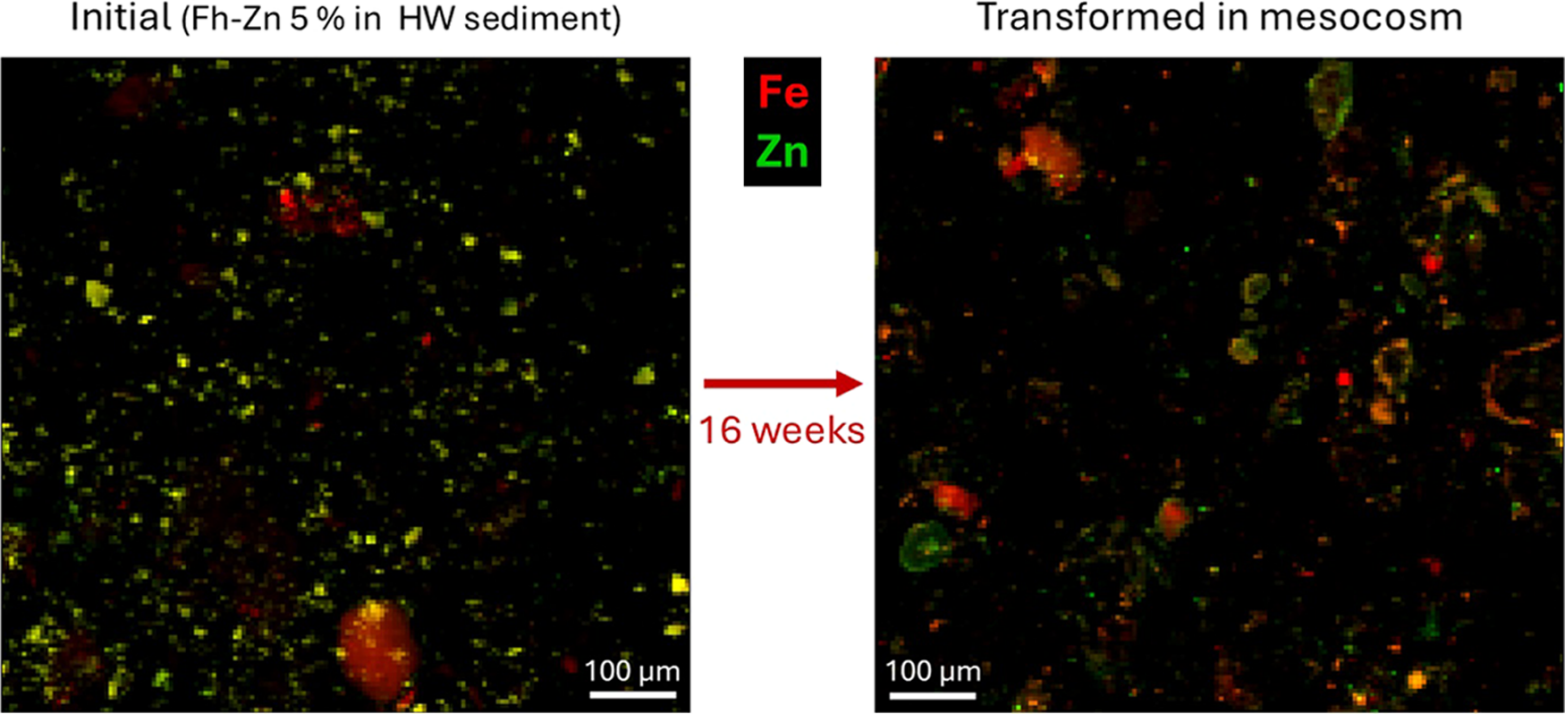
Micro-XRF maps of (left) the initial untransformed Fh–Zn 5% mixed in HW sediment (HW 5 ini) and of (right) the corresponding sample transformed over 16 weeks in the HW mesocosm (sample HW 5 16A). The intensities of Fe Kα and Zn Kα fluorescence lines are displayed in red and green respectively, yellow spots corresponding to co-occurrence of both elements (Fh–Zn 5% in the left panel). Fluorescence maps of individual elements can be found in Figures S16 and S42.

To obtain confirmation of these phases and assess the spatial distribution of Fe mineral phases, we performed micro-XRF followed by micro-XANES at the Fe K-edge on sample HW 5 16A. Micro-XRF chemical maps (Figures 3, S42, and S43) show that both elements are mainly partitioned into separate Fe- and Zn-rich regions, different from the initial state. Micro-XANES spectra at the Fe K-edge were collected on 11 selected points (Figures S43 and S44 and Table S16). Sparse grains with co-occurring Fe and Zn (appearing in yellow/orange on Figure 3 right, with lower Zn/Fe than initial Fh–Zn) correspond to slightly transformed, likely passivated grains of Fh–Zn which might have lost a fraction of their Zn content. Other Fe-rich grains included Fe-bearing clays or siderite which was clearly identified in a few grains with its unique double oscillation XANES feature (Figures S6 and S44d). We did not confirm the presence of GR(CO_3_) by micro-XANES, but this phase cannot be ruled out considering the potential selection bias of analyzed Fe hotspots. Additionally, exposure to air during the microbeam measurements may have oxidized oxygen-sensitive particles including green rust.

Overall, a large fraction of Fh–Zn particles totally transformed over the incubation in reducing conditions into a variety of mineral products which differ from the crystalline Fe(III) (oxyhydr)oxides observed in laboratory model experiments.^18,29−33^ This transformation was likely triggered by aqueous Fe(II) (∼0.9 mM). The lack of crystalline Fe mineral products may be due to the inhibiting role of dissolved components, including organic matter.^13,14^ The discrepancy between Mössbauer and XAS results suggests that at the sample scale, carbonated Fe(II) phases (GR(CO_3_), siderite) of variable crystallinity may coexist as intermediate states during the ongoing mineral transformation.^67,68^

### Fate of Zn during the Transformation

**Figure 4:**
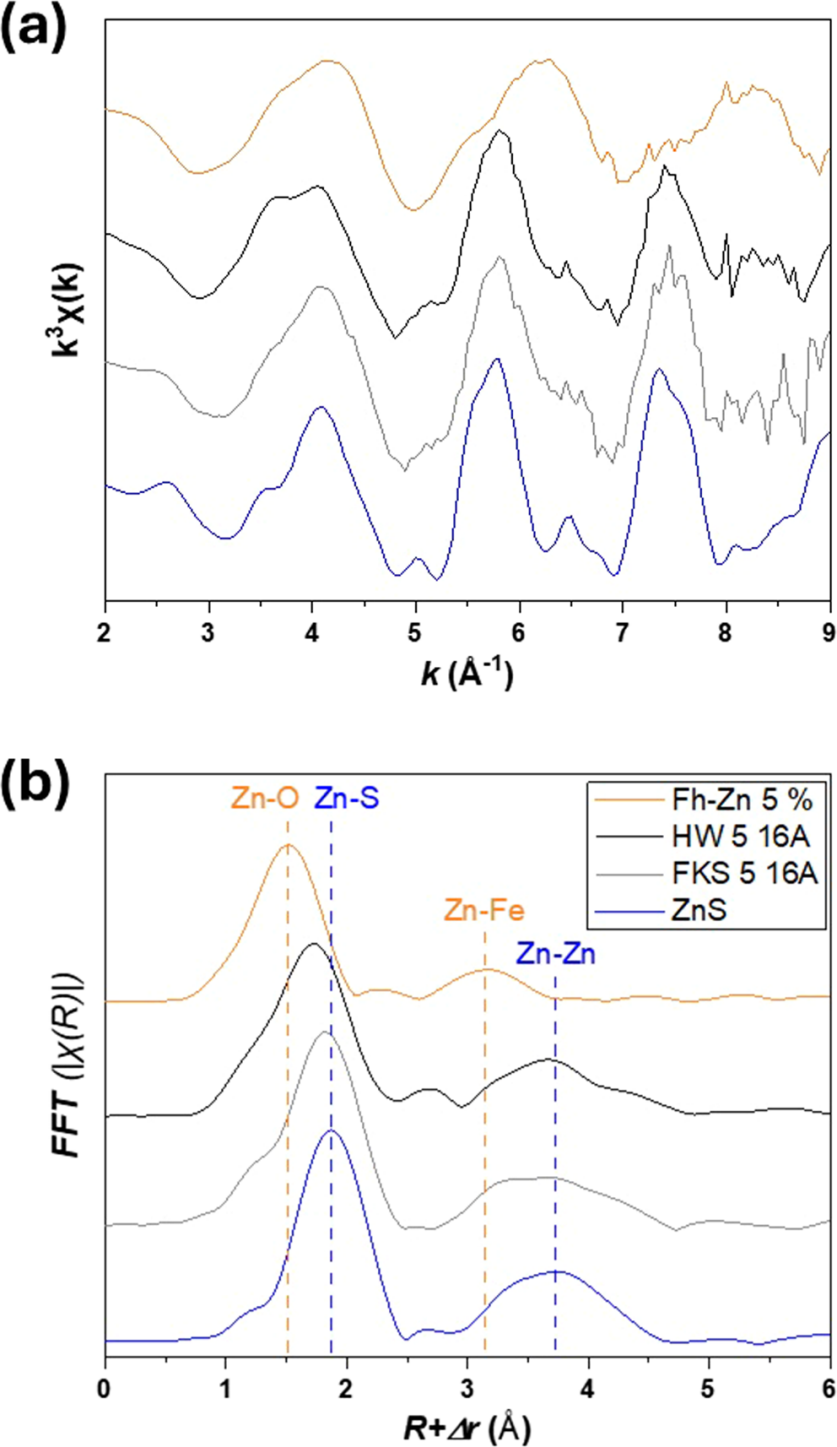
Zn K-edge EXAFS spectra (a) and corresponding Fast Fourier Transforms (b) of the initial Fh–Zn 5% (orange) and of ZnS (blue) compared to samples reacted for 16 weeks in HW (HW 5 16A, black) and FKS (FKS 5 16A, gray) mesocosms. In panel (b), the positions (uncorrected from phase shift) of the first neighboring shells around Zn are indicated with vertical dashed lines: Zn–O and Zn–Fe shells in Fh–Zn (orange) and Zn–S and Zn–Zn in ZnS (blue). The linear combination fits of samples (with additional reference compounds in the case of HW 5 16A) are shown in Figures S46 and S47.

Bulk XANES and EXAFS at the Zn K-edge were acquired on the same samples as for Fe except FKS sediments (Figures S45–S47 and Table S17). In initial dry HW sediment, the Zn speciation is shared mostly between Zn in tetrahedral coordination within Fe oxyhydroxides (with synthetic Fh–Zn 5% as reference compound) and Zn-bearing clays (modeled here by synthetic kerolite-type phyllosilicates); a small proportion of Zn is incorporated into carbonates. After 18 weeks of flooding of the HW sediment, the contribution of Fe oxides disappears and is replaced by sphalerite ZnS. Again, some differences are noted between XANES and EXAFS: in particular, ZnS is much more visible in EXAFS. In Fh–Zn samples transformed over 16 weeks, where most of the Zn signal comes from added Zn (Table S3), the bulk Zn speciation is dominated by ZnS and Zn in ferrihydrite, with minor contributions from Zn-bearing clays and Zn carbonates (Figures S45–S47 and Table S17). The Zn EXAFS signal of samples is clearly dominated by ZnS (Figure 4a and S46). In particular, this Zn coordination shift can be seen in the Fourier transforms of EXAFS (Figure 4b), with an increase in the first shell distance from tetrahedral Zn–O in Fh–Zn (∼1.50 Å uncorrected from phase shift) to Zn–S in sphalerite (∼1.85 Å), and also an increase in the distance of the main second shell, from Zn–Fe in Fh–Zn (∼3.2 Å uncorrected from phase shift) to Zn–Zn in ZnS (∼3.7 Å). The remaining Zn was fitted as Fh–Zn (19 to 42%, Table S17). Since most Fh–Zn was transformed as seen with Fe speciation, this phase should be interpreted as a global pool of sorbed Zn in tetrahedral coordination.

Sample HW 5 16A contains less ZnS than samples FKS 5 16A and HW 0.5 16A, which show a similar Zn speciation. It is particularly striking that the Zn speciation in FKS 5 16A is dominated by ZnS, the rest consisting mainly of tetrahedral sorbed Zn (or Fh–Zn), while this sample had limited Fe reduction, with only 11% of Fe(II) in the solid phase (Table S11). This finding highlights again the decoupling of Fe and Zn behaviors over the mineral transformation of Fh–Zn in reducing sediments. We attribute this contrasting behavior to dissolution–precipitation processes affecting Fh–Zn,^8−10,12^ where mineral-bound Fe would undergo atom exchange with dissolved Fe(II) that incorporates again in the mineral lattice, thus leading to slow Fe mineral transformation, while Zn is released in the porewater^69^ and subsequently scavenged by complexing agents with higher affinity toward Zn than Fe minerals, such as sulfides which precipitate in ZnS form.

We took a closer look into Zn behavior using micro-XRF (Figure 3) and micro-XANES at the Zn K-edge (Figure S48 and Table S18) on sample HW 5 16A. In addition to becoming decoupled from Fe, Zn seems to form coatings on a variety of grains, some of which are rich in Ca (with no Si or Al) and are likely made of calcium carbonate (Figures S42 and S43). A joint interpretation of Fe and Zn XANES where both were acquired on the same point is provided in Table S19. A few analyzed spots correspond to ZnS, including some small (≤5 μm) Zn hotspots (points 18–20) which might be ZnS spherules, e.g., ref (70). The Zn speciation in Fe- and Zn-rich particles where Fe speciation indicated poorly transformed Fh–Zn (points 23–26) is indeed consistent with Fh–Zn but also shows a contribution from franklinite ZnFe_2_O_4_, which could indicate a partial recrystallization of the remaining Zn. Some Zn clays were also detected. Nevertheless, most of the analyzed Zn-rich spots are dominated by Zn carbonates, which were modeled by three reference compounds: trace Zn (1500 ppm) in CaCO_3_, smithsonite (ZnCO_3_) and hydrozincite (Zn_5_(CO_3_)_2_(OH)_6_). The relative abundance of these different Zn carbonate species varies among the measured points and is interpreted as a continuum with variable contents of Zn and carbonate/hydroxyl groups. The majority of these Zn-carbonate particles were found on coatings around a variety of grains, including siderite grains and Ca-rich grains attributed to CaCO_3_. The over-representation of this Zn speciation compared to bulk Zn XAS measurements (16–18% as Zn carbonates, Table S17) is attributed to a sampling bias, as these coatings were more targeted during point selection for micro-XANES. Altogether, these results again underline how Fe and Zn behave separately, although exposed to the same local conditions.

### Decoupling of Fe and Zn Chemical Behaviors

Our favored interpretation of the decoupled behaviors of Fe and Zn lies in their contrasting affinities to ligands present in the local porewater environment. During Fh–Zn mineral transformation catalyzed by aqueous Fe(II), Zn and Fe are likely transiently released to porewater in the vicinity of the Fh–Zn particle.^29−31,33^ Then, each element reacts with the present complexing agents that show the highest affinity. In the case of Fe, the affinity is probably highest with the Fe mineral from which it was released, leading to reincorporation of released Fe atoms into the mineral or to catalysis of a transformation to another Fe mineral. On the other hand, Zn has a greater affinity to dissolved sulfides (e.g., ref (71)) and a similar affinity to dissolved (bi)carbonate as compared to Fe, causing its scavenging into ZnS or Zn carbonates depending on the local conditions. Reincorporation into the Fe mineral is possible in the absence of sulfide and (bi)carbonate. This scavenging likely explains the absence of detectable Zn in porewaters. Similarly, the high reactivity of dissolved sulfides probably prevented build-up in the porewaters up to detectable concentrations (<10 μM). On the other hand, the scavenging of most sulfides by Zn may explain the absence of Fe sulfide precipitation.

These mechanisms likely explain observations described above on the rates of Fe mineral transformation. Indeed, Zn decoupling from Fe is likely initiated in the first stages of Fh–Zn transformation, when Fe gets reduced in large proportions (Figure S31). Once separated from the transforming Fe mineral, Zn should not impact the transformation rate and products anymore. This explains the low influence of the initial Zn content (0, 0.5 or 5%) on the rates of Fe reduction, transformation and diffusive loss (Figures 2c and S20). The slight differences observed between HW and FKS mesocosms, although with similar aqueous Fe(II), are attributed to differences in the sediment and water compositions, e.g., higher salinity in FKS or possibly contrasting sulfide production rates.

Large amounts of Zn were chemically extracted by 0.5 M HCl, in proportions comparable to that of Fe (Figure S49 and associated discussion). This is consistent with the observed Zn speciation consisting of HCl-sensitive phases (Zn-ferrihydrite, Zn carbonates, ZnS), in contradiction with previous studies that show enhanced Zn incorporation into HCl-resistant minerals following transformation in pure mineral suspensions or freshwater sediment slurries.^29−31^ Therefore, even though Zn and Fe are decoupled, both elements still get incorporated into mineral phases that are easily extractable, including amorphous or poorly crystalline phases. At the millimeter to centimeter scale, the Zn mobility in the porewaters is highlighted by its diffusion outside of the sample mesh bag, as seen with Zn enrichment in sediments collected around transformed Fh–Zn 5%, particularly in FKS (Figure S12c).

### Environmental Implications

The investigation of Fh–Zn transformation within sediments by application of our original ^57^Fe-labeling technique highlighted major contrasts between model laboratory experiments using mixed mineral suspensions or sediment slurries^18,29−33^ and conditions more representative of a complex sediment matrix. In particular, we observed an unexpectedly low influence of Zn on the transformation rate and on the transformation products, which is due to decoupling of Fe and Zn chemical behaviors caused by competition between Fe minerals and sediment-originating ligands (sulfides, carbonates) that may not be present in controlled experiments. Another major difference is the identity of Fh–Zn transformation products, which is likely due to the dilution of Fe species within the sediment matrix, inhibiting the formation of crystalline Fe (oxyhydr)oxides observed in model laboratory experiments.^18,29−33^ The strong impact of the sediment matrix evidenced in the present work highlights the need to better constrain the applicability of laboratory studies to complex natural systems. However, there is still a gap in complexity between our mesocosm setups and natural environments, so that our results should also be considered with caution before applying them to field observations.

Contrary to our starting hypothesis based on published results,^18,29−33^ the present study suggests that Fe oxyhydroxides exert little control over the mobility of Zn in some Fe-reducing environments. The enhanced transformation of Zn-bearing Fe oxyhydroxides when exposed to aqueous Fe(II) combined with the contrasted behavior of Zn and Fe toward natural ligands in the sediment may lead to a quick decoupling of both elements’ chemical behavior, even when Fe and Zn are initially incorporated into the same mineral and exposed to the same local geochemical conditions. The stability of newly formed Zn species (ZnS, Zn-carbonates, Zn-clays) toward environmental disturbance (such as draining and subsequent oxidation) becomes the key factor controlling Zn mobility in such environments.^72,73^

The same global mechanism, i.e., the competition between available ligands, may govern the fate of other common divalent ions, such as Co, Ni, Cu, Cd or Pb.^26,29,71^ Depending on the availability of ligands with an affinity for the respective trace elements, the product phases of Fe mineral transformations may only partially control the fate of previously incorporated trace elements. The behavior of Zn in S-bearing sediments shows that sediment geochemistry may have a strong role in controlling the fate of mineral-bound trace elements.

## Supporting Information Available

Details on mesocosm setup, mineral synthesis, sampling procedures, chemical and mineralogical analyses. Additional data and discussion for porewaters, sediments, transformed Fh–Zn (chemical data, HCl extractions, Mössbauer spectroscopy, XAS) (PDF)
Supplementary Data: mesocosm monitoring (pH, Eh, MP, T), porewaters composition, HCl extractions (XLSX)
