# Supplementary material for: Geochemical Decoupling of Iron and Zinc during Transformation of Zn-Bearing Ferrihydrite in Reducing Sediments
Source: Environ Sci Technol. 2024 Nov 4;58(45):20224–34. doi: 10.1021/acs.est.4c09261 (PMC11562722; doi:10.1021/acs.est.4c09261)
Supplement: Supplementary file 1 — es4c09261_si_001.pdf [file es4c09261_si_001.pdf]

Supporting Information

for

Geochemical decoupling of iron and zinc during transformation of Zn-bearing ferrihydrite in reducing sediments

Pierre Lefebvre*, Andrew R. C. Grigg, Ruben Kretzschmar

Soil Chemistry Group, Institute of Biogeochemistry and Pollutant Dynamics, Department of Environmental Systems Science, ETH Zürich, Universitätstrasse 16, CHN, CH-8092 Zürich

Number of pages: 80

Number of figures: 49

Number of tables: 19

Environmental Science & Technology

*Corresponding author: Pierre Lefebvre, pierre.lefebvre@usys.ethz.ch

Table of contents

1. Mesocosms setup 3
2. Zn-ferrihydrite synthesis 6
3. Sampling procedures 8
4. Chemical analyses 12
5. Mineralogical analyses 13
6. Physico-chemical parameters of the mesocosms 19
7. Sediment composition 21
8. HCl extraction of sediments 26
9. Pore waters composition 28
10. Characterization of initial Fh-Zn:sediment mixes 30
11. HCl extraction of transformed Fh-Zn: Fe results 38
12. Mössbauer of transformed Fh-Zn:sediment mixes 41
13. Fe bulk XAS 62
14. Micro-XRF of transformed sample HW 5 16A 66
15. Fe K-edge micro-XANES of transformed sample HW 5 16A 68
16. Zn bulk XAS 70
17. Zn K-edge micro-XANES of transformed sample HW 5 16A 74
18. HCl extraction of transformed Fh-Zn: Zn results 78

References 80

1. Mesocosms setup

For the laboratory mesocosms, we used natural intertidal flat sediments collected at two sites from the Wadden Sea (Northern Germany). Large amounts of sediments were collected in August 2020 in Hollerwettern (noted as HW; 0-30 cm depth), on the shores of the Elbe River estuary, and in Friedrichskoog (noted as FKS; 0-15 cm depth), at the mouth of the estuary (Kubeneck et al., 2024). The wet sediments were stored at 4 °C until they were dried in May 2023 in an oven at 40 °C, gently ground, homogenized and sieved at 2 mm.

Aliquots of dry milled sediments from HW and FKS (initial and from Control and Zn cores 26w) were analyzed for their bulk chemical composition by X-Ray Fluorescence (XRF; XEPOS, Spectro). The values from 6-10 cm samples of Control cores 26w of HW and FKS (see Figure S3) reported in Table S1, obtained from triplicate pellets of each sediment, were used as background values (for Fe and Zn initial additions and for HCl-extracted proportions).

Table S1 – Elemental composition of FKS and HW sediments measured by XRF on "Control cores 26w" sampled in mesocosms at 6-10 cm depth. Average values and 2SD uncertainties shown in brackets were calculated from measurements on three pellets of each sediment.

Element	FKS	HW	Element	FKS	HW
**Na (g/kg)**	8(3)	1.1(8)	**Cu (mg/kg)**	<0.5	2.8(14)
**Mg (g/kg)**	7.9(7)	7.9(6)	**Zn (mg/kg)**	**45(3)**	**76(4)**
**Al (g/kg)**	42.4(3)	45.9(6)	**Ga (mg/kg)**	3.3(16)	3.8(9)
**Si (g/kg)**	341(27)	310(13)	**Ge (mg/kg)**	1.5(3)	2.0(4)
**P (g/kg)**	0.87(5)	1.12(1)	**As (mg/kg)**	4.5(5)	7.9(1.4)
**S (g/kg)**	1.95(9)	3.37(16)	**Se (mg/kg)**	1.1(7)	0.83(12)
**Cl (g/kg)**	7.1(4)	0.37(4)	**Br (mg/kg)**	9.5(9)	8.2(2)
**K (g/kg)**	15.8(2)	15.3(3)	**Rb (mg/kg)**	61(2)	65(3)
**Ca (g/kg)**	26.8(3)	33(2)	**Sr (mg/kg)**	129(2)	139(3)
**Ti (g/kg)**	2.85(1)	2.87(12)	**Y (mg/kg)**	24.4(6)	24.5(3)
**V (mg/kg)**	39(6)	45(8)	**Zr (mg/kg)**	706(26)	506(16)
**Cr (mg/kg)**	70(16)	70(15)	**Nb (mg/kg)**	10.6(10)	10.4(6)
**Mn (g/kg)**	0.26(2)	0.81(12)	**Mo (mg/kg)**	1.0(9)	0.6(6)
**Fe (g/kg)**	**8.22(7)**	**11.1(16)**	**Ba (mg/kg)**	315(17)	339(7)
**Co (mg/kg)**	40(10)	36(18)	**Pb (mg/kg)**	15.6(6)	17.2(14)
**Ni (mg/kg)**	3.5(10)	6.8(43)	**Th (mg/kg)**	6.2(16)	5.3(9)

We used a mesocosm setup very similar to the one described in Schulz et al. (2023). The mesocosms consisted of two nested 20 L polypropylene boxes (Figure S1). A series of 5-mm

diameter holes was drilled on the bottom of the upper box (containing the sediment), ensuring water connection with the lower box, which is equipped with a valve to allow drainage. A polyethylene terephthalate (PETE) mesh fabric (SEFAR, Switzerland) with a pore size of 105 µm was placed at the bottom of the upper box, covered with 1 cm of 0.1-0.5 mm-sieved quartz sand and covered again with mesh fabric. Approximately 11-12 kg of sediment were initially flooded with double-deionized water and slowly placed in the box to reach a thickness of 12 cm, with gentle mixing to remove large air bubbles. After one week of flooding, a first drainage cycle was initiated by opening the lower box valve. In both boxes, the sediments retained a significant amount of water, and remained in reducing conditions even after four weeks of drainage (Figure S7d). Re-flooding the sediment with oxygenated water (as could happen with rising tide) after four weeks did not cause any significant rise in Eh (Figure S7d). These observations suggest that even in the field, these sediments are likely not affected by significant drainage and consecutive oxygen penetration during low tides and are therefore not subject to substantial redox oscillations. To mimic field-like conditions, we consequently applied permanent flooding to the sediments, using artificial diluted seawater with concentrations close to those previously measured at the field site (Kubeneck et al., 2024), i.e. with a dilution factor of 70 for HW and 1.5 for FKS compared to average simplified seawater (Table S2). The flooding water was slowly and homogeneously poured over the sediments, until the water level reached 3 cm above the sediment, and the boxes were covered with a plastic film to limit evaporation. The mesocosms were placed for the entire experiment in a climate chamber where temperature was set at 22 °C, relative humidity at 50 % and in darkness.

In each box, a gel-filled redox glass electrode (Mettler Toledo; 3 M KCl, Ag/AgCl reference electrode) coupled with a mobile logger system (pH Meter 913, Metrohm, built-in data logger) was inserted at sample depth (8 cm), recording the oxidation-reduction potential (ORP) every 30 minutes. The ORP values were then converted to redox potentials (Eh) relative to the standard hydrogen electrode (Eh-ORP = 210 mV). The matric potential and the surface temperature were also monitored every 30 minutes with a tensiometer (METER group) combined with a logger (ZL6, METER Group), inserted at 8 cm. The pH was regularly monitored in the overlying water with a pH electrode (Metrohm) calibrated against commercial standard solutions.

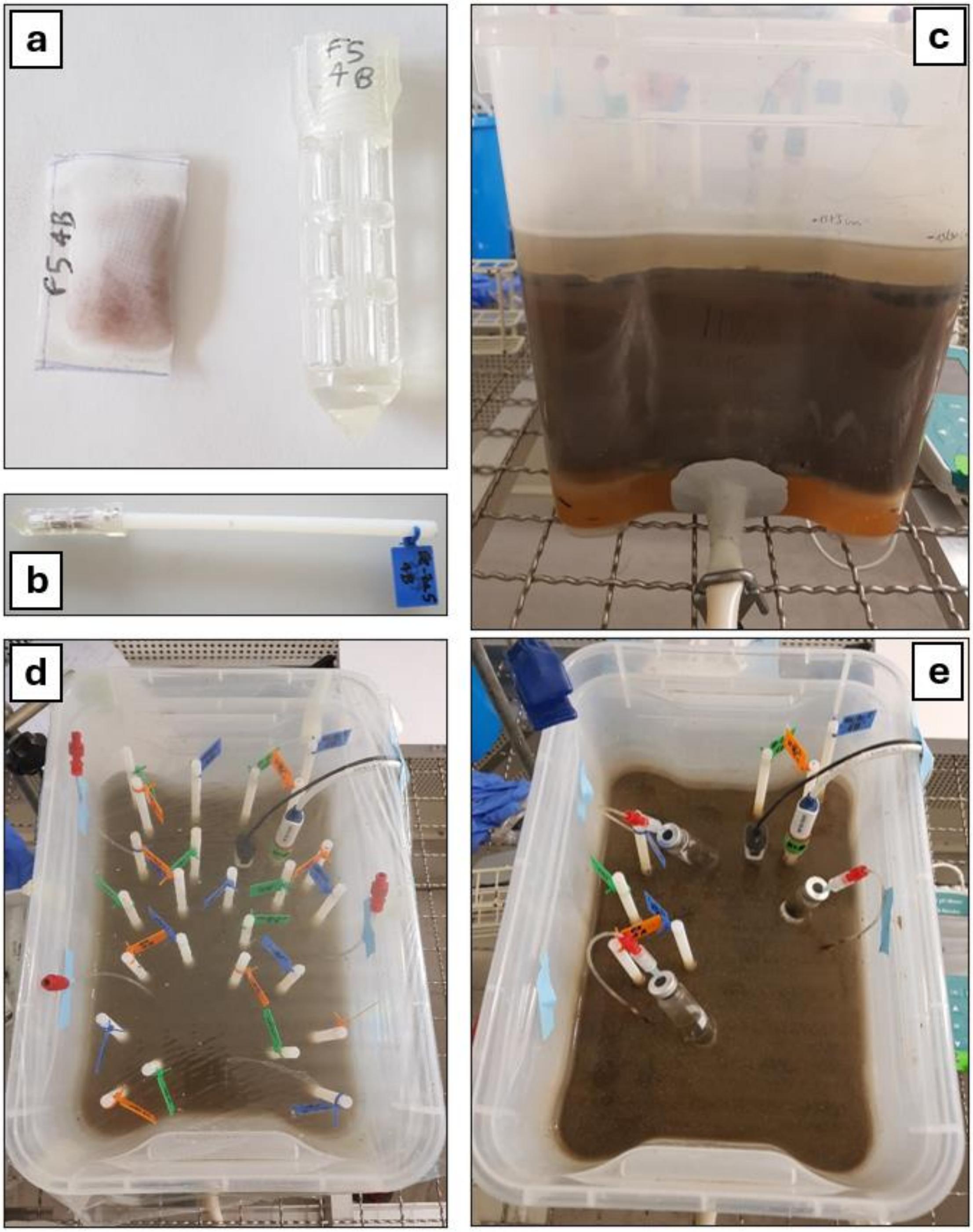

Figure S1 – Pictures of the experimental setup. (a) Sample mesh bag containing the dry Fh-Zn:sediment mix and its sample holder. (b) Sample holder filled with the mesh bag and equipped with a rod before insertion in the mesocosm. (c) Lateral view of a mesocosm box (after 16 weeks of incubation). (d) View from above a mesocosm box after sample insertion, showing the initial distribution of the samples (white rods with colored labels), the three pore water Rhizon samplers (tubes with red stoppers), the redox electrode (with white cable) and the tensiometer (with black cable). (e) Example of pore water sampling setup (at 16 weeks) showing the pore water vials and the former sample locations.

Table S2 – Chemical composition of the flooding water for the HW and FKS mesocosms. The major ion concentrations were calculated based on an average seawater (SW) composition, applying dilution factors of 70 for HW and 1.5 for FKS.

Mesocosm	HW (Hollerwettern) Dilution to SW: 70	FKS (Friedrichskoog) Dilution to SW: 1.5
**Concentration (mM)**		
**Na^+**	7.0	326
**Mg^{2+}**	0.76	35
**K^+**	0.15	6.8
**Ca^{2+}**	0.14	6.7
**Cl^-**	8.1	379
**SO_4^{2-}**	0.41	19

2. Zn-ferrihydrite synthesis

Three batches of ^{57}Fe-ferrihydrite containing 0, 0.5 and 5 wt% of coprecipitated Zn were made using a method adapted from Schwertmann and Cornell, (2000). Briefly, $^{57}Fe(0)$ powder (96.14 % ^{57}Fe, Isoflex USA) was dissolved overnight in 2 N HCl (Normatom, VWR); the solution (containing 0.16 M ^{57}Fe) was oxidized to $^{57}Fe(III)$ through addition of 35 % H_2O_2 (Merck) and passed through a 0.45 µm nylon filter. Three batches for each Zn content were made. Appropriate amounts of $ZnCl_2$ (Merck) were added and the solutions were mixed for homogenization for > 1 h and flushed with N_2 to limit CO_2 incorporation. The solutions were then neutralized with dropwise addition of a 1 M KOH solution (Titrisol, Merck) under vigorous stirring, until reaching a pH of ~7.4. The suspensions were stirred for > 1 h with regular pH adjustment (using 1 M HCl or KOH), then centrifuged at 3000 rpm for 15 minutes, rinsed with double-deionized water and centrifuged two times, shock-frozen by dropwise pouring into liquid N_2, and eventually freeze-dried. The dry material was ground, homogenized and stored in a desiccator until further use.

The identity of synthesized Fh-Zn 0, 0.5 and 5 % was verified by powder X-Ray Diffraction (XRD, Bruker D8 Advance) in Bragg-Brentano geometry using Cu K_α radiation. A small aliquot of ground sample was deposited on a Si(711) wafer with an ethanol droplet. The wafer was placed on a rotating stage (30 rotations per second) and analyzed from 10 to 70 ° (2θ) with 0.02 ° steps and a 6 s counting time per point. The mineralogy of the Fh-Zn samples was confirmed by XRD to be 2-line ferrihydrite, as shown in Figure S2, with two broad diffraction peaks centered around 35 and 62 ° (2θ) and no crystalline mineral impurities.

The Zn content of synthesized Fh-Zn was quantified by dissolving aliquots in 6 N HCl (Normatom, VWR) and analyzing the solution on an Agilent 5100 Inductively Coupled Plasma Optical Emission Spectrometry (ICP-OES). The Zn contents of Fh-Zn 0, 0.5 and 5 % were precisely measured at 0.00, 0.52 and 5.2 wt% (of total ferrihydrite) respectively, corresponding to Zn/(Fe+Zn) molar ratios of 0, 0.78 and 7.8 % respectively.

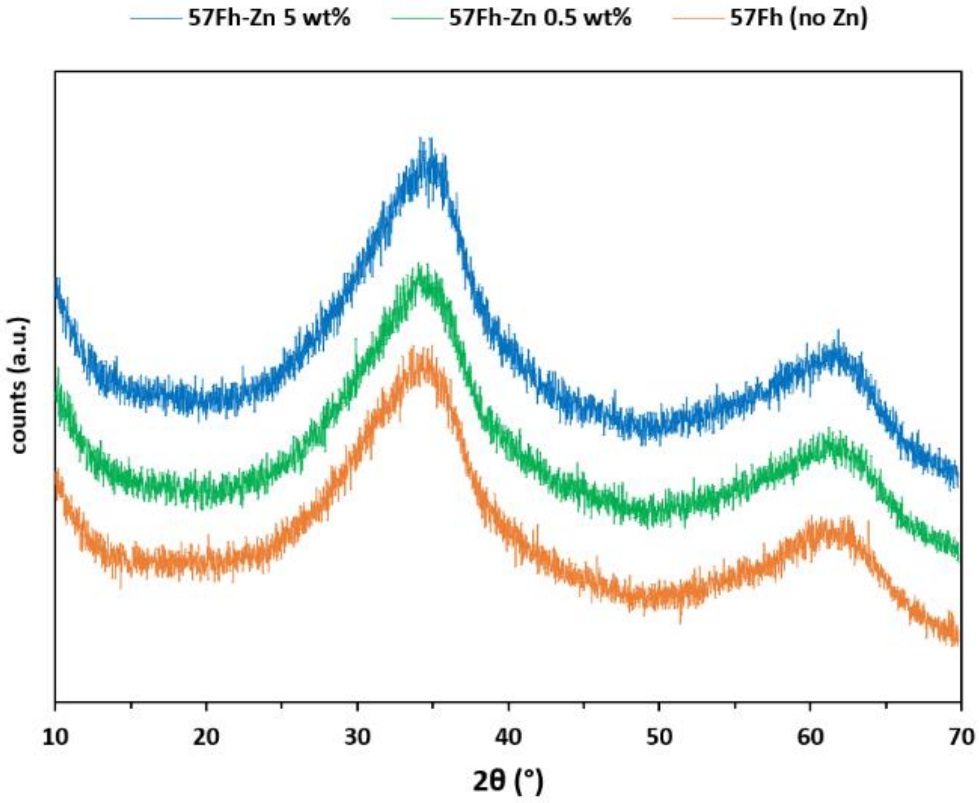

Figure S2 – X-ray diffraction patterns of synthesized ferrihydrites with different Zn contents: Fh-Zn 0 % in orange, Fh-Zn 0.5 % in green, Fh-Zn 5 % in blue. The patterns were vertically shifted to facilitate comparison.

Table S3 – Percentages of Zn, Fe and ^{57}Fe coming from Fh-Zn in sediment:Fh-Zn mixes, and relative increases in Zn and Fe contents in mixes compared to sediment only.

		% from Fh-Zn in Fh-Zn:sediment mix			Increase compared to sediment only	
**Sediment**	**Fh-Zn %**	**Zn**	**Fe**	**^{57}Fe**	**Zn**	**Fe**
**HW**	**0**	0	71.8	99.2	+0 %	+255 %
	**0.5**	77.3	71.8	99.1	+340 %	+254 %
	**5**	97.1	70.1	99.1	+3388 %	+234 %
**FKS**	**0**	0	77.5	99.4	+0 %	+343 %
	**0.5**	85.1	77.4	99.4	+570 %	+343 %
	**5**	98.3	75.9	99.3	+5683 %	+315 %

3. Sampling procedures

Pore water sampling

Pore water samples were collected in triplicate at sample depth before sample insertion and before each mesh bag sampling, using three permanently installed Rhizon samplers (Rhizosphere Research Products, The Netherlands, 2 cm porous part, 0.6 µm pore cut-off) per mesocosm. For each sample, four glass vials were prepared for different analyses: a first vial containing 30 µL of 6 N HCl (Normatom, VWR) for the analysis of major, minor and trace cations by ICP-OES and dissolved organic carbon (DOC) analysis; a second vial with 60 µL of 6 N HCl for the measurement of Fe speciation by UV-Visible spectrophotometry; a third vial with 400 µL of a 20 % Zn-acetate (Sigma Aldrich) solution for the analysis of dissolved sulfides (trapped through precipitation as ZnS); a fourth empty vial for the analysis of anions by ion chromatography (IC). All vials were flushed with N_2 after closing with a silicon or a rubber septum to prevent air penetration. The pore water samples were collected by suction directly into the vials, by connecting the rhizon sampler to a needle inserted through the septum and by using air-tight syringes to create a vacuum in the vials. The first 1-2 mL of pore water was discarded, then approximately 2 mL were collected in each of the four vials. The IC vials were immediately placed into a freezer at -20 °C, while all other vials were stored in the fridge (4 °C) until analysis. Additional pore water samples were collected after 19 weeks in empty N_2-flushed vials for immediate alkalinity measurements.

Mesh bag sampling

After 2, 4, 9 and 16 weeks of incubation (steps 1, 2, 3 and 4 respectively), the mesh bags (in duplicate) were collected from each mesocosm. The mud-covered holders were immediately placed into air-tight bags (Malaga series, Fosa) that were vacuumed to limit exposure to air during the 10-15 minutes transfer to an anaerobic glovebox under N_2 atmosphere (MBraun). Once in the glovebox, the mesh bags were taken out of their holder and allowed to dry. The mesh bags were then carefully cut open and their contents recovered. A small amount of intact material was placed in a 2 mL Eppendorf tube, and the rest was gently ground with an agate mortar and pestle for homogenization, placed in another tube and stored in the glovebox until analysis.

Sediment sampling

After 18 weeks of incubation, sediments were sampled by taking small (~5 cm deep) sediment cores using thin plastic tubes (7 mm diameter) that were placed in vacuumed bags and transferred into the glovebox within 10 minutes. The core was cut open and sediment at a depth of 3-4 cm (labelled as "Sed 18w") was collected, dried, ground and homogenized. These sediment samples from HW and FKS were analyzed by Mössbauer spectroscopy and X-ray absorption spectroscopy.

After 26 weeks, larger sediment cores were collected in both mesocosms to evaluate the diffusion of Zn and ^{57}Fe from a mesh bag containing Fh-Zn 5 % (Figure S3). First, a control core (labelled as "Control core 26w") was sampled by inserting a Humax core sleeve (35 mm diameter) down to the sediment bottom with a length of ~11.5 cm, at a location distant from any previous mesh bag insertion. Then, a larger core labelled as "Zn core 26w" was collected by first inserting a 35-mm sleeve around the Fh-Zn 5 % sample, and an additional 51 mm sleeve around this first tube. All core sleeves were taken out of the sediment, closed with plastic lids, and shock-frozen in liquid N_2 to maintain their structure. The frozen cores were then taken inside the glovebox and sliced during thawing. The outer layer (1-2 mm) of sediment was first removed with a spatula. The control cores were cut into four slices: 0-2, 2-6, 6-10 and 10-11.5 cm. The Zn cores were sampled at similar depth intervals, starting from the outer concentric layer (between 51- and 35-mm tubes, labelled OUT), then the inner concentric layer inside the 35-mm tube. At sample depth (6.5-9.5 cm, centered around 8 cm), we collected the outer 8-mm layer of this inner core (labelled MID), then the inner sediment right around the sample holder (IN), and finally the sediment in contact with the mesh bags inside the windows of the sample holder (RW). The sampling of sediment cores is further

illustrated in Figure S3. The sediment slices were dried, ground, homogenized and stored inside the glovebox. The mesh bag inside each core was recovered and treated the same way as other mesh bags. The water content and sediment dry bulk density (DBD) were measured by weighing known volumes of sediment before and after oven drying at 40 °C overnight. We measured a DBD of 1.27 ± 0.05 g/cm^3 for HW and 1.40 ± 0.04 g/cm^3 for FKS.

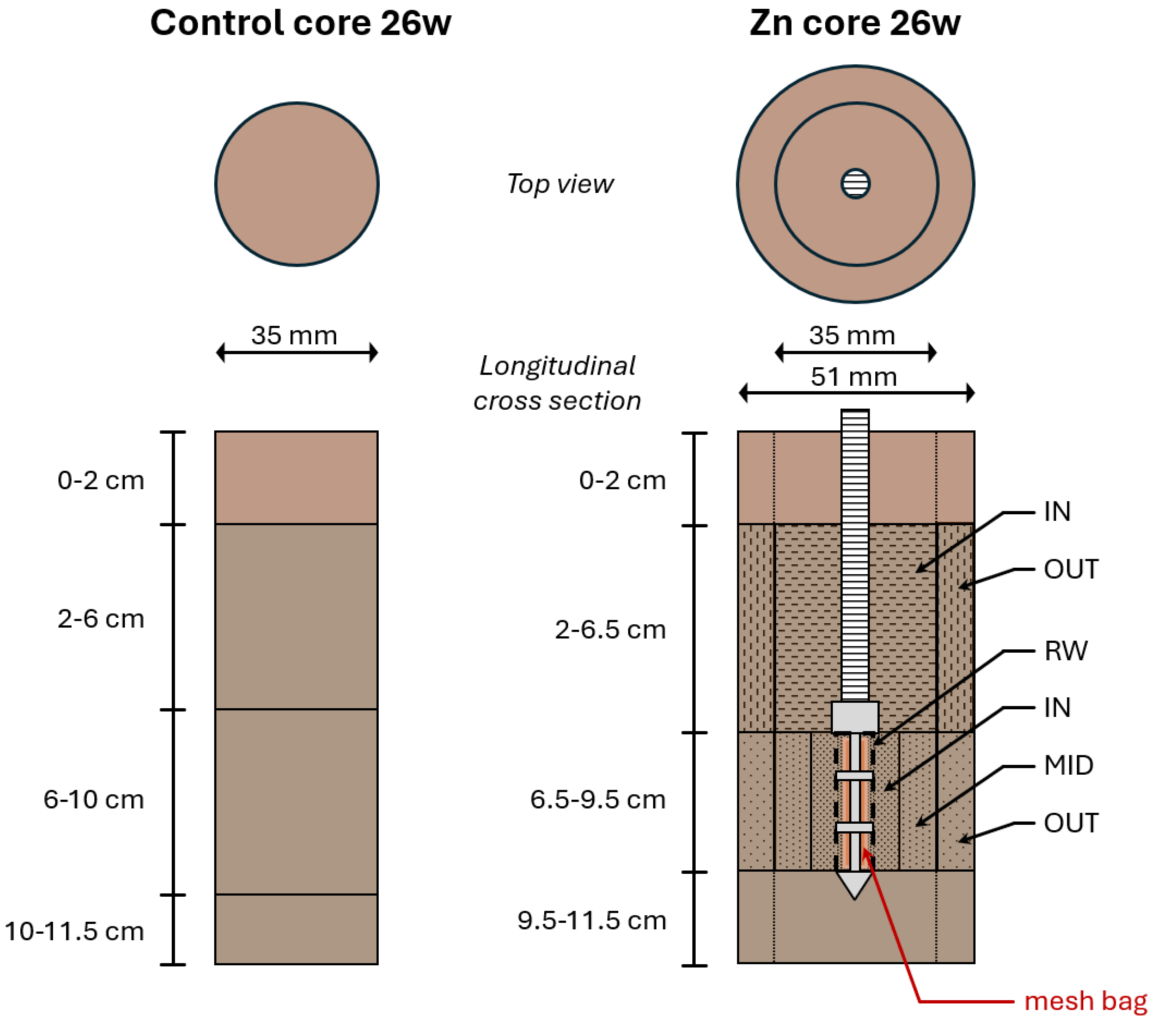

Figure S3 – Schematic description of the subsampling of sediment cores "Control core 26w" and "Zn core 26w", identical for both mesocosms. Control cores were sliced in four subsamples: 0-2, 2-6, 6-10 and 10-11.5 cm. Zn cores were divided into 8 subsamples: one at 0-2 cm and at 9.5-11.5 cm, two at 2-6.5 cm (OUT: between 51 and 35 mm tubes, IN: inside 35 mm tube), four at sample depth 6.5-9.5 cm (OUT: between 51 and 35 mm tubes; MID: 8 mm layer inside 35 mm tube, IN: inner sediment around the sample holder, RW: windows/openings of the sample holder, in contact with the mesh bag). The different color at 0-2 cm denotes the visual observation of more yellowish sediment in the upper 2 cm, which was used to delimitate this top layer from the underlying layer.

HCl extractions

All extractions were performed inside the anaerobic glovebox. A weighed amount of approximately 50 mg of material was suspended in 2 mL of HCl 0.5 M (Normatom, VWR) and shaken for one hour at room temperature. The suspension was then centrifuged at 6000 rpm for 5 minutes, and the supernatant was sampled and stored in the fridge (outside of the glovebox) until analysis. Preliminary kinetic tests showed that the amount of extracted total Fe and Fe(II) was stable between 40 min and 3 h of extraction time (Figure S4). Several duplicate extractions were performed and showed excellent reproducibility, with an average 5 % difference in extracted total Fe and Fe(II) proportions.

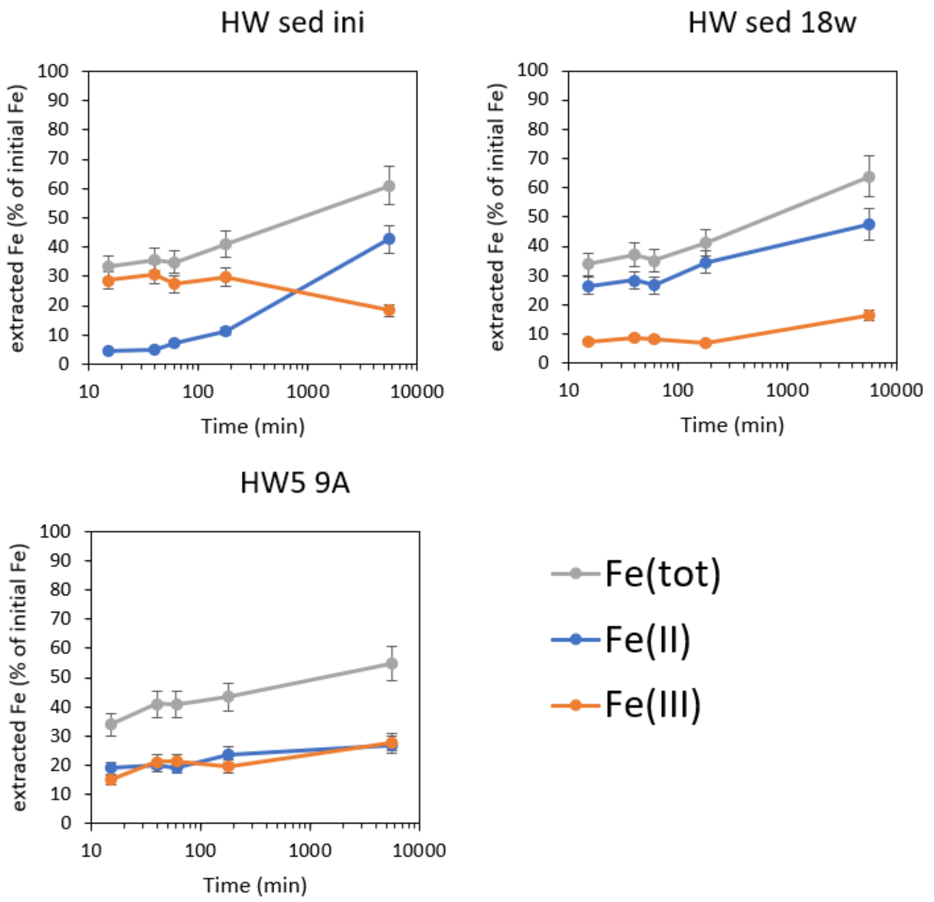

Figure S4 – Kinetic tests of 0.5 M HCl extractions of selected samples: initial (dry) HW sediment (top left), HW sediment core sampled after 18w (top right), Sediment:Fh-Zn mix (HW sediment, Fh-Zn 5 %) reacted for 9 weeks. Proportions of extracted Fe species are expressed as a percentage of initial total solid-state Fe in the sample: total Fe in gray, Fe(II) in blue, Fe(III) in orange. Aliquots (100 µL) were sampled after 15 min, 40 min, 1 h, 3 h and 4 days and analyzed for Fe speciation with the 1,10-phenanthroline method.

4. Chemical analyses

Pore water composition

Within one to two days after sampling, aqueous concentrations of Fe(II) and total Fe in pore waters were analyzed by UV-Visible spectrometry using the 1,10-phenanthroline method (e.g., Tamura et al., 1974) and used to calculate the percentage of Fe(II) in the aqueous phase. Within a few weeks after sampling, the total dissolved concentrations of major, minor and trace cations were measured by ICP-OES. Dissolved organic carbon (DOC) and nitrogen concentrations were measured on a DOC analyzer (DIMATOC 2000, Dimatec).

The dissolved sulfides (HS^-, S^{2-}, H_2S) were analyzed in all pore water aliquots (where sulfides were trapped as ZnS) between 3 (week 16 samples) to 6 months (week 0 samples) after sampling. The exact concentration of a 0.1 M Na_2S stock solution used for calibration was first determined by iodometric titration (EPA, 1996; Peiffer and Pecher, 1997). The sulfide concentration in samples was then determined spectrophotometrically with the methylene blue method adapted from Cline (1969). These measurements showed undetectable sulfides, i.e., < 10 μM (maximum limit of detection) in all pore waters. In order to check that this observation was due to the absence of sulfides and not to poor preservation of the samples, we prepared test solutions with variable amounts of Na_2S (80 μM to 4 mM) trapped with Zn acetate in different matrices (ultrapure water or synthetic seawater used for FKS flooding (Table S1)) in glass vials with rubber stoppers (same as for samples) that were flushed with N_2 and stored in the fridge for 6 months. After this period, which corresponds to the maximum storage time of samples, we measured sulfide concentrations corresponding to the expected amounts in these test samples, thus validating sample preservation in these conditions. We therefore consider that our pore waters really did not contain measurable amounts of sulfides. The total alkalinity in pore waters collected after 19 weeks was approximated by analysis of dissolved carbonates and was measured by colorimetric titration using the bromocresol green-methyl red indicator.

HCl extraction of transformed solids

Within one day after the HCl extractions, the supernatants were analyzed for their dissolved Fe(II) and total dissolved Fe by UV-Vis spectrometry as described above. The total concentrations of cations (including Fe and Zn) were then analyzed by ICP-OES, and the ^{57}Fe fraction ($f\,^{57}Fe$, expressed as the counts of ^{57}Fe divided by the sum of counts of isotopes ^{54}Fe, ^{56}Fe, ^{57}Fe and ^{58}Fe) in the

supernatants was determined with an Agilent 8800 Triple Quad ICP-MS (mass spectrometer), using a method described in Schulz et al. (2022).

5. Mineralogical analyses

X-Ray Diffraction

The initial dry sediments and selected transformed Fh-Zn:sediment mixes were ground and deposited with ethanol on Si(711) wafers for the analysis of mineral phases by bulk powder X-ray diffraction (XRD), using a Bruker D8 Advance diffractometer from 10 to 70 ° (2θ) as described above for Fh-Zn. The only difference is that sediments were measured in open air on a rotative stand (30 s^{-1}), while Fh-Zn:sediment samples were analyzed in a N_2-filled dome without rotation, to preserve their oxidation state.

Apart from expected minerals from the sediment (quartz, feldspars, clays), no other crystalline phases associated with transformed Fh-Zn could be detected, as could be expected regarding (i) the high dilution factor of Fh-Zn and its transformation products in the sediment matrix and (ii) the hardly detectable XRD pattern of poorly crystalline ferrihydrite in presence of crystalline phases. This observation confirms the need for ^{57}Fe enrichment in order to detect and characterize Fe mineral products of transformation that are diluted in the sediment. Consequently, no XRD data is shown in the present study.

^{57}Fe Mössbauer spectroscopy

The ^{57}Fe Mössbauer spectroscopy measurements were made in transmission mode with a ^{57}Co source and a constant acceleration drive system over a -12 to 12 mm/s velocity range in a standard setup (WissEl, Wissenschaftliche Elektronik GmbH) equipped with a closed-cycle He cryostat (SHI-850, Janis Research Co.). The experimental spectra were calibrated with an α-Fe(0) foil regularly measured at room temperature. All samples were analyzed at 77 and 5 K, with additional temperatures (140, 60, 50, 40, 30, 20, 10 K) for a selection of samples. Ground samples were homogeneously spread and sealed in Kapton tape, within the glovebox for all air-sensitive samples, and quickly transferred into the cryostat under He atmosphere. The samples were measured for several hours (several days for sediment samples) until a satisfactory signal-to-noise ratio was

achieved. The spectra were calibrated, folded and fitted on the Recoil software (Lagarec and Rancourt, 1998). An extended Voigt-based fitting (xVBF) procedure (Lagarec and Rancourt, 1997) was used to model and quantify the observed spectral features at 77 K. The half-width at half-maximum was fixed to 0.135 mm s^{-1}, corresponding to the value of the inner line broadening of the calibration foil. For 5 K spectra, we applied a Full Static Hamiltonian (FSH) model (Blaes et al., 1985).

Bulk X-Ray Absorption Spectroscopy

Bulk Extended X-Ray Absorption Fine Structure (EXAFS) analysis was performed on selected samples at the Zn and Fe *K*-edges at the BM23 bending magnet beamline of the European Synchrotron Radiation Facility (ESRF, Grenoble, France). For Fe *K*-edge measurements, ground samples were diluted with variable amounts of organic XRF wax to reach optimal absorption coefficients and pressed as 7 mm pellets. The samples were not diluted for Zn *K*-edge analysis. Two to three sample pellets were mounted inside an anaerobic glovebox (Ar atmosphere, ESRF Chemistry Laboratory) on 3D-printed resin sample holders, transferred to the beamline in air-tight containers, and quickly inserted into the beamline liquid He cryostat. All bulk measurements were performed at 10 K to limit potential beam damage, protect samples from air exposure and facilitate data analysis by reducing thermal agitation. The beam energy was selected by a Si(111) double-crystal monochromator, and harmonics were rejected using mirrors. The sample holder was rotated by 45 ° relative to the beam, and a Vortex Si-drift fluorescence detector was placed perpendicular to the beam (45° relative to the sample). Ion chambers were placed before and after the sample in the beam direction to measure X-Ray transmission, with an additional chamber to analyze a reference metal foil (Zn or Fe when appropriate) for calibration in double-transmission setup. Zn *K*-edge EXAFS measurements were performed in fluorescence mode (10 to 37 scans per sample), while Fe *K*-edge EXAFS was done either in transmission or in fluorescence mode (3 to 17 scans per sample).

Micro-X-Ray Fluorescence and micro-X-Ray Absorption Spectroscopy

Two samples (HW 5 ini, initial mix of dry HW sediment and Fh-Zn 5%, and HW 5 16A, reacted for 16 weeks) were chemically probed by micro-X-Ray Fluorescence (μ-XRF) imaging, complemented by micro-X-Ray Absorption Near-Edge Structure (μ-XANES) measurements on selected points, at the BM23 beamline. For these micro-focused measurements, unground sample

powders were spread on carbon tape. All samples were processed inside an anaerobic glovebox and transported to the ESRF in air-tight containers to preserve their oxidation state.

The samples were placed perpendicular to the beam, and the fluorescence detector was placed at 45 ° between the beam and the sample plane. In these conditions, the samples were exposed to air for several hours. The beam was focused to a focal point size of 3-5 µm with two mirrors in Kirkpatrick-Baez (KB) geometry. First, micro-XRF maps were acquired with a beam energy of 12 keV, using 5 µm steps and a counting time of 0.5 s per point. The emission spectrum energy was calibrated using the Zn K_α and Fe K_α and K_β emission lines. The XRF spectra were converted to element-specific fluorescence intensity maps with the PyMca software (Solé et al., 2007) through batch curve fitting of emission lines of elements of interest (including Zn K_α and Fe K_α and K_β). Based on visual evaluation of the chemical maps, selected points were targeted for micro-XANES analyses at the Zn and/or Fe *K*-edges. A few series of micro-XANES spectra were acquired consecutively on the same spot to assess beam-induced damage to the sample (e.g., Lefebvre et al., 2022). No clear evolution of the sample redox and speciation were observed, indicating that the beam-damage was likely negligible in this case (or faster than the acquisition time of the first spectrum, i.e., ~9 min, although this seems unlikely considering the observed wide range of Fe redox).

All XAS spectra were examined, merged, calibrated and normalized using the Athena software (Ravel and Newville, 2005). The XANES and EXAFS spectra were then analyzed by linear combination-least square fitting (further abbreviated as LCF) on Athena, using a large set of reference spectra from our databases covering all expected Zn and Fe species. Figures S5 and S6 show the XAS spectra of all reference compounds which were actually used in LCF of sample data. The XANES spectra were fitted from -20 to + 80 eV relative to E_0 (set for all spectra to 9663 eV for Zn, 7128 eV for Fe), and EXAFS from k 3 to 9 Å^{-1}. Because the micro-XANES spectra could not be precisely energy-calibrated during the measurements, an energy shift (ΔE_0) was allowed to vary during the fitting procedure. For micro-XANES fitting, we tried to minimize the number of references with a maximum of 3, and excluded references contributing less than 10 %, except when required for a reasonable fit.

Because bulk XAS measurements at the Fe *K*-edge probe all Fe present in a sample, as opposed to ^{57}Fe Mössbauer spectroscopy which targets only ^{57}Fe, the signal from the sedimentary Fe in Fh-Zn:sediment mixes contributes a large fraction of the total XAS signal. In the initial Fh-Zn:sediment mixes, this sediment contribution makes up to 30 and 25 % of the Fe content in HW and FKS samples, respectively. When taking into account the significant Fe losses by diffusion

revealed by HCl extraction (see below), the sediment contribution to Fe XAS signal reaches 50-62 % for HW 5 16A, 52-59 % for FKS 5 16A and 45-55% for HW 0.5 16A. In an attempt to better characterize the Fh-Zn transformation products in sample HW 5 16A, we subtracted the contribution of sediment Fe (between 50 and 62 %, approximated here as 55 %) to the total XANES and EXAFS signal before applying the LCF procedure. Results from this attempt are shown in Figures S39 to S41. Although the XANES and EXAFS fits are not ideal and should thus be considered with caution, they provide comparable and consistent results.

Given the low sediment Zn concentrations and the large Zn contents in added Fh-Zn, the contribution from sedimentary Zn to the total Zn signal is almost negligible in samples HW 5 16A and FKS 5 16A (3 and 2 % respectively), and relatively low in HW 0.5 16A (23 %, not accounting for Zn losses by diffusion).

Some micro-XANES spectra could not be perfectly fit with the available set of reference spectra. This is particularly the case at the Zn *K*-edge for particles consisting of a mix of Fh-Zn 5 % and franklinite ($ZnFe_2O_4$) (Figure S48e), and at the Fe *K*-edge for particles of siderite ($FeCO_3$) (Figure S44d). Given the unique features in the first oscillations of the XANES spectra of these reference compounds (Figures S5 and S6), we are still confident in the identification of such phases. We thus attribute the slight micro-XANES misfits to differences in the crystallinity and purity of these phases between the model compounds and our samples.

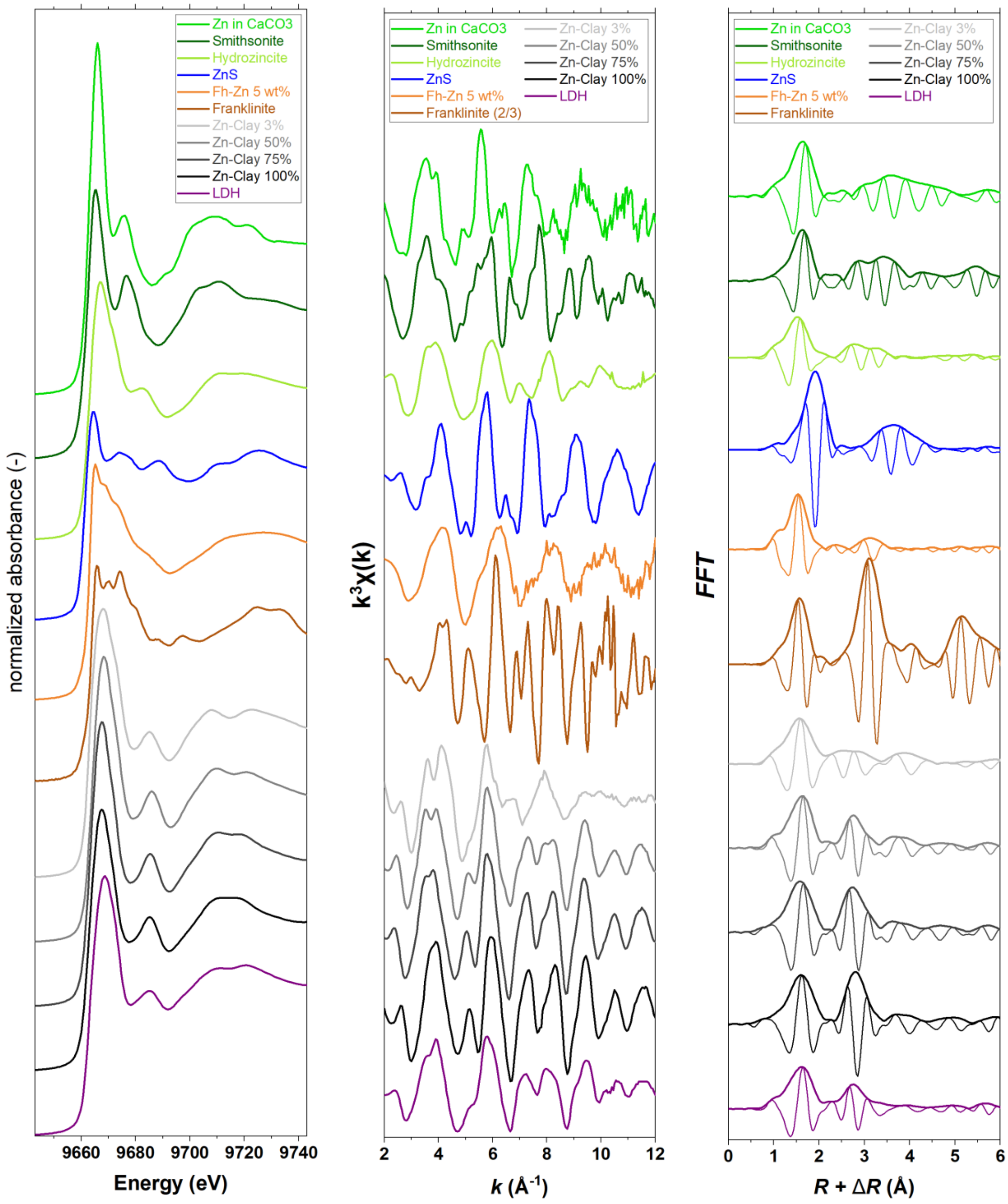

Figure S5 – Zn K-edge XANES and EXAFS spectra of Zn reference compounds used for LCF of bulk and micro-XANES data. (Left) XANES spectra as a function of energy. (Middle) EXAFS spectra (k^3-weighted chi(k) function) as a function of wavenumber k. The EXAFS spectrum of franklinite was reduced (2/3 of actual amplitude) for plotting purposes. (Right) Fast Fourier Transforms (FFT) of the EXAFS data (magnitude and real part) as function of radial distance to the absorber atom, uncorrected for phase shift.

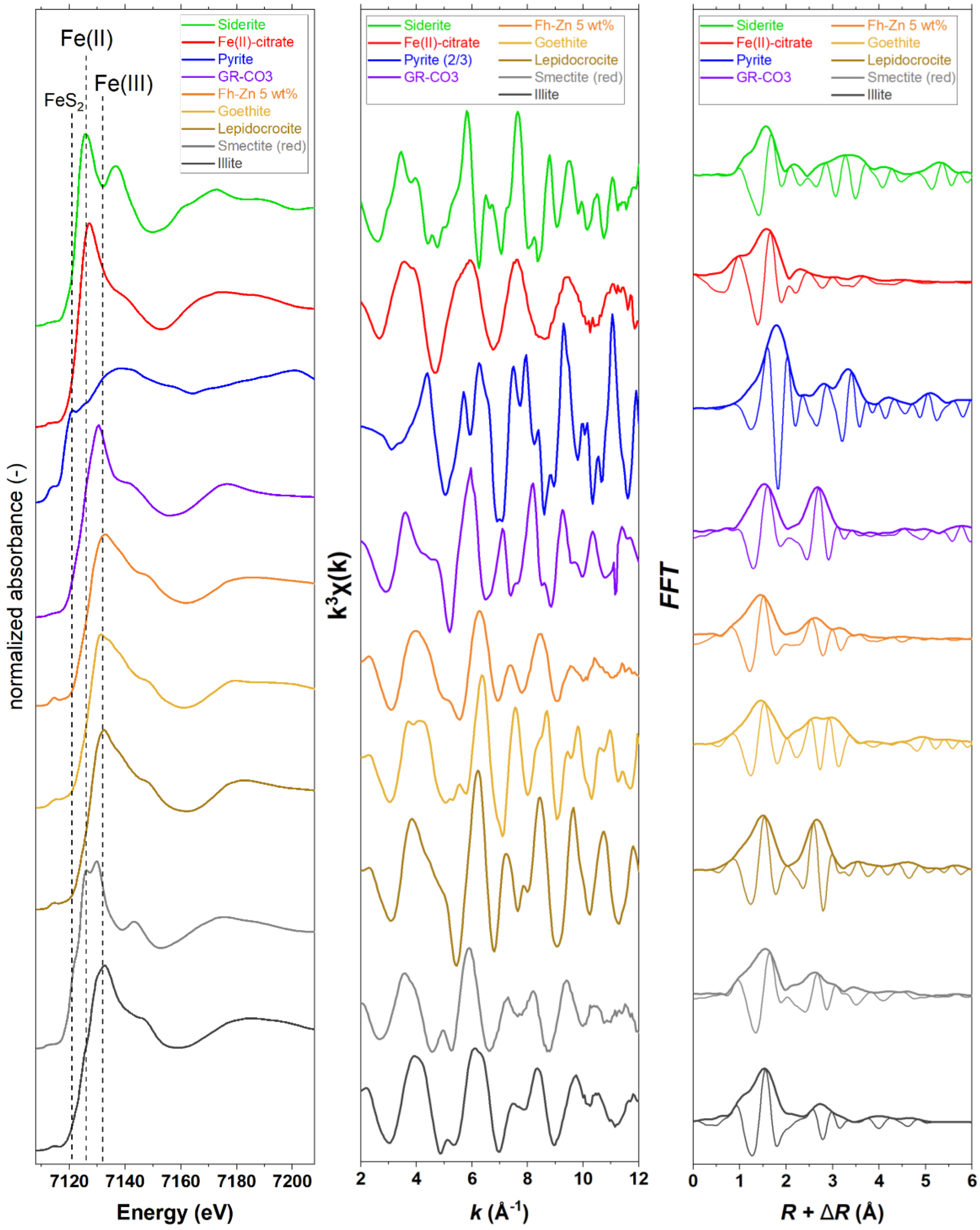

Figure S6 – Fe K-edge XANES and EXAFS spectra of Fe reference compounds used for LCF of bulk and micro-XANES data. (Left) XANES spectra as a function of energy. Vertical dashed lines indicate the whiteline peak positions of the main Fe redox states (Fe in pyrite FeS_2 at ~7121 eV, Fe(II) at ~7126 eV and Fe(III) at ~7132 eV). (Middle) EXAFS spectra (k^3-weighted chi(k) function) as a function of wavenumber k. The EXAFS spectrum of pyrite was reduced (2/3 of actual amplitude) for plotting purposes. (Right) Fast Fourier Transforms (FFT) of the EXAFS data (magnitude and real part) as function of radial distance to the absorber atom, uncorrected for phase shift.

6. Physico-chemical parameters of the mesocosms

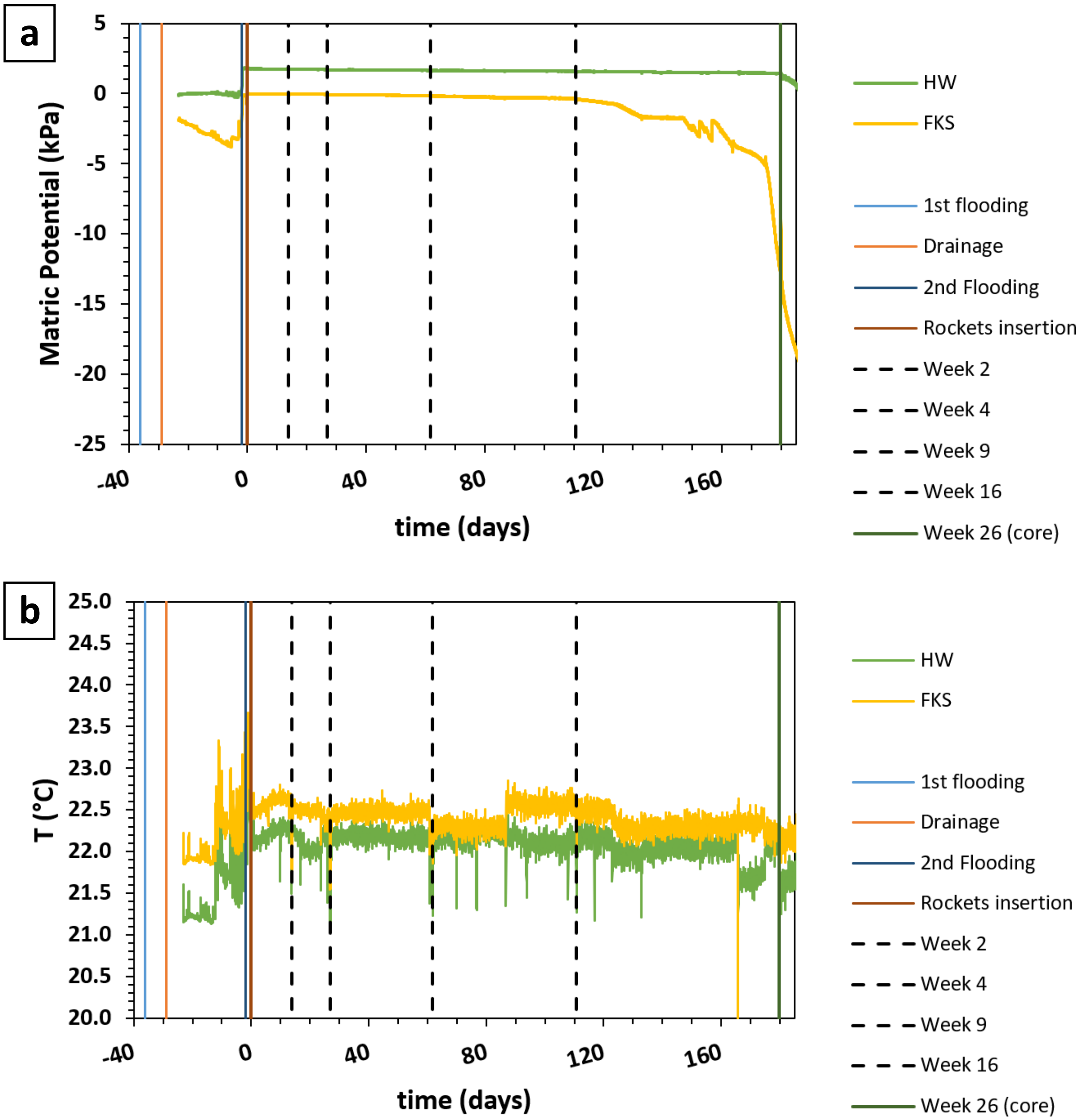

Figure S7 – Evolution of the main physico-chemical parameters during the incubation in the HW (green) and FKS (yellow) mesocosms: (a) matric potential, (b) temperature, (c) pH and (d) redox potential Eh. The occurrence of main events is represented by vertical lines: initial flooding with double-deionized water (light blue), start of the drainage period (light orange), second flooding with diluted artificial seawater (dark blue), insertion of the samples (dark red), four sample collection steps at 2, 4, 9 and 16 weeks (dashed black) and sampling of sediment cores for the evaluation of Zn and Fe diffusion after 26 weeks (dark green).

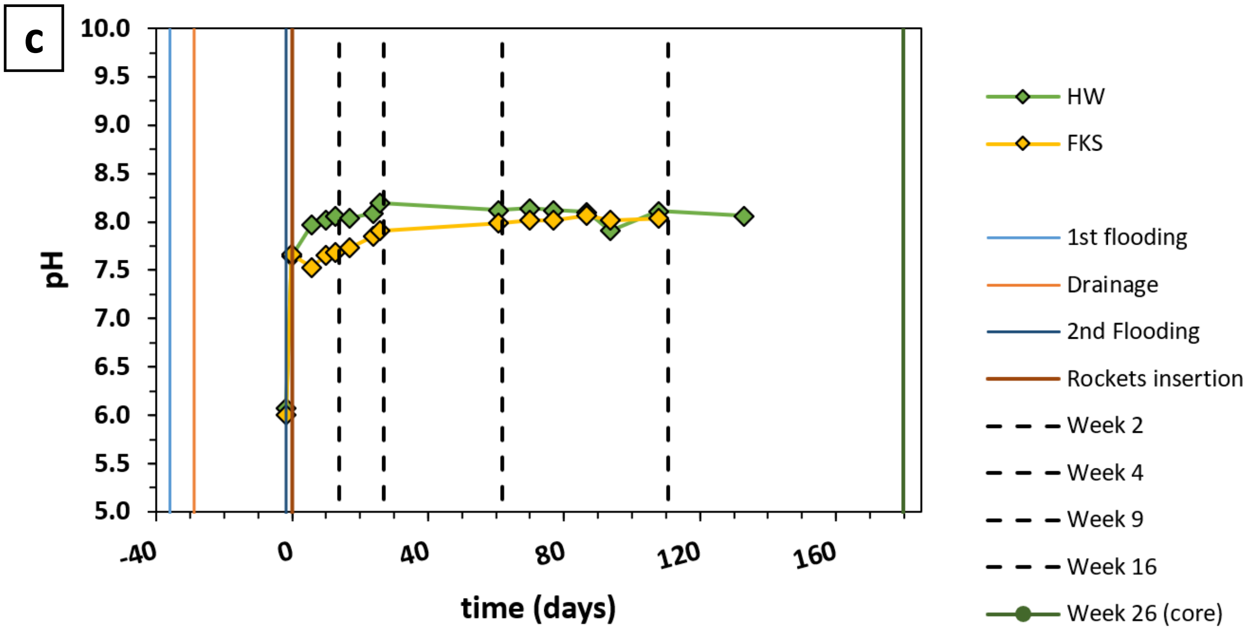

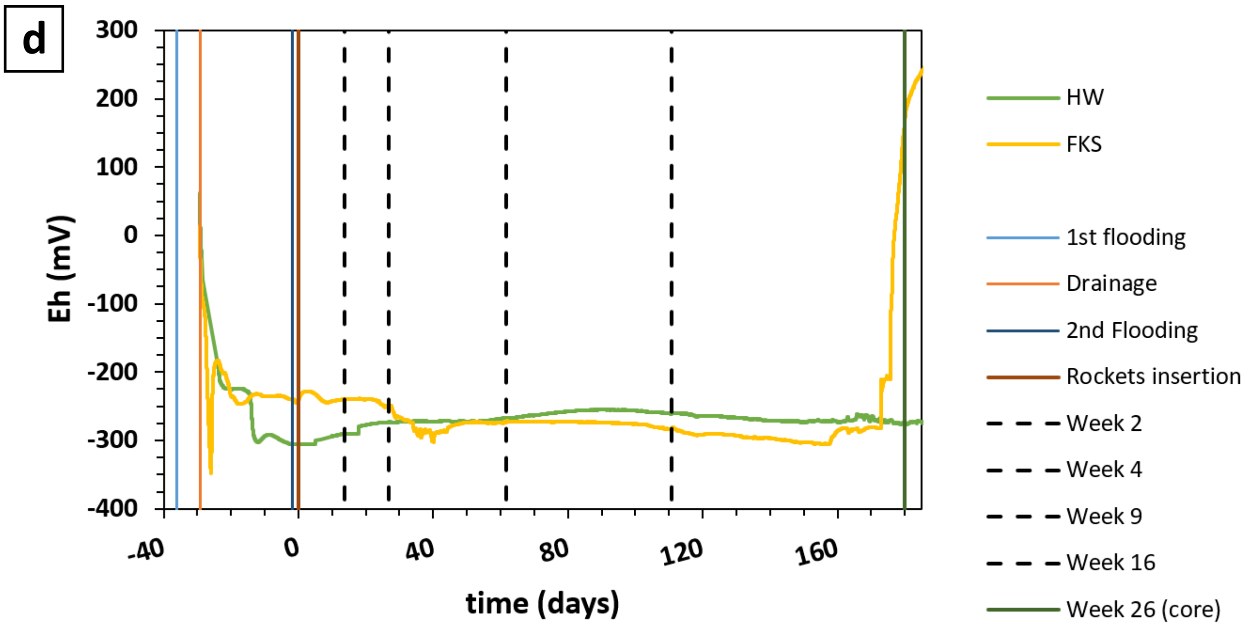

Figure S7 – continued.

7. Sediment composition

Mössbauer spectra were acquired at 77 and 5 K for both initial dry sediments ("sed ini") and sediments reacted in the mesocosms for 18 weeks ("sed 18w"). Only 77 K spectra and fitting parameters are shown here in Figure S8 and Table S4; 5 K spectra showed similar Fe(II) and Fe(III) doublets, partly transformed into a mixed-valence disordered Fe phase (collapsed feature) and, only in dry sediments, an additional Fe(III) sextet (about 16-17 % of total Fe) corresponding to poorly crystalline lepidocrocite.

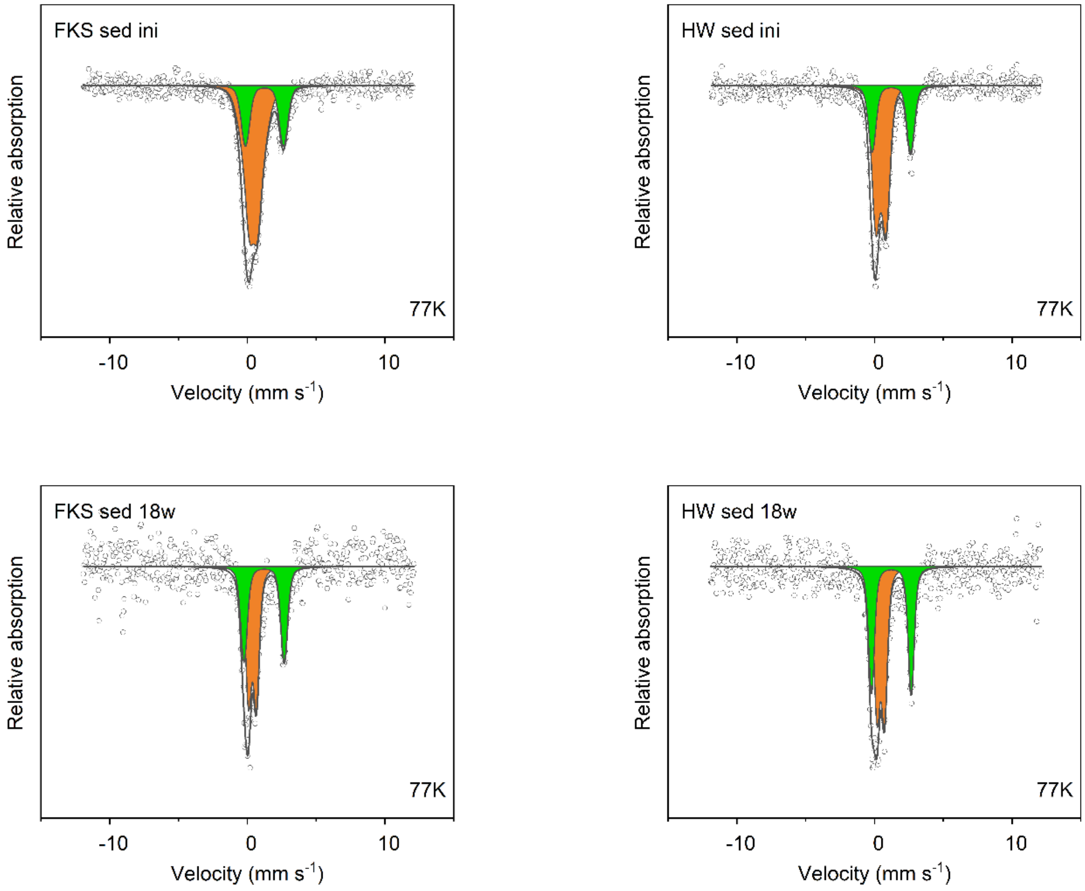

Figure S8 – Mössbauer spectra at 77 K of initial dry sediments (top) and reduced, reacted sediments (bottom) from Friedrichskoog (FKS, left) and Hollerwettern (HW, right). The experimental data is shown as open circles, the calculated fit as a black line, and the fitted components as filled areas: orange for the Fe(III) doublet and green for the Fe(II) doublet.

*Table S4 – Fitting parameters (xVBF model) of Mössbauer spectra acquired at 77 K for dry and reduced sediments. CS: center/isomer shift, QS: quadrupole splitting, σ_{QS}: standard deviation of QS, χ^2_R: reduced chi-square. Uncertainties on the last digits, calculated from the covariance matrix, are given in brackets. * indicates a fixed parameter.*

Sample	Doublet	Contribution (%)	CS (mm/s)	QS (mm/s)	σ_{QS} (mm/s)	χ^2_R
**FKS sed ini**	Fe(II)-D1	27.5(17)	1.254(38)	2.769(73)	0.5*	0.59
	Fe(III)-D2	72.5(17)	0.447(23)	0.563(57)	0.41(11)	
**FKS sed 18w**	Fe(II)-D1	44.8(45)	1.224(44)	2.919(88)	0.36(11)	0.60
	Fe(III)-D2	55.2(45)	0.376(40)	0.568(54)	0.3*	
**Fe(II) difference 18w - ini (%)**		**17.3(48)**				
**HW sed ini**	Fe(II)-D1	31.9(21)	1.231(32)	2.793(65)	0.428(72)	0.68
	Fe(III)-D2	68.1(21)	0.473(20)	0.732(30)	0.455(41)	
**HW sed 18w**	Fe(II)-D1	46.7(32)	1.229(26)	2.879(53)	0.291(64)	0.63
	Fe(III)-D2	53.3(32)	0.456(32)	0.543(41)	0.3*	
**Fe(II) difference 18w - ini (%)**		**14.8(38)**				

The same dry and reduced sediments were also analyzed by Fe *K*-edge XANES and EXAFS. The results of LCF are shown in Figures S9 to S11 and Table S5 and indicate the predominance (63-90 %) of Fe-bearing clays (smectite and illite), with lower proportions of Fe (oxyhydr-)oxides (ferrihydrite and/or lepidocrocite) and minor amounts (4-10 %) of pyrite.

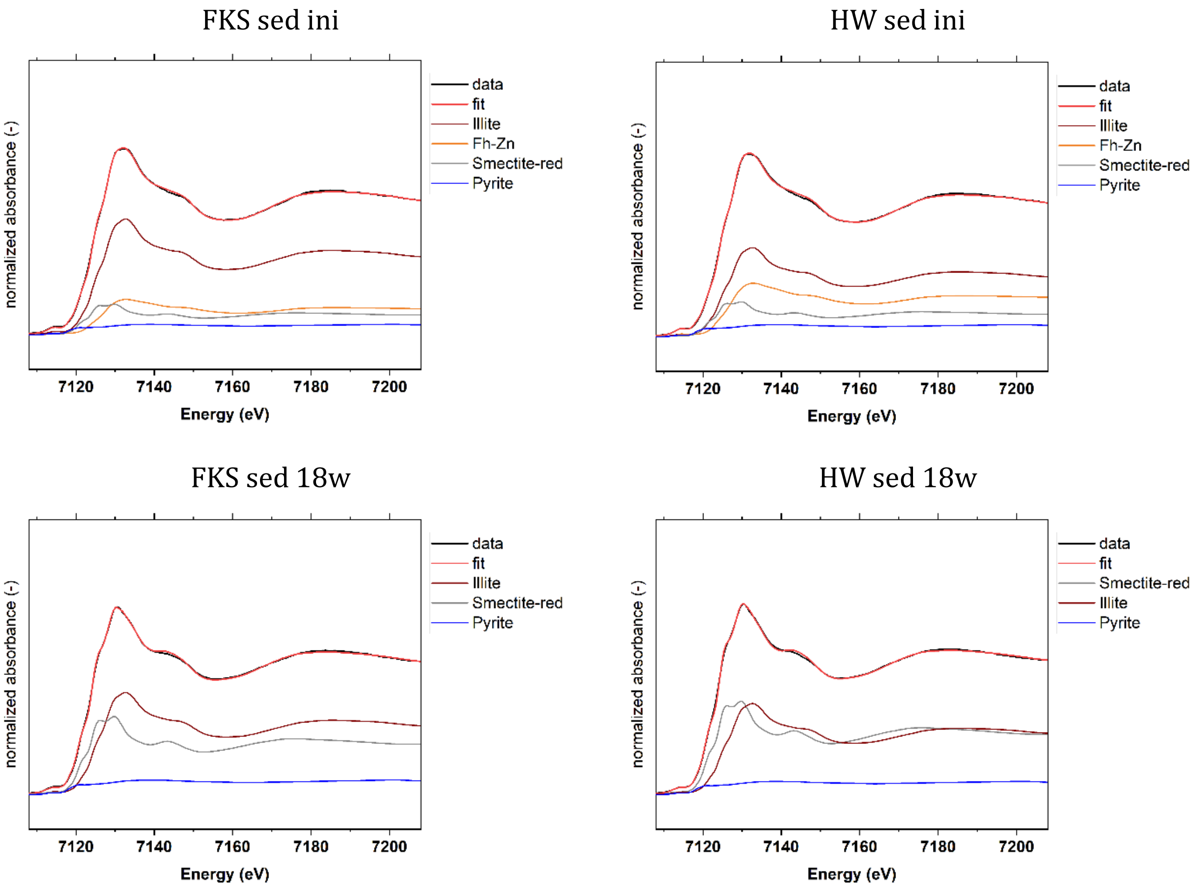

Figure S9 – Fe K-edge XANES spectra of initial dry sediments (top) and reduced, reacted sediments (bottom) from Friedrichskoog (FKS, left) and Hollerwettern (HW, right). The experimental data and corresponding LC fits are shown in black and red, respectively. The weighted spectra of fitted reference compounds are shown below in dark red for illite, gray for reduced smectite, blue for pyrite, and orange for ferrihydrite (Fh-Zn).

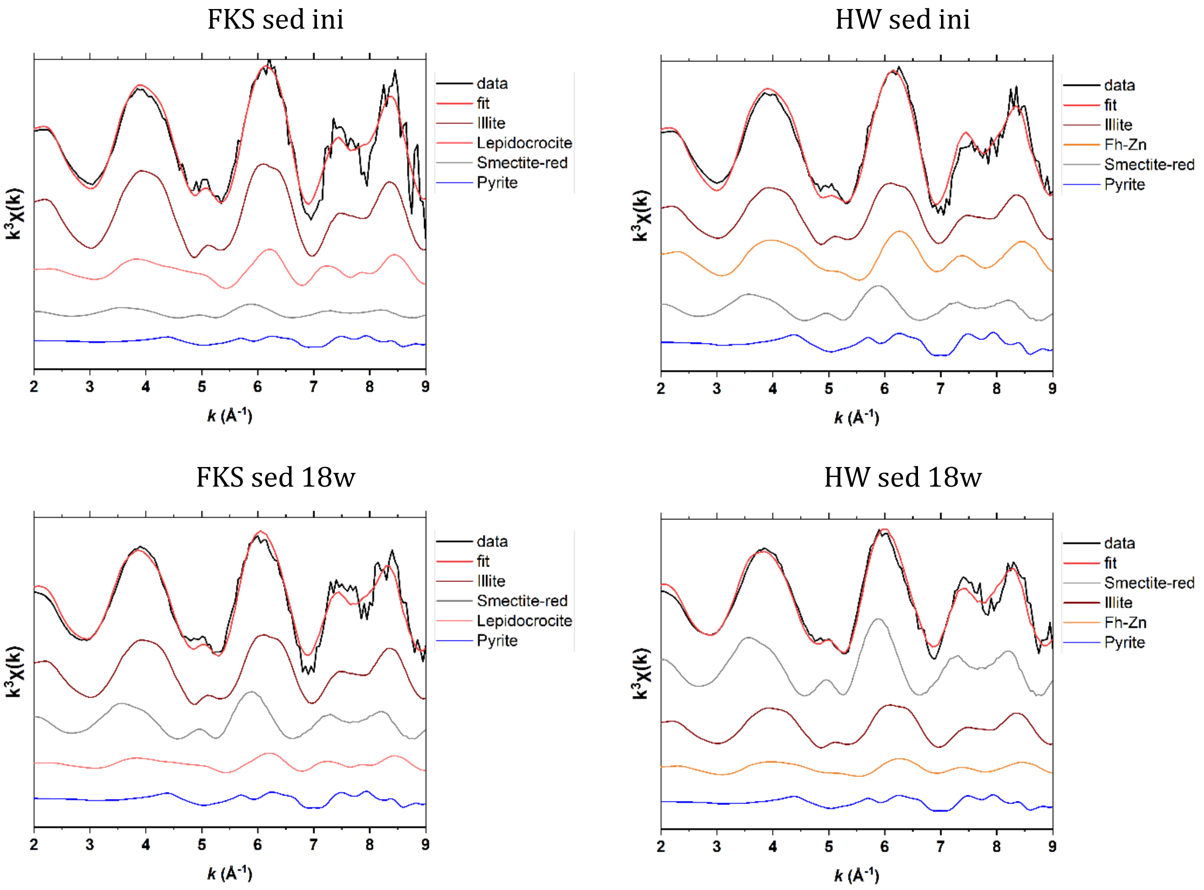

Figure S10 – Fe K-edge EXAFS spectra of initial dry sediments (top) and reduced, reacted sediments (bottom) from Friedrichskoog (FKS, left) and Hollerwettern (HW, right). The experimental data and corresponding LC fits are shown in black and red, respectively. The weighted spectra of fitted reference compounds are shown below in dark red for illite, gray for reduced smectite, blue for pyrite, pink for lepidocrocite and orange for ferrihydrite (Fh-Zn).

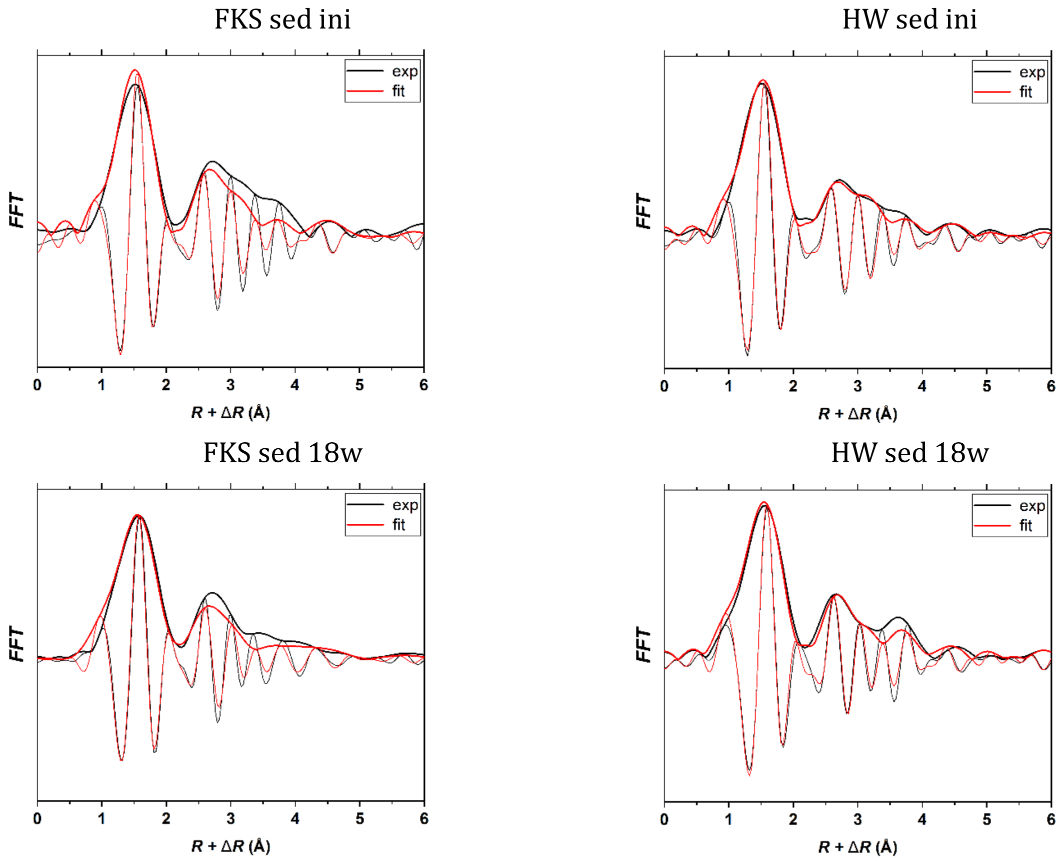

Figure S11 – Fast Fourier Transforms (FFT) of Fe K-edge EXAFS spectra of initial dry sediments (top) and reduced, reacted sediments (bottom) from Friedrichskoog (FKS, left) and Hollerwettern (HW, right). The experimental data and corresponding LC fits are shown in black and red, respectively.

Table S5 – Fe K-edge XAS fitting parameters of dry and reduced sediments. Ill: illite, Smec: smectite (reduced), Py: pyrite, Fh: Fh-Zn 5%, Lp:lepidocrocite. %Fe(II): percentage of Fe(II) over total Fe, calculated from the weighted Fe(II) content of fitting components. Rf: R-factor, χ^2_R: reduced chi-square.

		Components						Fit parameters	
**Sample**	**Fit**	**Ill.**	**Smec.**	**Py.**	**Fh**	**Lp**	**% Fe(II)**	**R_f**	**χ^2_R**
**FKS sediment**	**XANES**	58(2)	15(1)	7.3(4)	19(2)	-	34	$2.0 .10^{-4}$	$3.5 .10^{-5}$
**ini**	**EXAFS**	70(7)	10(4)	4(2)	-	15.8(4)	27	$9.4 .10^{-2}$	0.91
**FKS sediment**	**XANES**	51.3(4)	38.7(4)	10.0(4)	-	-	58	$3.2 .10^{-4}$	$5.3 .10^{-5}$
**18w**	**EXAFS**	52(5)	35(3)	5(1)	-	8(3)	50	$6.0 .10^{-2}$	0.45
**HW sediment**	**XANES**	45(2)	17.5(6)	8.4(4)	29(2)	-	34	$2.2 .10^{-4}$	$3.8 .10^{-5}$
**ini**	**EXAFS**	40(7)	23(4)	6(1)	31(7)	-	37	$11 .10^{-2}$	0.92
**HW sediment**	**XANES**	45.2(4)	45.7(4)	9.0(4)	-	-	63	$3.6 .10^{-4}$	$5.9 .10^{-5}$
**18w**	**EXAFS**	30(6)	54(3)	5(1)	12(5)	-	64	$7.8 .10^{-2}$	0.53

8. HCl extraction of sediments

Table S6 – Results of 0.5 M HCl extractions of dry (ini) and reduced (18w) sediments, with proportions of Fe extracted compared to total initial Fe, and Fe(II)-Fe(III) proportions of HCl-extracted Fe. Uncertainties on the last digits are given in brackets.

Sample	% of total Fe extracted by HCl	Redox of HCl-extracted Fe	
		Fe(II) (%)	Fe(III) (%)
**FKS sed ini**	22.8(7)	22.8(18)	77.2(61)
**FKS sed 18w**	24.0(37)	94.5(56)	5.5(56)
**HW sed ini**	35.2(10)	12.5(2)	87.5(7)
**HW sed 18w**	32.6(57)	85(10)	15(10)

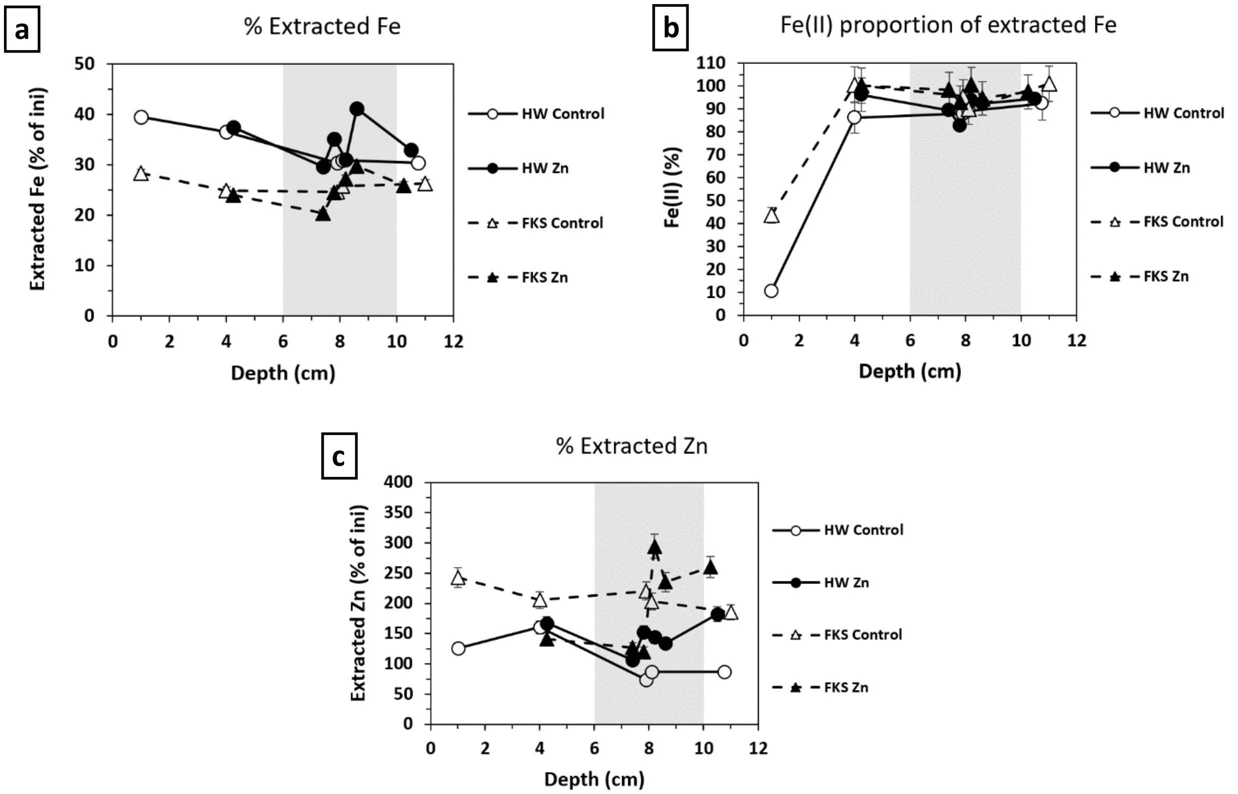

Figure S12 – Results from HCl extractions of sediments subsamples from Control Cores (open symbols) and Zn Cores (filled symbols) of HW and FKS mesocosms (circles and triangles respectively). (a) Proportion of HCl-extracted Fe relative to total solid-phase Fe (measured by XRF) as a function of depth; (b) Fe(II) proportion of the extracted Fe; (c) proportion of HCl-extracted Zn relative to total solid-phase Zn as measured by XRF. The sample depth range is indicated by a grey area. For visualization purposes, the four sediment concentric subsamples collected at 6.5-9.5 cm around the mesh bag in Zn cores (from outside to inside: OUT, MID, IN, RW) are represented at increasing depths, i.e., OUT at 7.4 cm and RW at 8.6 cm.

Chemical extractions with 0.5 M HCl were performed on all sediments subsampled from Control Cores and Zn Cores of HW and FKS mesocosms (Figure S3), with results shown in Figure S12. Some similar patterns are observed between both Control Cores, with different magnitudes. The percentage of Fe extracted by 0.5 M HCl is slightly decreasing with depth, with higher extracted Fe in the upper sediments (Figure S12a). More Fe is extracted from HW sediments (30-40 %) than from FKS sediments (25-28 %). Fe extracted from the upper sediments is predominantly Fe(III) – 10 % in HW, 44 % in FKS (Figure S12b) – which is consistent with the orange color of the top 2 cm, which are exposed to atmospheric oxygen diffusion. Below this top oxidized layer, Fe is dominantly present as Fe(II) with proportions ranging between 86 and 100 %. The trend of Zn extracted proportions is more variable with depth (Figure S12c). An unexpected observation is that the measured amounts of extracted Zn (measured by ICP-OES in the extracted solutions) often exceed the maximal value calculated using the Zn solid concentration measured by XRF. We attribute these discrepancies to uncompensated large analytical uncertainties due to the low Zn concentrations in the extracted solution, and/or to an undetected Zn contamination of the HCl solutions (not seen in analytical blanks). Consequently, only large variations in the extracted Zn proportions can be qualitatively discussed.

In contrast to the Control Cores, the Zn cores from HW and FKS mesocosms show a clear increase in extracted Fe and Zn proportions at sample depth (grey areas in Figure S12), with higher proportions in sediments in contact with the mesh bag (samples 6.5-9.5 cm RW). This likely indicates diffusion of Fe and Zn from the mesh bag containing Fh-Zn 5 %. The Fe(II)-dominated oxidation state of this additional Fe is indistinguishable from sediment Fe (Figure S12b).

The diffusion of Fe from the mesh bag is confirmed by Fe isotope measurements ($f\,^{57}$Fe) of the HCl solutions that show a clear enrichment in ^{57}Fe in Zn cores sediment close to the mesh bag, with $f\,^{57}$Fe ratios up to 6 and 8 % in HW and FKS samples 6.5-9.5 cm RW (instead of 2.1 % for natural abundance). A rough estimation of the diffused ^{57}Fe amounts (lost from the mesh bag) was obtained using sediment volumes and densities and ^{57}Fe/Fe contents. This estimation could then be compared to the amounts of ^{57}Fe lost from the Fh-Zn:sediment mix, measured with Fe isotopes (see Figure S20 below). An estimated 1.1 ± 0.5 mg of ^{57}Fe was lost in HW Zn Core, while isotope measurements on the Fh-Zn:sediment mix show a ^{57}Fe loss of 0.94 ± 0.02 mg. In FKS, the diffused ^{57}Fe is estimated at 1.4 ± 0.5 mg, while the measured amount of ^{57}Fe lost from the mesh bag is 0.80 ± 0.03 mg of ^{57}Fe. Although these estimations show high uncertainties (which are likely not all taken into account), they are on the same order of magnitude and thus confirm the likelihood of a strong Fe (and Zn) diffusion out of the Fh-Zn:sediment mixes during the mineral transformation.

9. Pore waters composition

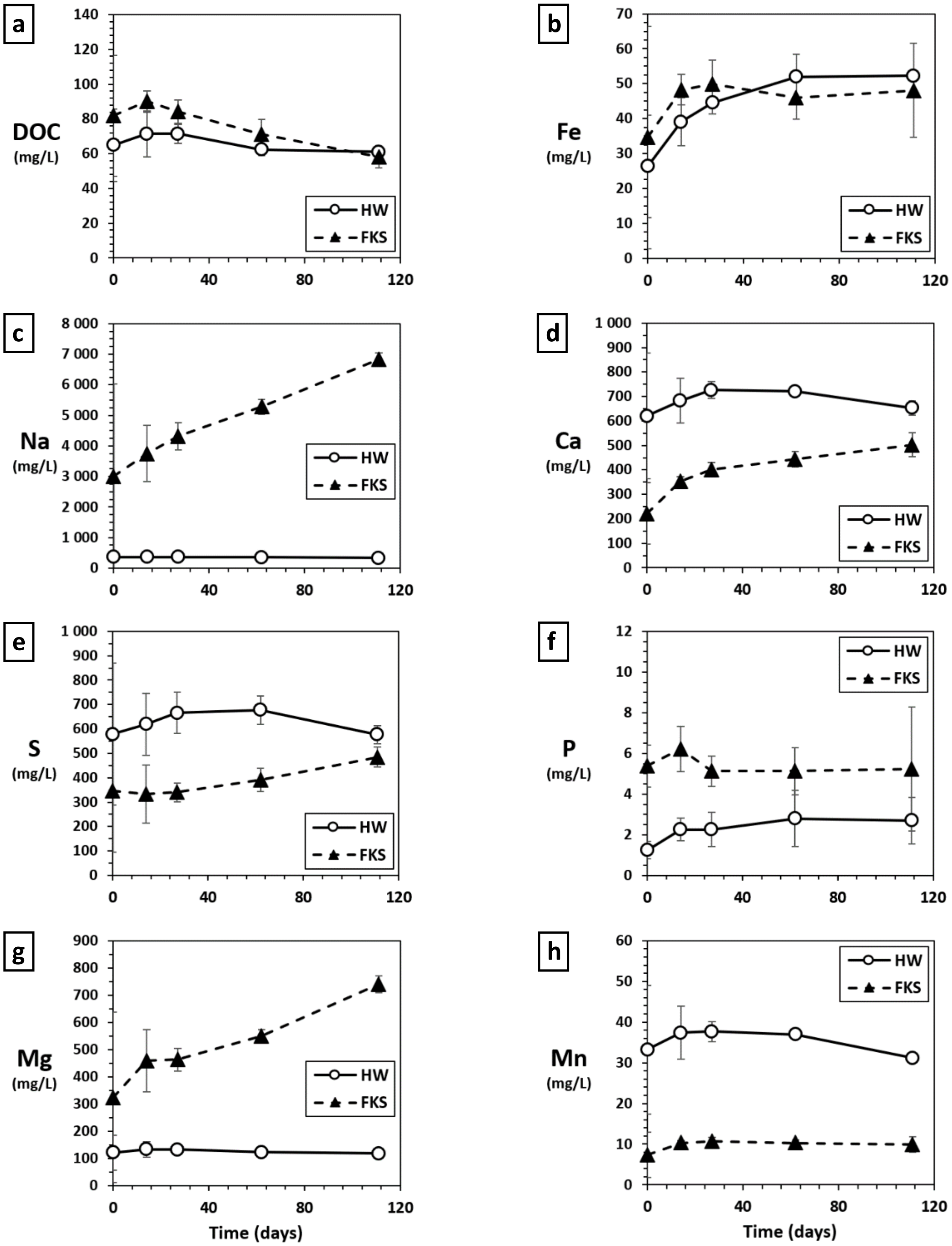

Figure S13 – Evolution over the incubation of the pore water concentrations of (a) Dissolved Organic Carbon (DOC), (b) total Fe, (c) Na, (d) Ca, (e) S, (f) P, (g) Mg and (h) Mn. Pore water samples from the HW mesocosm are shown with open circles and solid lines, samples from FKS with triangles and dashed lines. The error bars show 2SD uncertainties from triplicate pore water samples.

After 16 weeks, dissolved Fe(II) concentrations in the pore waters reached 52 ± 2 mg/L in the HW mesocosm and 53 ± 9 mg/L in FKS (Figure 1). As calculated in Table S4, 15 % of total solid-phase Fe was reduced to Fe(II) after 18 weeks ("available Fe") in HW (17 % in FKS). This corresponds to about 1.6 g/kg "available Fe(II)" in HW sediment (1.4 g/kg in FKS). Using the sediment water contents (28 and 23 % in HW and FKS respectively) and densities (DBD of 1.27 and 1.40 g/cm^3 respectively, see above), we can calculate the fraction of solid-phase available Fe(II) that was dissolved in the pore water after 16-18 weeks. In the HW mesocosm, pore water Fe(II) corresponds to ~1.2 % of solid-phase available Fe(II); in FKS, this proportion equals ~1.1 %.

10. Characterization of initial Fh-Zn:sediment mixes

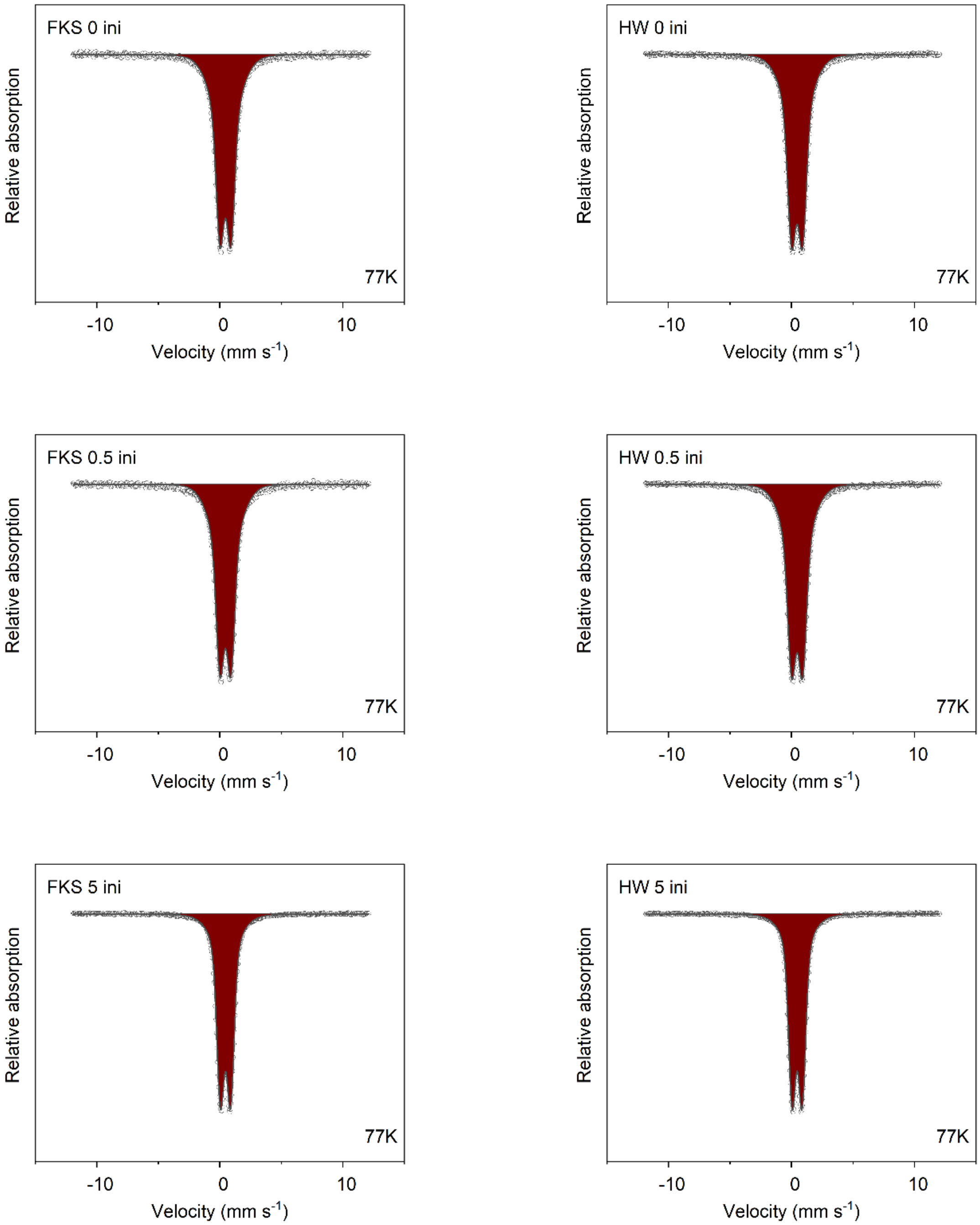

Figure S14 – Mössbauer spectra at 77 K of initial (untransformed) Fh-Zn samples initially containing 0, 0.5 and 5 wt% Zn, mixed with sediments from Friedrichskoog (FKS 0, FKS 0.5 and FKS 5, left) and Hollerwettern (HW 0, HW 0.5 and HW 5, right). The experimental data is shown as open circles, the calculated fit as a black line, and the fitted components as filled areas: dark red for the ferrihydrite Fe(III) doublet, gray for the collapsed feature.

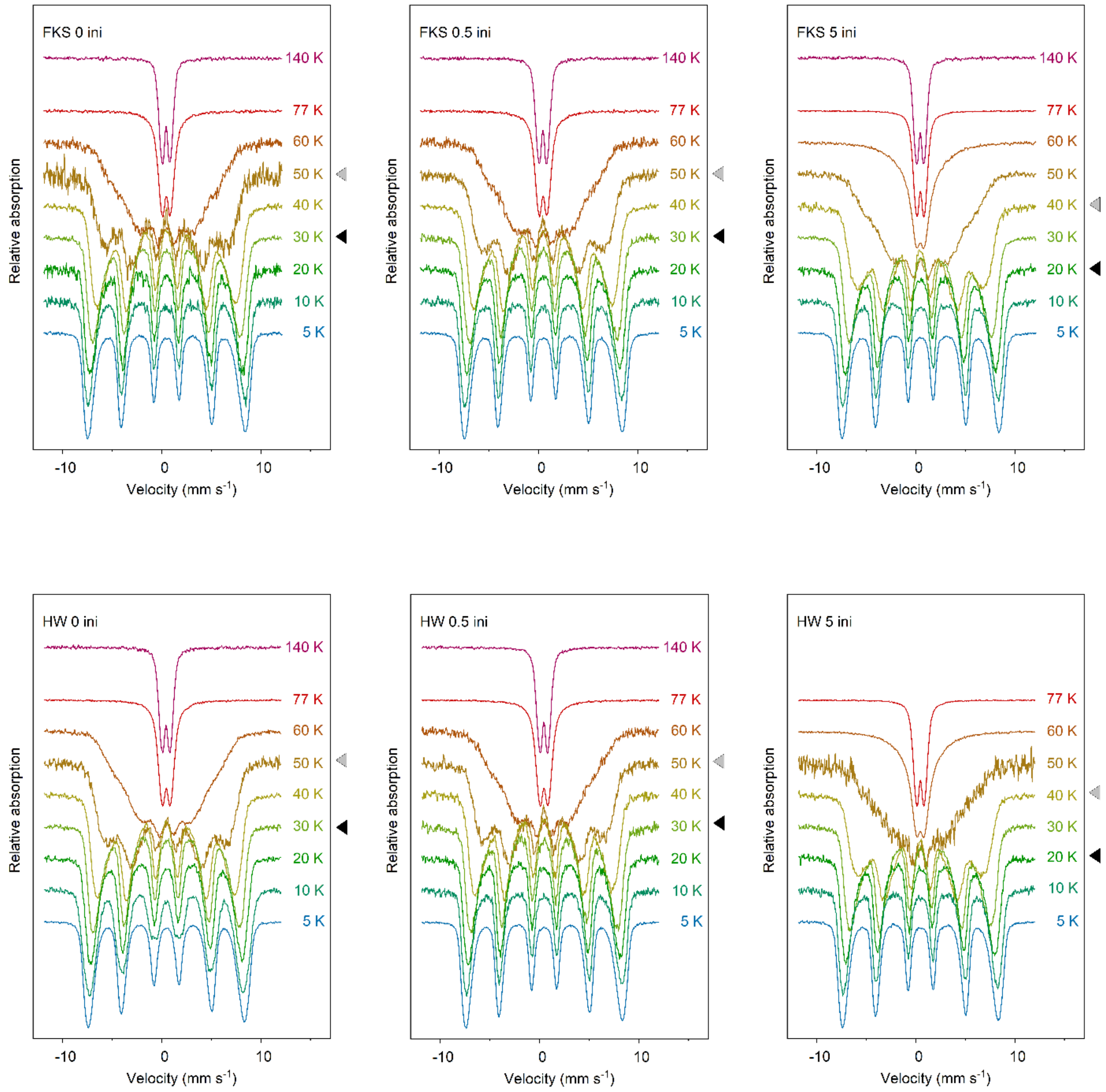

Figure S15 – Temperature profiles of Mössbauer spectra from 140 to 5 K of initial (untransformed) Fh-Zn samples initially containing 0, 0.5 and 5 wt% Zn, mixed with sediments from Friedrichskoog (FKS 0, FKS 0.5 and FKS 5, top) and Hollerwettern (HW 0, HW 0.5 and HW 5, bottom). Each temperature of acquisition is represented by a given color, grading from purple (140 K) to blue (5 K). Arrows on the right-hand side indicate the temperature at which a sextet appears (gray arrow) and is fully ordered (black arrow). Note that no spectrum was acquired at 140 K for the sample HW 5 ini.

Table S7 – Fitting parameters (xVBF model) of Mössbauer spectra acquired at 140 K and 77 K for initial dry Fh-Zn:sediment mixes. As the contribution of sediment Fe to the Mössbauer signal is negligible (< 1 %), the fit parameters were averaged for FKS and HW mixes with the same Fh-Zn and same temperature. CS: center/isomer shift, QS: quadrupole splitting, σ_{QS}: standard deviation of QS, χ^2_R: reduced chi-square. Uncertainties on the last digits, from the covariance matrix, are given in brackets.

Sample	T	Component	Contribution (%)	CS (mm/s)	QS (mm/s)	σ_{QS} (mm/s)	χ^2_R
**FKS 0 ini**		**Fe(III) doublet**	100	0.436(3)	0.911(5)	0.52(1)	
(Fh-Zn 0 % in	**140 K**	*comp. 1*	82(3)	*0.436(3)*	0.845(5)	0.432(10)	1.00
FKS sediment)		*comp. 2*	18(3)	*0.436(3)*	1*	1*	
**FKS 0 ini**		**Fe(III) doublet**	100	0.455(3)	1.269(7)	1.03(2)	
(Fh-Zn 0 % in	**77 K**	*comp. 1*	59(1)	*0.455(3)*	*0.887(5)*	*0.525(8)*	1.02
FKS sediment)		*comp. 2*	41(1)	*0.455(3)*	*1**	*2**	
**HW 0 ini**		**Fe(III) doublet**	100	0.433(3)	0.923(6)	0.56(1)	
(Fh-Zn 0 % in	**140 K**	*comp. 1*	76(3)	*0.433(3)*	*0.833(5)*	*0.453(10)*	0.83
HW sediment)		*comp. 2*	24(3)	*0.433(3)*	*1**	*1**	
**HW 0 ini**		**Fe(III) doublet**	100	0.456(2)	1.237(4)	1.006(7)	
(Fh-Zn 0 % in	**77 K**	*comp. 1*	*62.7(6)*	*0.456(2)*	*0.882(3)*	*0.547(4)*	2.12
HW sediment)		*comp. 2*	*37.3(6)*	*0.456(2)*	*1**	*2**	
**Average**	**140 K**	**Fe(III) doublet**	100	0.435(4)	0.917(8)	0.54(1)	
**Fh-Zn 0 %**	**77 K**	**Fe(III) doublet**	100	0.456(4)	1.253(8)	1.02(2)	
**FKS 0.5 ini**		**Fe(III) doublet**	100	0.432(3)	0.929(5)	0.55(1)	
(Fh-Zn 0.5 % in	**140 K**	*comp. 1*	*76(3)*	*0.432(3)*	*0.845(5)*	*0.432(10)*	0.85
FKS sediment)		*comp. 2*	*24(3)*	*0.432(3)*	*1**	*1**	
**FKS 0.5 ini**		**Fe(III) doublet**	100	0.456(3)	1.299(7)	1.06(2)	
(Fh-Zn 0.5 % in	**77 K**	*comp. 1*	*56(1)*	*0.456(3)*	*0.885(5)*	*0.557(8)*	1.44
FKS sediment)		*comp. 2*	*44(1)*	*0.456(3)*	*1**	*2**	
**HW 0.5 ini**		**Fe(III) doublet**	100	0.432(2)	0.935(4)	0.57(1)	
(Fh-Zn 0.5 % in	**140 K**	*comp. 1*	*73(2)*	*0.432(2)*	*0.841(4)*	*0.439(8)*	1.17
HW sediment)		*comp. 2*	*27(2)*	*0.432(2)*	*1**	*1**	
**HW 0.5 ini**		**Fe(III) doublet**	100	0.455(2)	1.261(4)	1.02(1)	
(Fh-Zn 0.5 % in	**77 K**	*comp. 1*	*60.3(6)*	*0.455(2)*	*0.889(3)*	*0.544(5)*	2.02
HW sediment)		*comp. 2*	*39.7(6)*	*0.455(2)*	*1**	*2**	
**Average**	**140 K**	**Fe(III) doublet**	100	0.432(4)	0.932(6)	0.56(1)	
**Fh-Zn 0.5 %**	**77 K**	**Fe(III) doublet**	100	0.456(4)	1.280(8)	1.04(2)	
**FKS 5 ini**		**Fe(III) doublet**	100	0.438(2)	0.873(4)	0.52(1)	
(Fh-Zn 5 % in	**140 K**	*comp. 1*	*81(3)*	*0.438(2)*	*0.796(4)*	*0.421(8)*	0.91
FKS sediment)		*comp. 2*	*19(3)*	*0.438(2)*	*1**	*1**	
**FKS 5 ini**		**Fe(III) doublet**	100	0.4584(9)	1.024(1)	0.812(3)	
(Fh-Zn 5 % in	**77 K**	*comp. 1*	*80.3(4)*	*0.4584(9)*	*0.818(1)*	*0.479(2)*	3.49
FKS sediment)		*comp. 2*	*19.7(4)*	*0.4584(9)*	*1**	*2**	
**HW 5 ini**		**Fe(III) doublet**	100	0.4565(9)	1.005(1)	0.793(3)	
(Fh-Zn 5 % in	**77 K**	*comp. 1*	*81.6(4)*	*0.4565(9)*	*0.811(1)*	*0.472(2)*	3.45
HW sediment)		*comp. 2*	*18.4(4)*	*0.4565(9)*	*1**	*2**	
**Average**	**140 K**	**Fe(III) doublet**	100	0.438(2)	0.873(4)	0.52(1)	
**Fh-Zn 5 %**	**77 K**	**Fe(III) doublet**	100	0.457(1)	1.015(1)	0.803(4)	

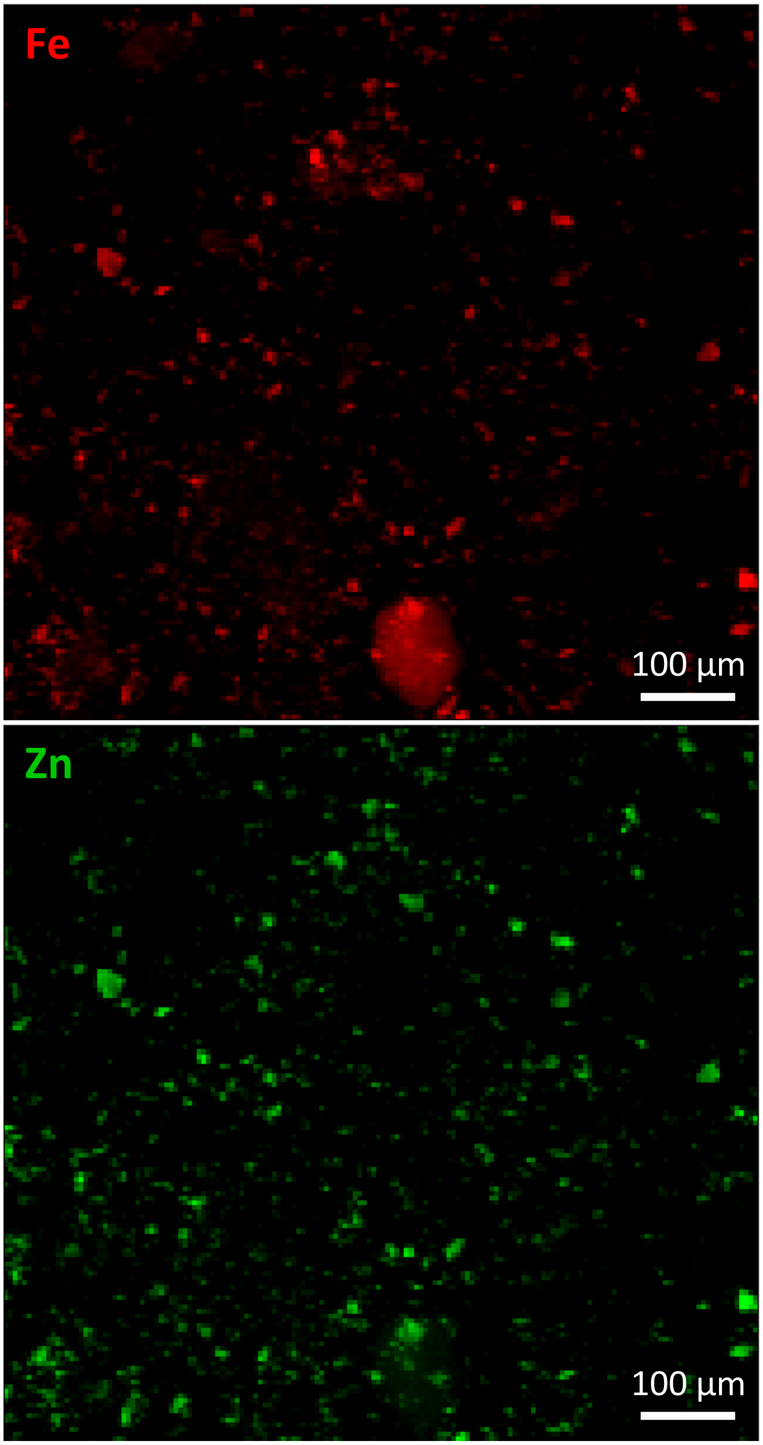

Figure S16 – Micro-X-ray-fluorescence maps of the initial Fh-Zn:sediment mix with 5 wt% Zn and HW sediment (HW 5 ini): (top) Fe in red, (bottom) Zn in green.

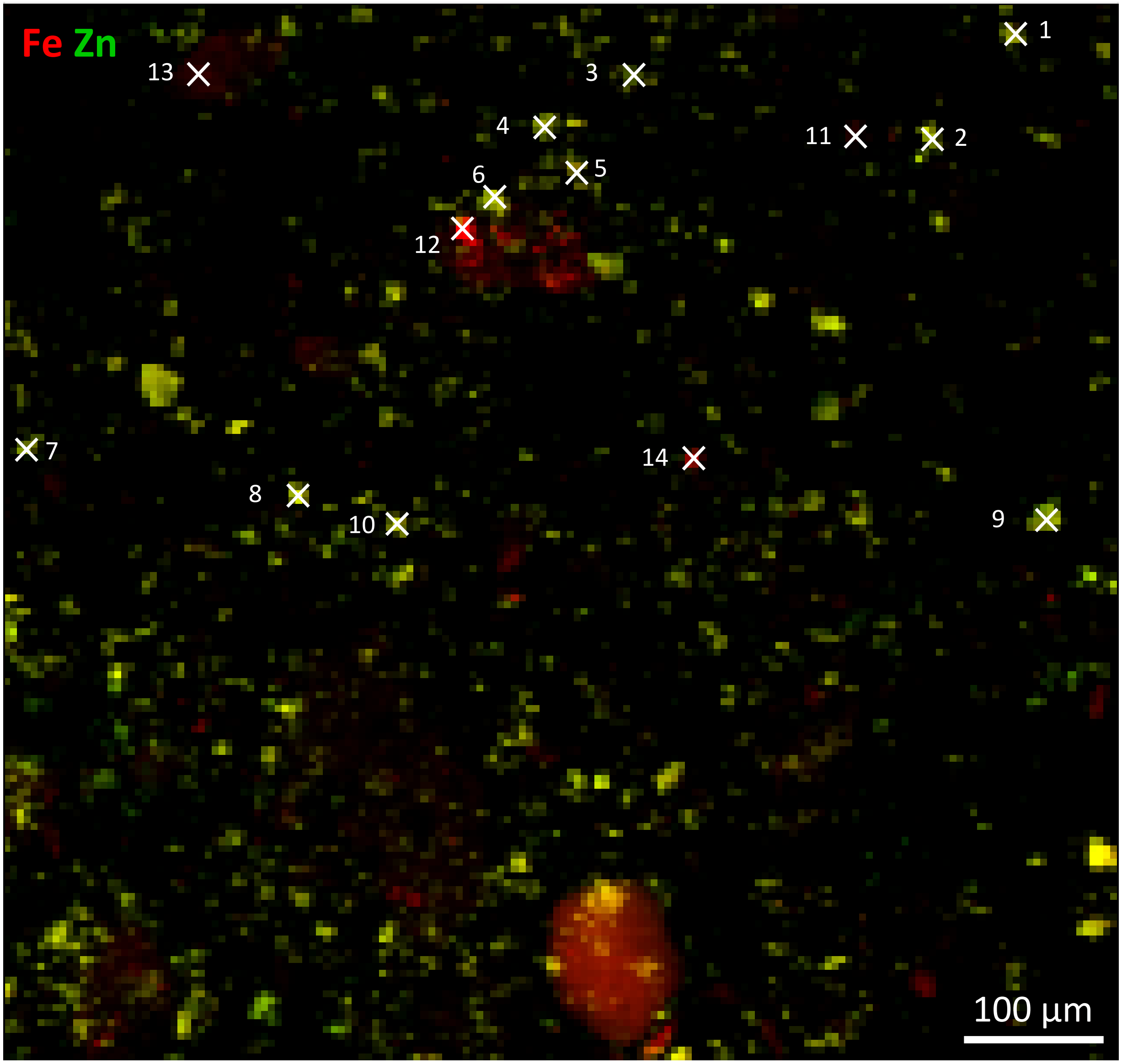

Figure S17 – Micro-XRF mapping of the initial Fh-Zn:sediment mix with 5 wt% Zn and HW sediment (HW 5 ini), showing Fe fluorescence in red and Zn fluorescence in green (yellow color thus corresponding to co-occurring Fe and Zn in Fh-Zn particles). Numbered white crosses show spots where Fe K-edge and Zn K-edge micro-XANES spectra were acquired.

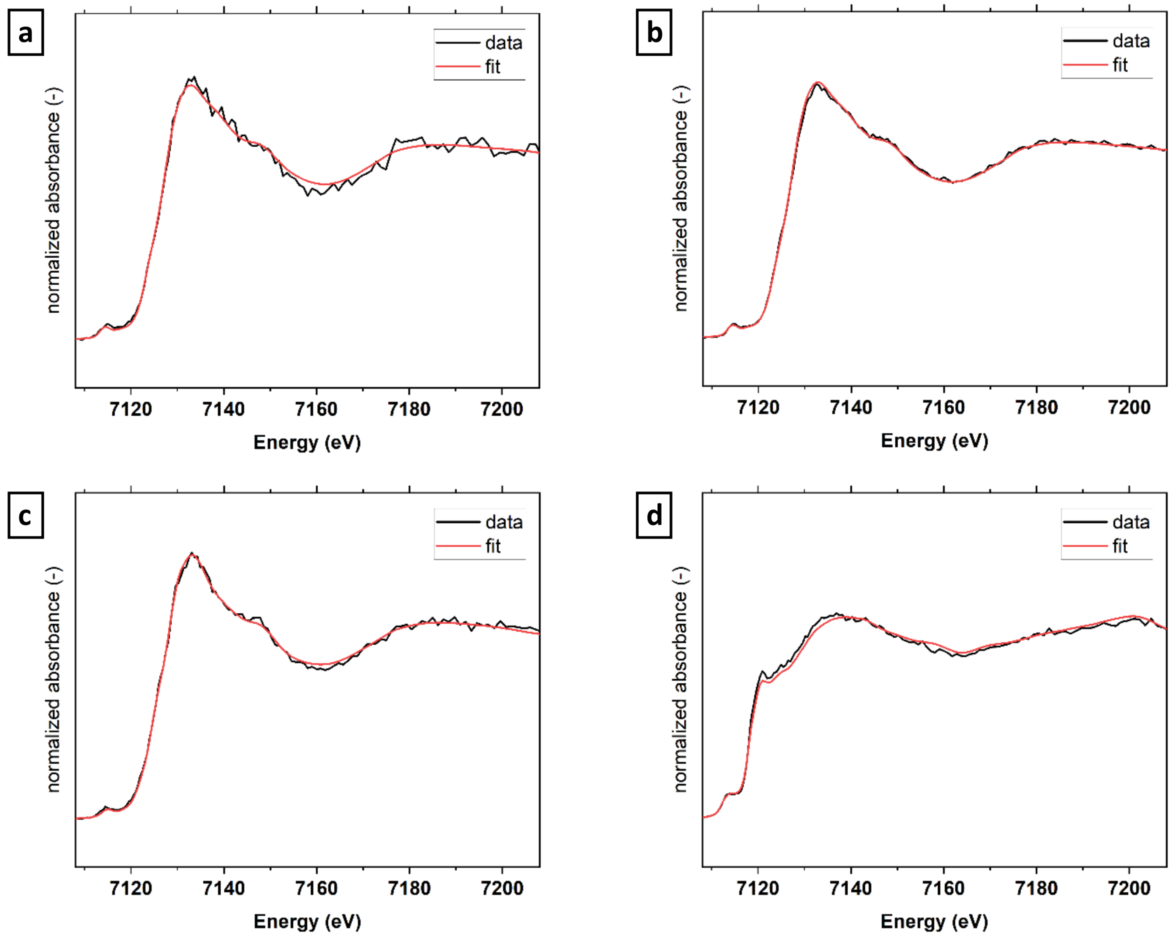

Figure S18 – Examples of Fe K-edge micro-XANES spectra (black) and corresponding LC fits (red) acquired on initial Fh-Zn:sediment mix HW 5 ini: (a) measurement point 3 composed only of Fh-Zn 5 %, (b) measurement point 9 consisting of Fh-Zn only, (c) measurement point 13 fit with 45 % Fh-Zn and 55 % illite, (d) measurement point 12 composed of pyrite (FeS_2).

Table S8 – Fe K-edge micro-XANES LC fitting parameters of initial Fh-Zn:sediment mix HW 5 ini. Rf: R-factor; χ^2_R: reduced chi-square; ΔE_0: shift in E_0 applied to the experimental spectrum to compensate for the absence of energy calibration.

	Component proportions (%)			Fit parameters		
**Point**	**Fh-Zn**	**Pyrite**	**Illite**	**R_f**	**χ^2_R**	**ΔE0 (eV)**
**1**	100	-	-	0.0018	0.00032	-0.277(33)
**2**	100	-	-	0.0013	0.00022	-0.140(27)
**3**	100	-	-	0.0041	0.00077	0.046(50)
**4**	100	-	-	0.0029	0.00055	-0.248(42)
**5**	100	-	-	0.0073	0.00140	-0.528(65)
**6**	100	-	-	0.0015	0.00027	-0.087(29)
**7**	76(2)	24(2)	-	0.0057	0.00099	0.457(71)
**8**	100	-	-	0.0021	0.00040	-0.343(35)
**9**	100	-	-	0.0007	0.00014	0.058(21)
**10**	100	-	-	0.0014	0.00028	-0.254(29)
**11**	100	-	-	0.0044	0.00081	-0.234(50)
**12**	-	100	-	0.0041	0.00043	-0.203(47)
**13**	45(5)	-	55(5)	0.0013	0.00025	-0.475(47)
**14**	100	-	-	0.0066	0.00107	-1.818(58)

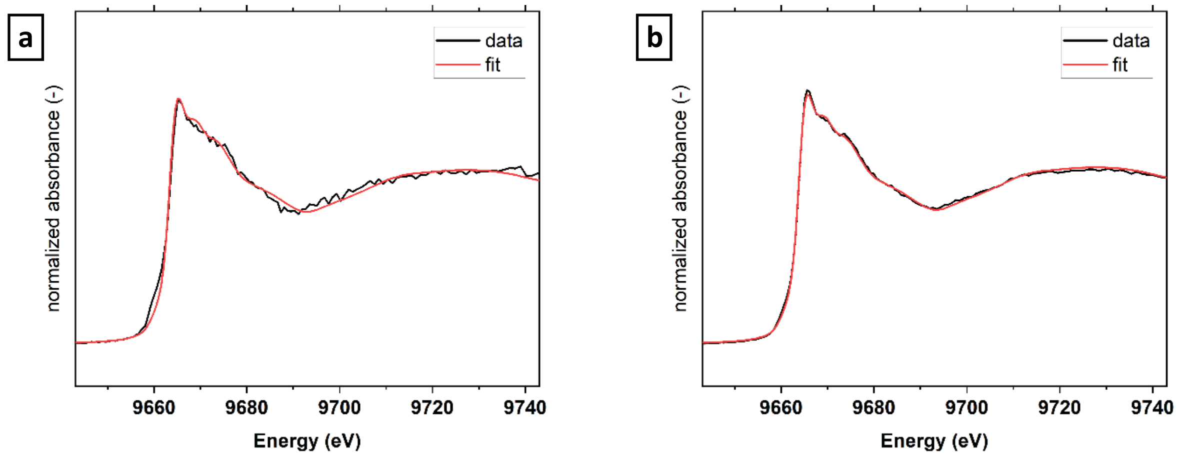

Figure S19 – Examples of Zn K-edge micro-XANES spectra (black) and corresponding LC fits (red) acquired on initial Fh-Zn:sediment mix HW 5 ini: (a) measurement point 3 and (b) measurement point 9, both composed of Fh-Zn 5 % only.

Table S9 – Zn K-edge micro-XANES LC fitting parameters of initial Fh-Zn:sediment mix HW 5 ini. Rf: R-factor; χ^2_R: reduced chi-square; ΔE_0: shift in E_0 applied to the experimental spectrum to compensate for the absence of energy calibration.

	Component (%)	Fit parameters		
**Point**	**Fh-Zn**	**R_f**	**χ^2_R**	**ΔE0 (eV)**
**1**	100	0.0029	0.00063	-0.229
**2**	100	0.0042	0.00093	0.123
**3**	100	0.0048	0.00099	0.107
**4**	100	0.0032	0.00070	0.309
**5**	100	0.0161	0.00331	0.298
**6**	100	0.0006	0.00014	0.526
**8**	100	0.0007	0.00017	0.581
**9**	100	0.0006	0.00013	0.556
**10**	100	0.0006	0.00013	0.580

Note: Zn K-edge micro-XANES spectra of measurement points 7 and 11-14 were not fit because of very low Zn signal.

11. HCl extraction of transformed Fh-Zn: Fe results

Mass balance equations were applied to determine the contributions from the mineral products of Fh-Zn transformation and from the surrounding sediment, in the HCl-extracted solutions. First, a simple isotope mixing equation (Equation S1) was used to calculate the percentage of HCl-extracted Fe originating from transformed Fh-Zn ($\%Fh_{sample}$) as opposed to sedimentary Fe, using measured $f\,^{57}Fe$ isotope ratios ($f\,^{57}Fe_{sample}$) and the endmember ratios from the synthetic ^{57}Fe-labelled Fh-Zn ($f\,^{57}Fe_{Fh\text{-}Zn}$ = 96.14 %) and from the sediment ($f\,^{57}Fe_{sed}$ = 2.12 %, natural abundance). This proportion is shown in Figure S20a.

$$\%Fh_{sample} = \frac{f^{57}Fe_{sample} - f^{57}Fe_{sed}}{f^{57}Fe_{Fh-Zn} - f^{57}Fe_{sed}} \qquad (S1)$$

Then, the proportion of Fe extracted from transformed Fh-Zn (i.e., either the proportion of Fe in poorly crystalline mineral products and/or the proportion of Fe not lost by diffusion) was calculated in Equation S2 as the ratio of the mass of HCl-extracted Fe coming from transformed Fh-Zn to the initial Fe mass in added Fh-Zn. The evolution of these proportions is shown in Figure S20b.

$$\%Fe_{extracted\ from\ Fh-Zn} = \frac{\%Fh_{sample} \times m_{total\ extracted\ Fe}}{m_{initial\ Fe\ in\ Fh-Zn}} \qquad (S2)$$

Eventually, we developed mass balance equations to determine the redox (Fe(II) proportion) of Fe extracted from transformed Fh-Zn ($R_{Fh\text{-}Zn}$), using the measured redox of total HCl-extracted Fe R_{tot} (Figure S20c) and the redox of the sedimentary Fe endmember R_{sed} (Table S6, Figure S12). The final equation is displayed in Equation S3, with results shown in Figure S20d.

$$R_{Fh-Zn} = R_{tot} + \frac{R_{tot} - R_{sed}}{\left({}^{\%Fh_{sample}}\big/_{1 - \%Fh_{sample}} \right)} \qquad (S3)$$

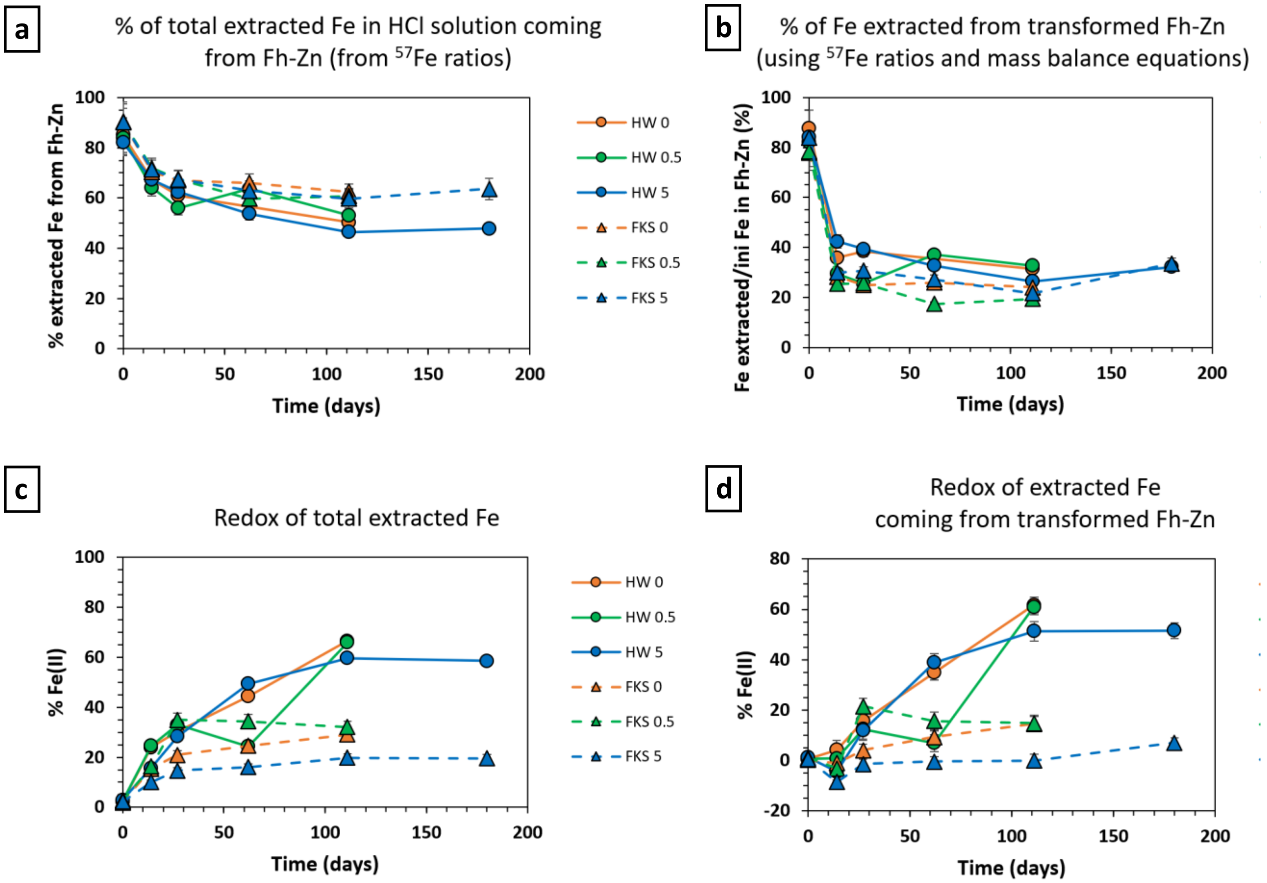

Figure S20 – Fe data from HCl extractions of transformed Fh-Zn:sediment mixes: (a) proportion of total HCl-extracted Fe in solution coming from transformed Fh-Zn (based on f ^{57}Fe measurements); (b) Fe proportion from Fh-Zn transformation products compared to initial Fe in Fh-Zn, after removing the contribution from sedimentary Fe; if all Fh-Zn transformation products are dissolved by 0.5 M HCl, this proportion corresponds to the remaining Fe fraction after diffusive ^{57}Fe losses out of the mesh bag. (c) Fe(II) proportions of total extracted Fe, and (d) Fe(II) proportions of extracted Fe coming from transformed Fh-Zn, after removing the contribution from sedimentary Fe. Note that the vertical scale on (d) is shifted by 20 % to account for negative values (for which the mass balance was not ideal).

The oxidation state of HCl-extracted Fe coming from Fh-Zn (Figure S20d) is similar to the redox of solid-state ^{57}Fe analyzed by Mössbauer (Figure 2c). This is more evidence to show that the Fh-Zn transformation products were completely dissolved by 0.5 M HCl.

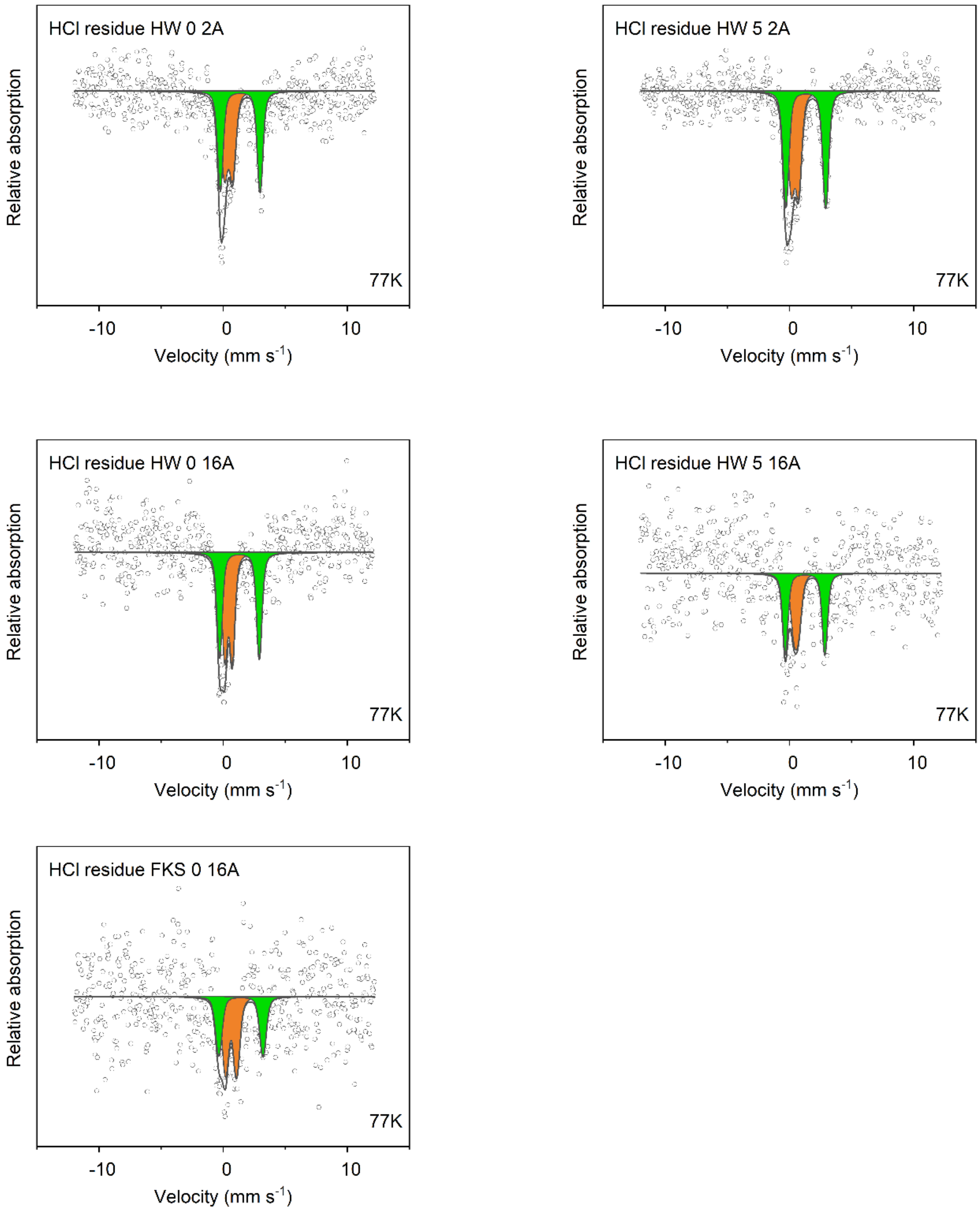

Figure S21 – Mössbauer spectra at 77 K of solid residues after HCl extractions of a selection of transformed Fh-Zn:sediment mixes in the HW and FKS mesocosms, with Fh-Zn 0 % (left) or 5 % (right), reacted for 2 weeks (top) or 16 weeks (bottom). The experimental data is shown as open circles, the calculated fit as a black line, and the fitted components as filled areas: green for the Fe(II) doublet, dark red for the Fe(III) doublet. These spectra were collected over longer periods than most of the corresponding Fh-Zn:sediment mixes and show a comparatively much lower signal. The fitting parameters of Fe(II) and Fe(III) doublets are similar to that of bulk sediment samples (Figure S8 and Table S4).

12. Mössbauer of transformed Fh-Zn:sediment mixes

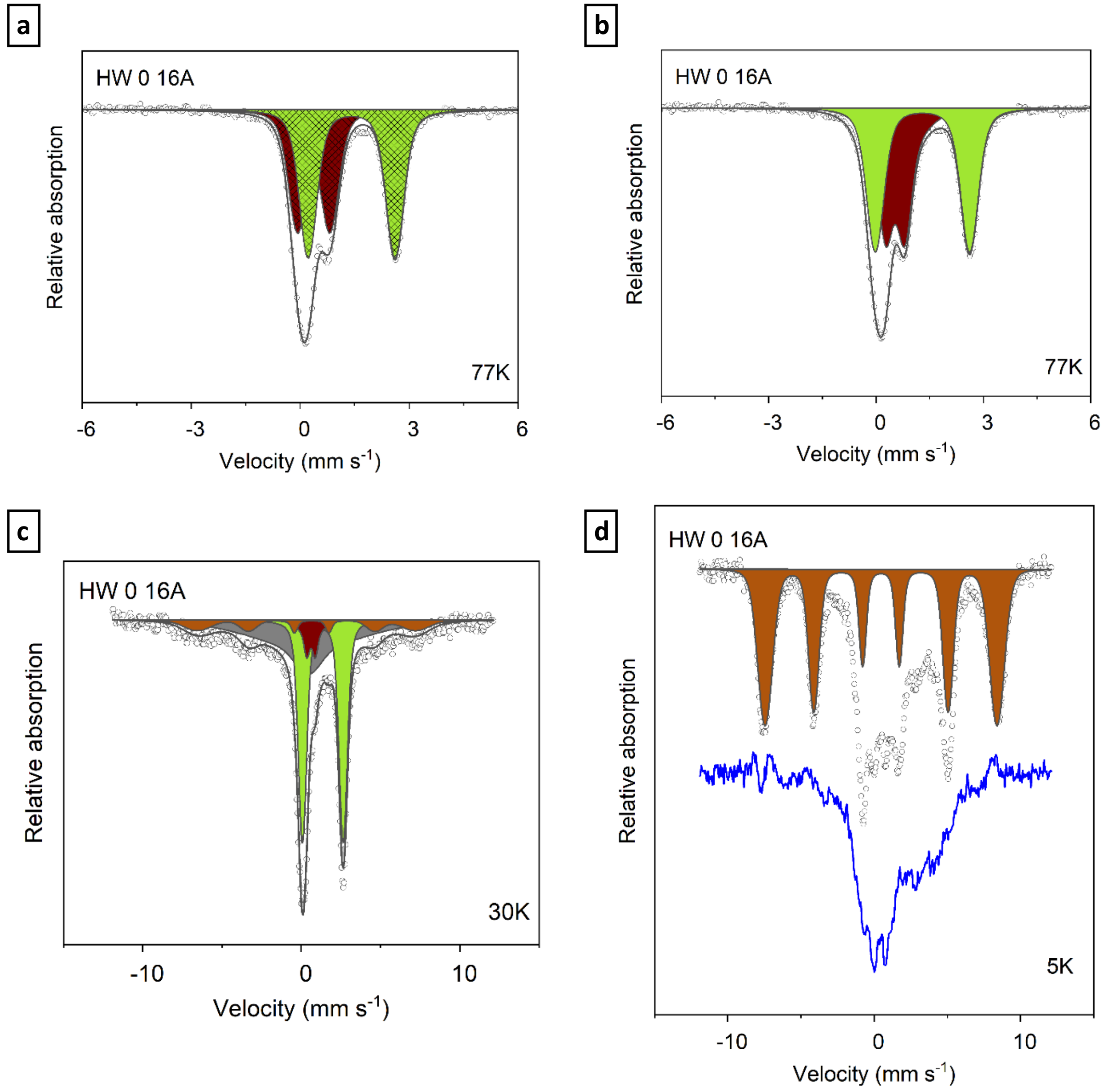

Figure S22 – Examples of Mössbauer spectra at various temperatures and fitting solutions for sample HW 0 16A. The experimental data is shown as open circles and the calculated fit as a black line. (a and b) Same experimental spectrum acquired at 77 K with two possible fit solutions, where the Fe(III) doublet in dark red either overlaps the left peak of the Fe(II) doublet in green (a) or is entirely overlapped by the Fe(II) doublet (b). (c) 30 K spectrum of the same sample; the four main components are shown as filled areas: green for the Fe(II) doublet, dark red for the Fe(III) doublet, gray for the collapsed feature and brown for the Fe(III) sextet. At 30 K, the Fe(III) component starts ordering, leaving a clear Fe(II) doublet which fit parameters are better constrained than at 77 K; the fitting solution of (b) is subsequently favored over (a). (d) 5 K spectrum where only an Fe(III) sextet is fit in brown, leaving the residue – corresponding to a poorly ordered Fe(II) octet – shown as a blue line.

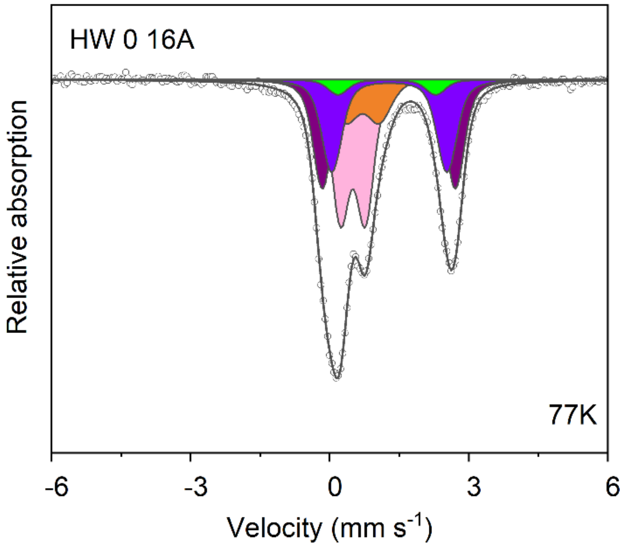

Figure S23 – Example of a complex fit solution for the Mössbauer spectrum of sample HW 0 16A. Instead of two doublets describing Fe(II) and Fe(III) components, a combination of five doublets could provide a satisfying fit. These doublets could correspond approximately to ferrihydrite (Fe(III), orange), siderite (Fe(II), green) and green rust (three doublets: 2 Fe(II) in purple and violet and 1 Fe(III) in pink). However, such fits are unstable (several parameters had to be fixed) and do not have a unique solution (several proportions and parameter values were possible). We therefore kept the simple "2 doublets" solutions. Both solutions provide similar total Fe(II)-Fe(III) proportions.

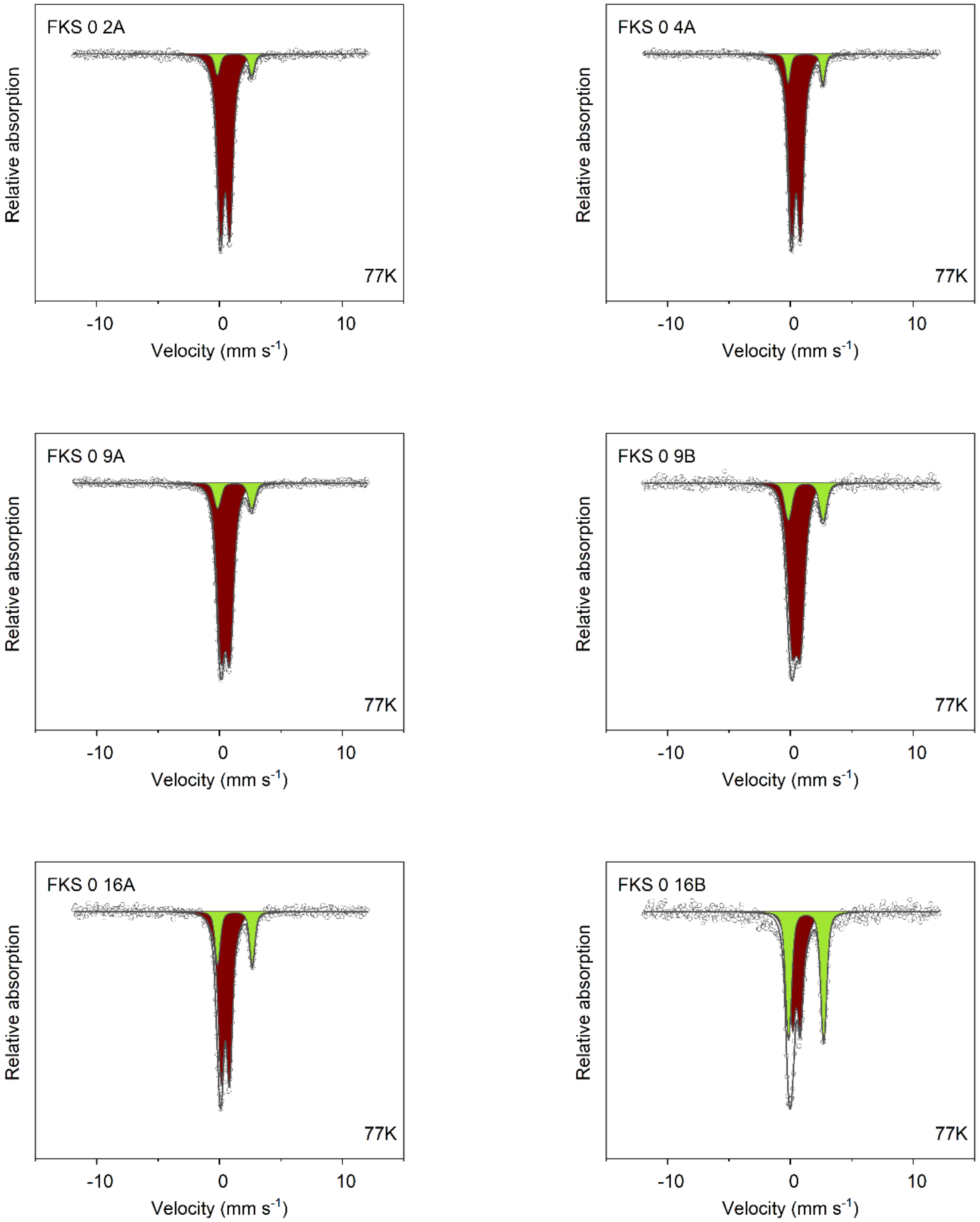

Figure S24– Mössbauer spectra at 77 K of transformed Fh-Zn samples initially containing 0 wt% Zn, reacted in sediments from Friedrichskoog (FKS 0) during two (2A), four (4A), nine (9A and 9B) and sixteen weeks (16A and 16B). The experimental data is shown as open circles, the calculated fit as a black line, and the two main components as filled areas: green for the Fe(II) doublet, dark red for the Fe(III) doublet (Table S11).

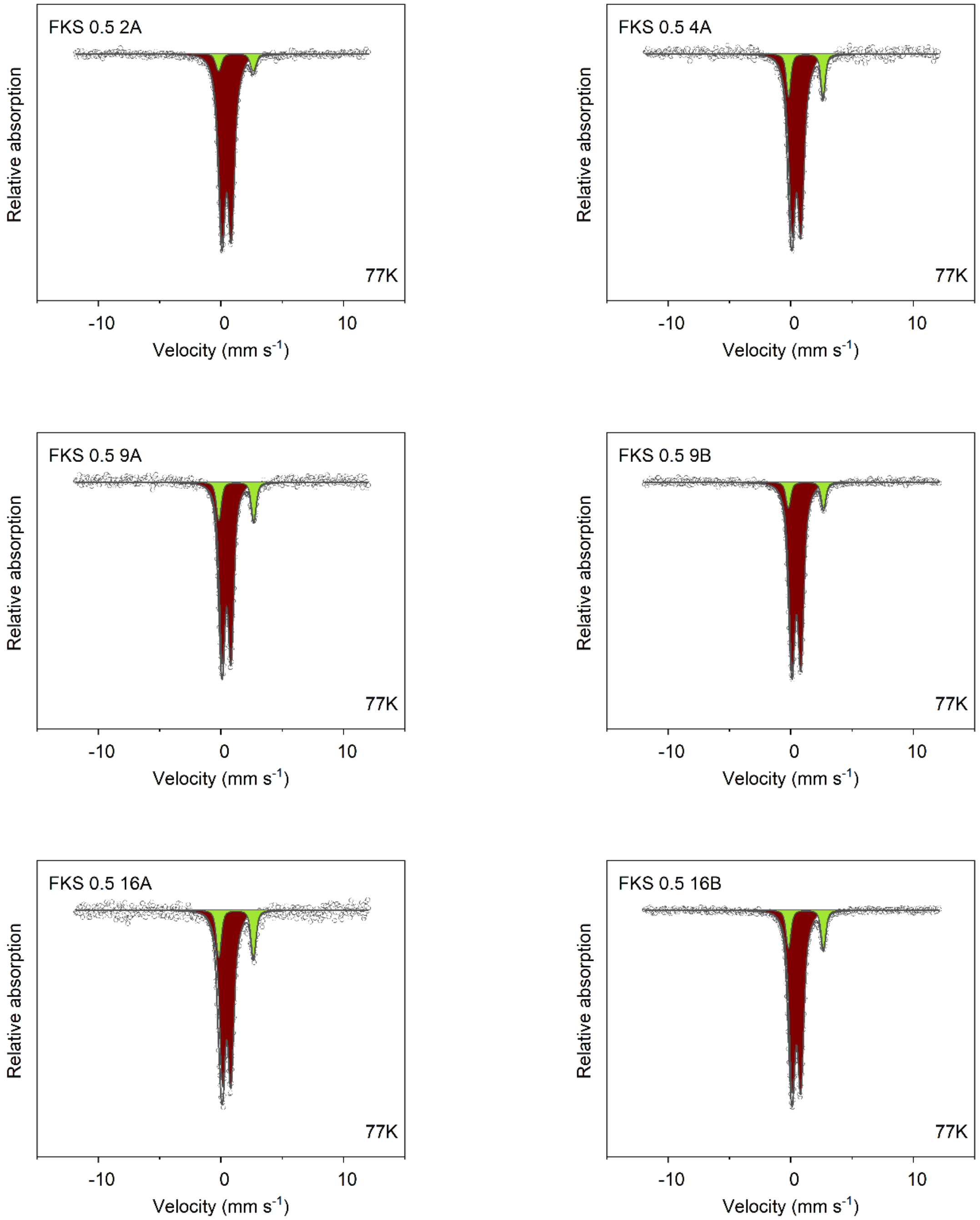

Figure S25 – Mössbauer spectra at 77 K of transformed Fh-Zn samples initially containing 0.5 wt% Zn, reacted in sediments from Friedrichskoog (FKS 0.5) during two (2A), four (4A), nine (9A and 9B) and sixteen weeks (16A and 16B). The experimental data is shown as open circles, the calculated fit as a black line, and the two main components as filled areas: green for the Fe(II) doublet, dark red for the Fe(III) doublet (Table S11).

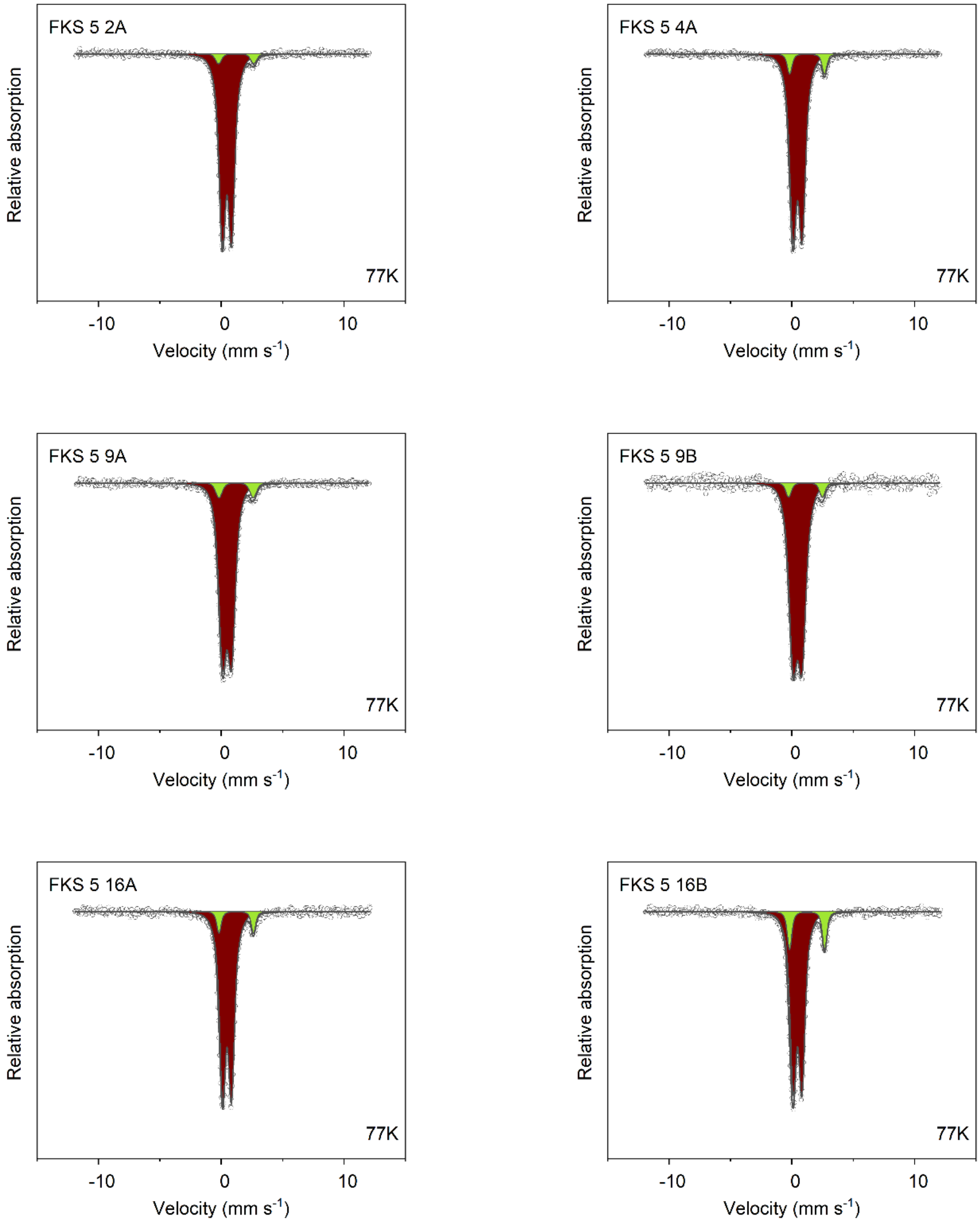

Figure S26 – Mössbauer spectra at 77 K of transformed Fh-Zn samples initially containing 5 wt% Zn, reacted in sediments from Friedrichskoog (FKS 5) during two (2A), four (4A), nine (9A and 9B) and sixteen weeks (16A and 16B). The experimental data is shown as open circles, the calculated fit as a black line, and the two main components as filled areas: green for the Fe(II) doublet, dark red for the Fe(III) doublet (Table S11).

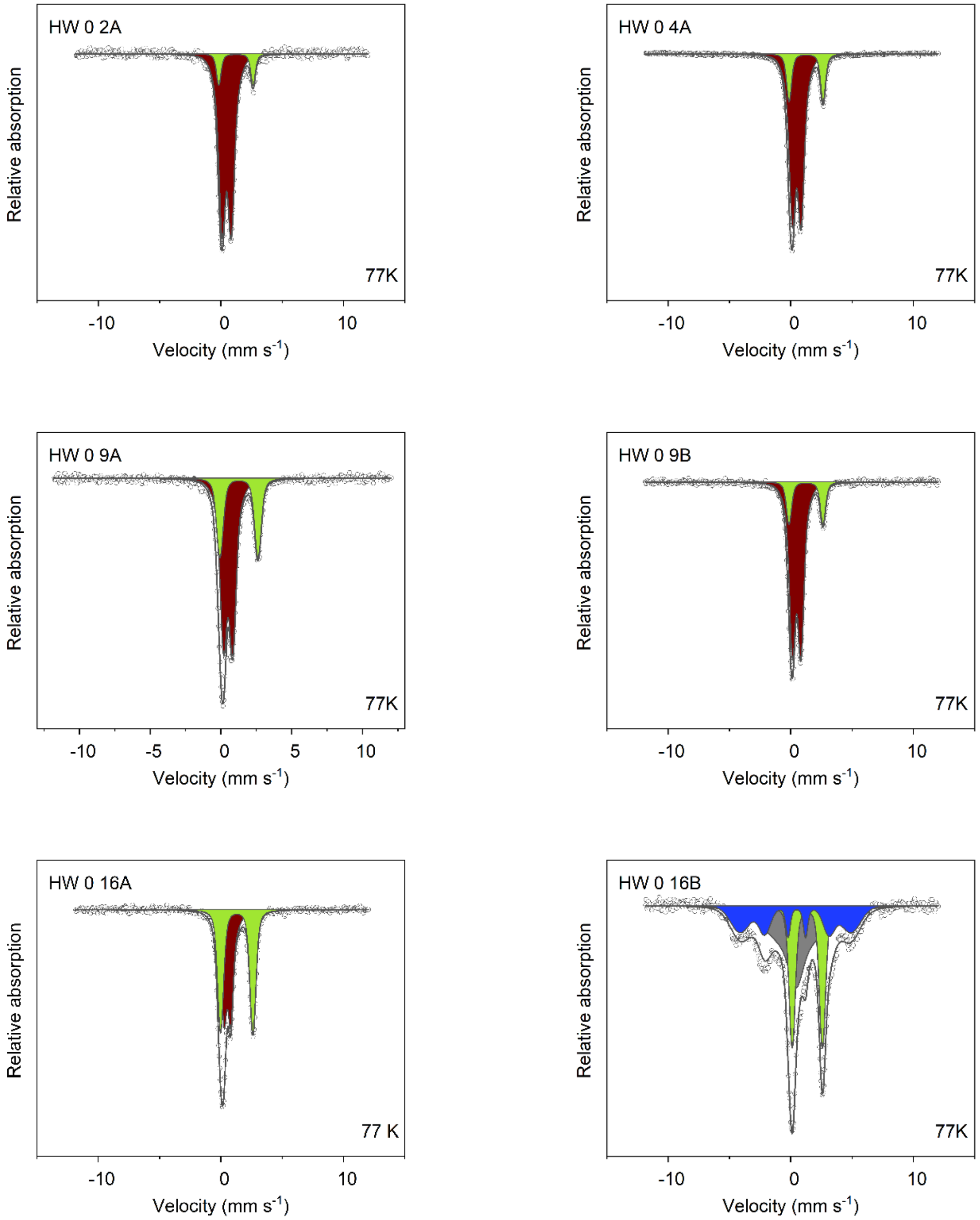

Figure S27 – Mössbauer spectra at 77 K of transformed Fh-Zn samples initially containing 0 wt% Zn, reacted in sediments from Hollerwettern (HW 0) during two (2A), four (4A), nine (9A and 9B) and sixteen weeks (16A and 16B). The experimental data is shown as open circles, the calculated fit as a black line, and the two main components as filled areas: green for the Fe(II) doublet, dark red for the Fe(III) doublet (Table S12). The exception is for sample HW 0 16B that shows a different Fe speciation (Table S10): in addition to the green Fe(II) doublet, we observe a sextet (blue) and a disordered Fe(III) phase in the form of a collapsed feature (grey).

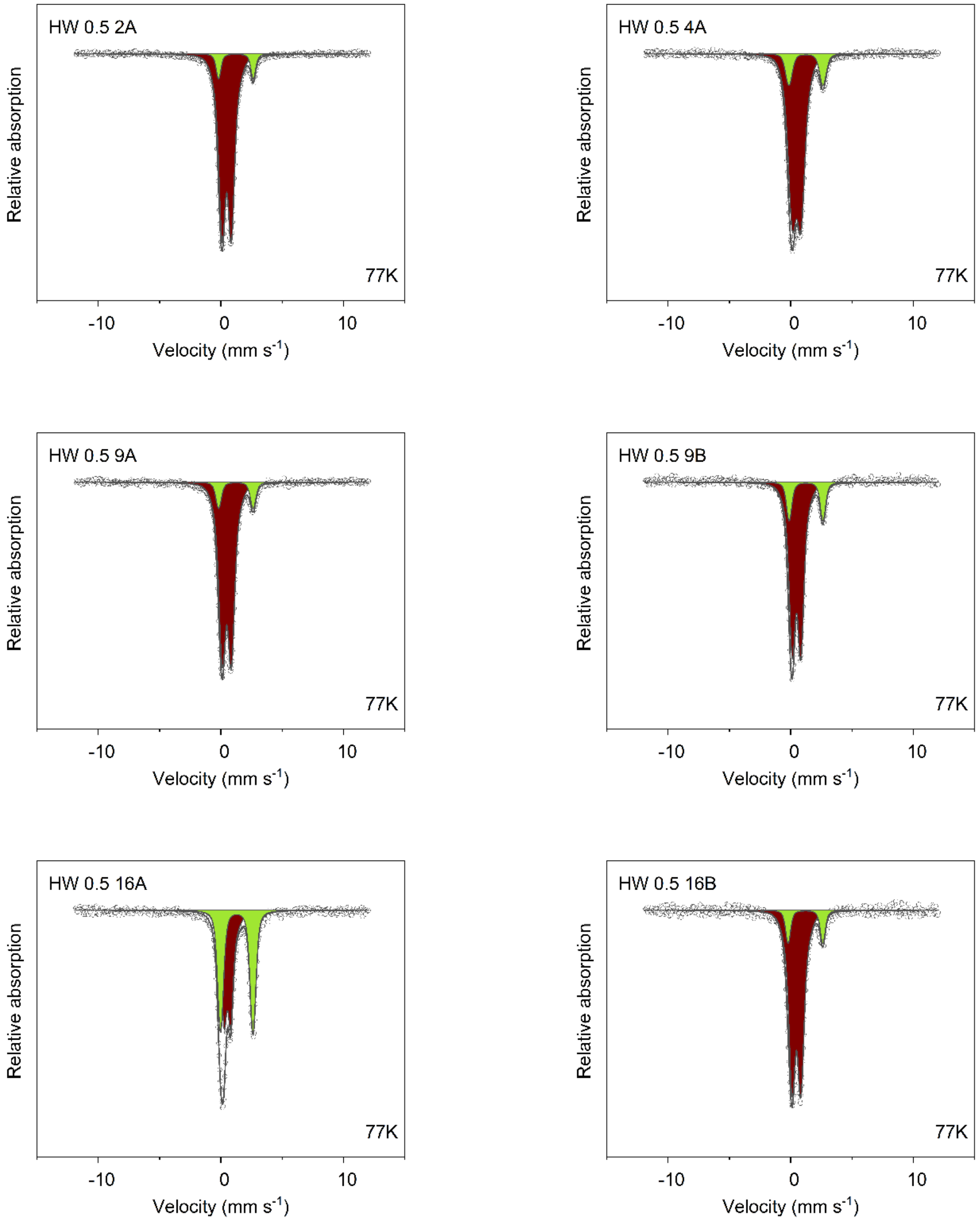

Figure S28 – Mössbauer spectra at 77 K of transformed Fh-Zn samples initially containing 0.5 wt% Zn, reacted in sediments from Hollerwettern (HW 0.5) during two (2A), four (4A), nine (9A and 9B) and sixteen weeks (16A and 16B). The experimental data is shown as open circles, the calculated fit as a black line, and the two main components as filled areas: green for the Fe(II) doublet, dark red for the Fe(III) doublet (Table S12).

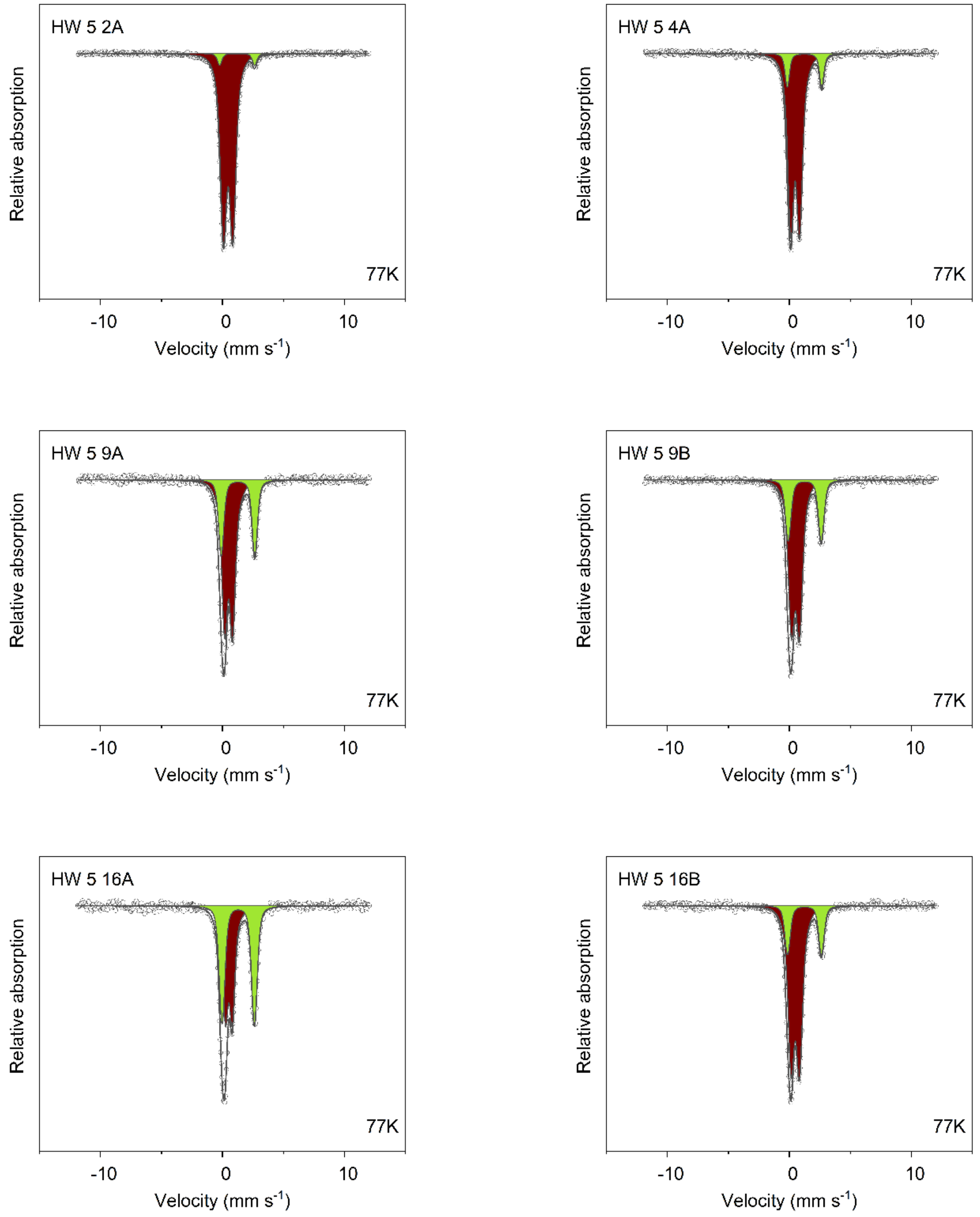

Figure S29 – Mössbauer spectra at 77 K of transformed Fh-Zn samples initially containing 5 wt% Zn, reacted in sediments from Hollerwettern (HW 5) during two (2A), four (4A), nine (9A and 9B) and sixteen weeks (16A and 16B). The experimental data is shown as open circles, the calculated fit as a black line, and the two main components as filled areas: green for the Fe(II) doublet, dark red for the Fe(III) doublet (Table S12).

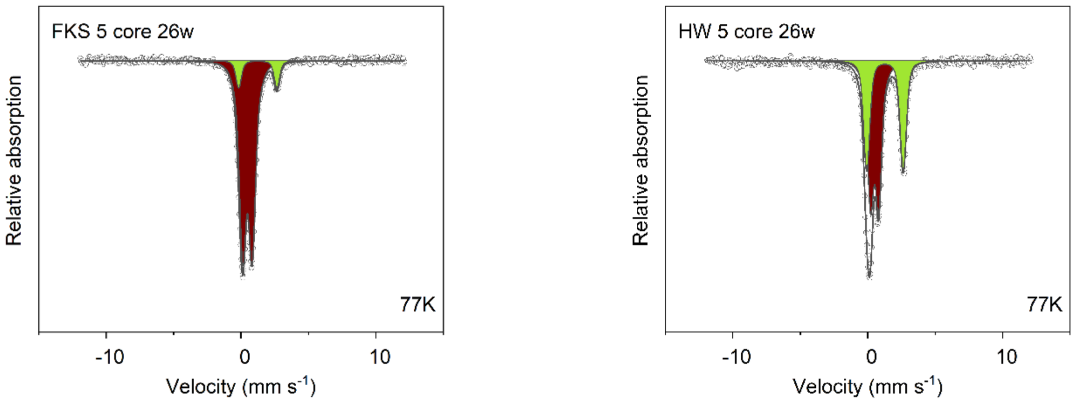

Figure S30 – Mössbauer spectra at 77 K of transformed Fh-Zn samples initially containing 5 wt% Zn, reacted in sediments from Friedrichskoog (FKS 5) or Hollerwettern (HW 5) for 26 weeks and sampled within sediment cores. The experimental data is shown as open circles, the calculated fit as a black line, and the two main components as filled areas: green for the Fe(II) doublet, dark red for the Fe(III) doublet (Tables S11 and S12).

Table S10 – Fitting parameters (xVBF model) of Mössbauer spectra acquired at 77 K for transformed Fh-Zn:sediment sample HW 0 16B from the HW mesocosm. CS: center/isomer shift, QS: quadrupole splitting/shift, σ_{QS}: standard deviation of QS, H: Hyperfine field, σ_H: standard deviation of H. Uncertainties on the last digits, calculated from the covariance matrix, are given in brackets (SD of the fit parameter).

**Sample**	**Time step Fh-Zn**	**Component**	**Contribution (%)**	**CS (mm/s)**	**QS (mm/s)**	**σ_{QS} (mm/s)**	**H (T)**	**σ_H (T)**
**HW 0 16B**	Week 16 Fh-Zn 0 %	Fe(II) doublet	29(2)	1.353(6)	2.47(1)	0.39(2)	-	-
		Sextet	29(3)	0.45(3)	-0.05(3)	-	28.0(3)	4.6(4)
		CF	43(3)	0.42(4)	-	-	-	19(2)

Table S11 – Fitting parameters (xVBF model) of Mössbauer spectra acquired at 77 K for transformed Fh-Zn:sediment mixes from the FKS mesocosm. CS: center/isomer shift, QS: quadrupole splitting, σ_{QS}: standard deviation of QS. Uncertainties on the last digits are given in brackets; these correspond either to the SD on the average of 2 replicate measurements (A, B) or the SD of the fit parameter (calculated from the covariance matrix) if only one measurement.

Sample (replicates)	Time step Fh-Zn	Doublet	Contribution (%)	CS (mm/s)	QS (mm/s)	σ_{QS} (mm/s)
**FKS 0 2 (A)**	Week 2	Fe(II)	9.1(6)	1.23(2)	2.80(4)	0.31(6)
	Fh-Zn 0 %	Fe(III)	90.9(6)	0.468(4)	0.737(7)	0.37(1)
**FKS 0 4 (A)**	Week 4	Fe(II)	10.9(5)	1.24(1)	2.84(2)	0.24(4)
	Fh-Zn 0 %	Fe(III)	89.1(5)	0.466(3)	0.725(6)	0.36(1)
**FKS 0 9 (A, B)**	Week 9	Fe(II)	15(4)	1.24(1)	2.80(3)	0.46(3)
	Fh-Zn 0 %	Fe(III)	85(4)	0.478(3)	0.7(2)	0.49(3)
**FKS 0 16 (A, B)**	Week 16	Fe(II)	36(20)	1.27(2)	2.84(3)	0.32(4)
	Fh-Zn 0 %	Fe(III)	64(20)	0.48(2)	0.64(4)	0.31(3)
**FKS 0.5 2 (A)**	Week 2	Fe(II)	7.7(6)	1.22(2)	2.83(5)	0.34(6)
	Fh-Zn 0.5 %	Fe(III)	92.3(6)	0.466(3)	0.746(6)	0.36(1)
**FKS 0.5 4 (A)**	Week 4	Fe(II)	15.0(9)	1.23(2)	2.81(3)	0.22(4)
	Fh-Zn 0.5 %	Fe(III)	85.0(9)	0.473(6)	0.71(1)	0.35(2)
**FKS 0.5 9 (A, B)**	Week 9	Fe(II)	13(4)	1.25(1)	2.86(1)	0.256(8)
	Fh-Zn 0.5 %	Fe(III)	87(4)	0.4669(3)	0.711(8)	0.338(7)
**FKS 0.5 16 (A, B)**	Week 16	Fe(II)	17(3)	1.240(2)	2.84(1)	0.264(7)
	Fh-Zn 0.5 %	Fe(III)	83(3)	0.4701(5)	0.693(6)	0.34(1)
**FKS 5 2 (A)**	Week 2	Fe(II)	4.8(6)	1.21(4)	2.87(8)	0.36(10)
	Fh-Zn 5 %	Fe(III)	95.2(6)	0.466(3)	0.736(5)	0.361(9)
**FKS 5 4 (A)**	Week 4	Fe(II)	7.8(5)	1.24(2)	2.83(3)	0.26(5)
	Fh-Zn 5 %	Fe(III)	92.2(5)	0.468(3)	0.727(5)	0.379(9)
**FKS 5 9 (A, B)**	Week 9	Fe(II)	6(1)	1.16(7)	2.77(3)	0.37(7)
	Fh-Zn 5 %	Fe(III)	94(1)	0.47(1)	0.707(9)	0.448(5)
**FKS 5 16 (A, B)**	Week 16	Fe(II)	11(4)	1.23(1)	2.83(3)	0.23(1)
	Fh-Zn 5 %	Fe(III)	89(4)	0.471(4)	0.706(7)	0.342(6)
**FKS 5 core 26w**	Week 26	Fe(II)	10.6(6)	1.24(2)	2.84(3)	0.28(4)
	Fh-Zn 5 %	Fe(III)	89.4(6)	0.473(3)	0.693(5)	0.350(9)

Table S12 – Fitting parameters (xVBF model) of Mössbauer spectra acquired at 77 K for transformed Fh-Zn:sediment mixes from the HW mesocosm. CS: center/isomer shift, QS: quadrupole splitting, σ_{QS}: standard deviation of QS. Uncertainties on the last digits are given in brackets; these correspond either to the SD on the average of 2 replicate measurements (A, B) or the SD of the fit parameter (calculated from the covariance matrix) if only one measurement.

Sample (replicates)	Time step Fh-Zn	Doublet	Contribution (%)	CS (mm/s)	QS (mm/s)	σ_{QS} (mm/s)
**HW 0 2 (A)**	Week 2	Fe(II)	11.6(8)	1.22(2)	2.80(4)	0.24(5)
	Fh-Zn 0 %	Fe(III)	88.4(8)	0.469(5)	0.74(1)	0.36(2)
**HW 0 4 (A)**	Week 4	Fe(II)	18.9(2)	1.251(4)	2.771(8)	0.28(1)
	Fh-Zn 0 %	Fe(III)	81.1(2)	0.489(2)	0.703(3)	0.363(5)
**HW 0 9 (A, B)**	Week 9	Fe(II)	25(9)	1.26(2)	2.73(6)	0.32(3)
	Fh-Zn 0 %	Fe(III)	75(9)	0.50(2)	0.67(3)	0.341(6)
**HW 0 16 (A x2)**	Week 16	Fe(II)	53(1)	1.298(1)	2.630(9)	0.358(6)
	Fh-Zn 0 %	Fe(III)	47(1)	0.531(7)	0.54(1)	0.272(1)
**HW 0.5 2 (A)**	Week 2	Fe(II)	9.5(4)	1.23(1)	2.83(2)	0.26(3)
	Fh-Zn 0.5 %	Fe(III)	90.5(4)	0.477(2)	0.743(4)	0.355(7)
**HW 0.5 4 (A)**	Week 4	Fe(II)	14.4(6)	1.24(2)	2.78(3)	0.43(4)
	Fh-Zn 0.5 %	Fe(III)	85.6(6)	0.482(5)	0.70(1)	0.46(2)
**HW 0.5 9 (A, B)**	Week 9	Fe(II)	14(4)	1.24(2)	2.78(4)	0.32(1)
	Fh-Zn 0.5 %	Fe(III)	86(4)	0.481(2)	0.71(3)	0.36(2)
**HW 0.5 16 (A, B)**	Week 16	Fe(II)	33(28)	1.25(7)	2.74(13)	0.32(5)
	Fh-Zn 0.5 %	Fe(III)	67(28)	0.50(4)	0.62(13)	0.31(6)
**HW 5 2 (A)**	Week 2	Fe(II)	4.6(4)	1.20(2)	2.87(4)	0.23(5)
	Fh-Zn 5 %	Fe(III)	95.4(4)	0.467(2)	0.755(3)	0.363(5)
**HW 5 4 (A)**	Week 4	Fe(II)	12.9(4)	1.241(8)	2.82(2)	0.22(2)
	Fh-Zn 5 %	Fe(III)	87.1(4)	0.477(2)	0.715(4)	0.333(7)
**HW 5 9 (A, B)**	Week 9	Fe(II)	29(3)	1.27(1)	2.73(3)	0.32(2)
	Fh-Zn 5 %	Fe(III)	71(3)	0.501(6)	0.65(1)	0.34(4)
**HW 5 16 (A, B)**	Week 16	Fe(II)	37(22)	1.27(4)	2.71(9)	0.33(2)
	Fh-Zn 5 %	Fe(III)	63(22)	0.51(3)	0.61(9)	0.32(6)
**HW 5 core 26w**	Week 26	Fe(II)	43.2(6)	1.279(6)	2.71(1)	0.34(1)
	Fh-Zn 5 %	Fe(III)	56.8(6)	0.510(5)	0.59(1)	0.31(2)

Table S13 – Mössbauer fitting parameters at 77 K of initial Fh-Zn samples (containing 0, 0.5 and 5 wt% Zn) and average parameters obtained for the Fe(II) and Fe(III) doublets of transformed Fh-Zn samples. CS: Center shift/Isomer shift, QS: quadrupole shift, σ_{QS}: standard deviation of the QS.

Sample	Doublet	CS (mm/s)	QS (mm/s)	σ_{QS} (mm/s)
**Initial Fh-Zn 0 %**	Fe(III)	0.456(4)	1.253(8)	1.02(2)
**Initial Fh-Zn 0.5 %**	Fe(III)	0.456(4)	1.280(8)	1.04(2)
**Initial Fh-Zn 5 %**	Fe(III)	0.457(1)	1.015(1)	0.803(4)
**All transformed Fh-Zn**	Fe(II)	1.25(8)	2.78(17)	0.31(13)
	Fe(III)	0.48(4)	0.71(18)	0.37(12)
- **All from HW**	Fe(II)	1.26(7)	2.73(19)	0.31(11)
	Fe(III)	0.49(5)	0.69(20)	0.35(12)
- **All from FKS**	Fe(II)	1.23(7)	2.83(6)	0.30(15)
	Fe(III)	0.47(2)	0.73(13)	0.38(11)
**HW 2 weeks**	Fe(II)	1.22(2)	2.83(6)	0.24(2)
	Fe(III)	0.47(1)	0.74(1)	0.36(1)
**HW 16 weeks**	Fe(II)	1.27(7)	2.69(16)	0.33(6)
	Fe(III)	0.51(5)	0.59(15)	0.30(7)

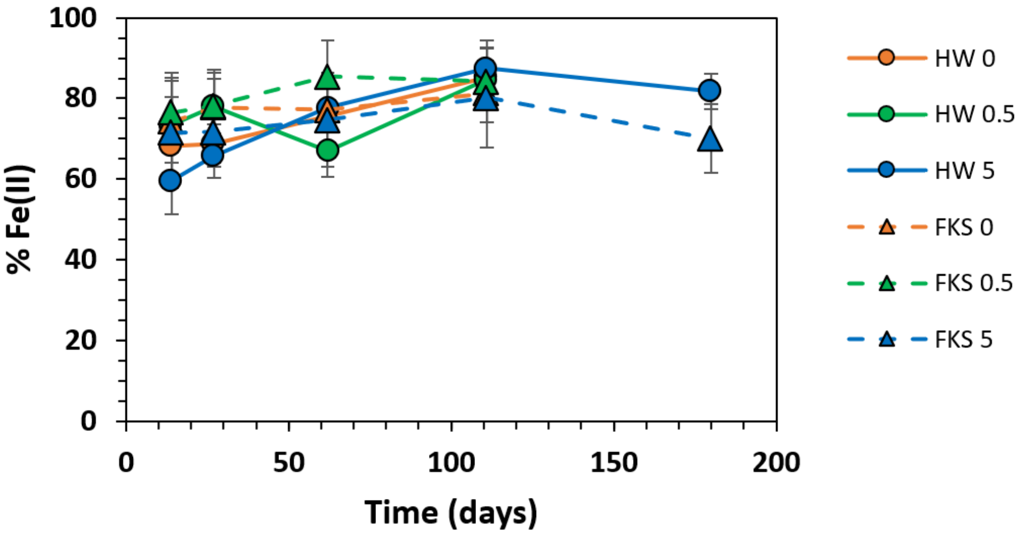

Figure S31 – Total Fe(II) proportions of all transformed Fh-Zn, corresponding to the sum of remaining solid-phase $^{57}Fe(II)$ (analyzed by Mössbauer) shown in Figure 2c and Fe(II) lost by diffusion outside of the sample mesh bag, using proportions determined by Fe isotope measurements (Figure S20b). For simplicity, we hypothesized that all lost Fe was reduced Fe(II).

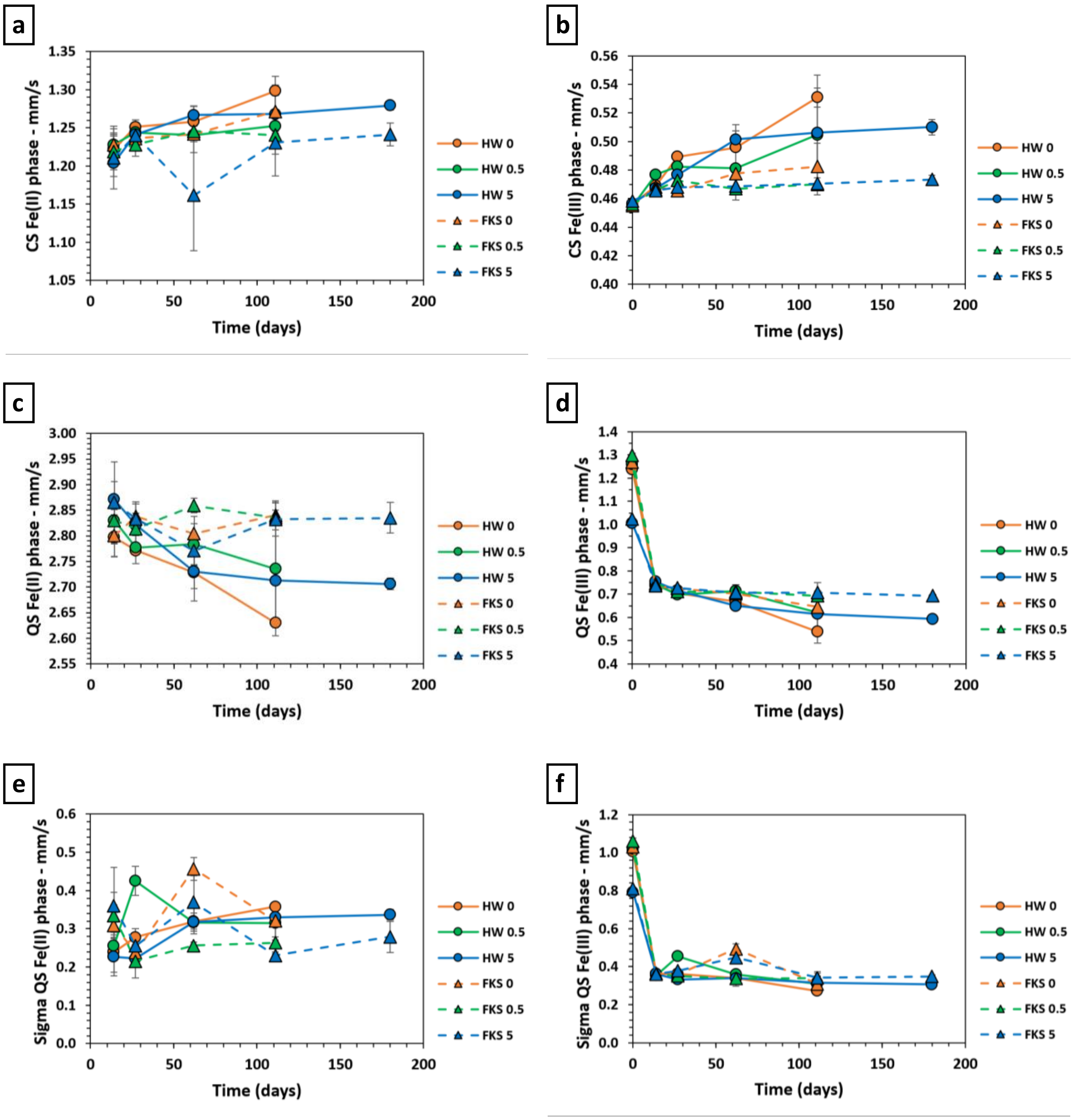

Figure S32 – Evolution of fit parameters of the Mössbauer spectra of Fh-Zn:sediment mixes over the mineral transformation of Fh-Zn, for each of the HW (circles, solid line) and FKS (triangles, dashed line) mesocosms and each of the Fh-Zn 0 % (orange), 0.5 % (green) and 5 % (blue). (a) Center shift (CS) of the Fe(II) doublet; (b) CS of the Fe(III) doublet; (c) Quadrupole splitting (QS) of the Fe(II) doublet; (d) QS of the Fe(III) doublet; (e) standard deviation of the QS (σ_{QS}) of the Fe(II) doublet; (f) σ_{QS} of the Fe(III) doublet.

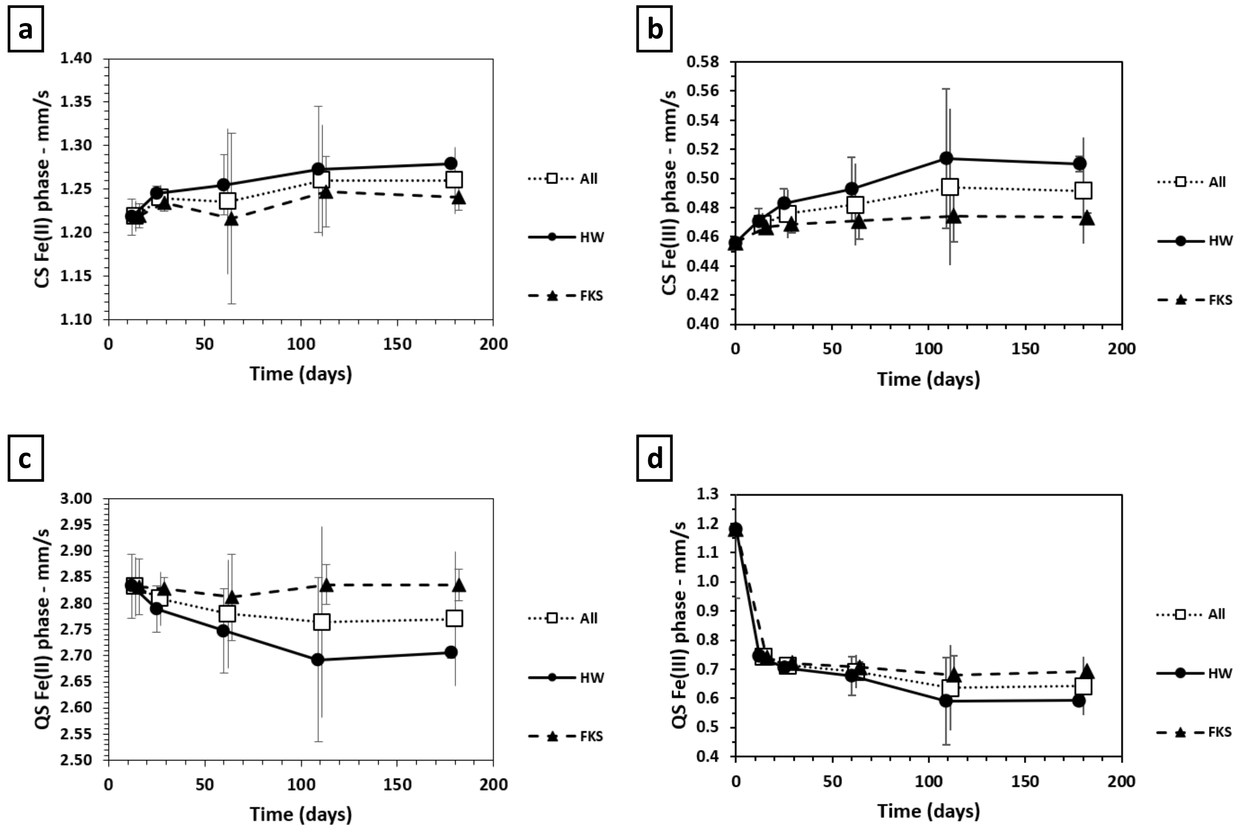

Figure S33 – Evolution of the average fit parameters of Mössbauer spectra of Fh-Zn:sediment mixes over the mineral transformation of Fh-Zn, for HW (black circles) and FKS (black triangles) mesocosms and all samples (open squares). (a) Center shift (CS) of the Fe(II) doublet; (b) CS of the Fe(III) doublet; (c) Quadrupole splitting (QS) of the Fe(II) doublet; (d) QS of the Fe(III) doublet. The x-axis values were slightly shifted for visual purposes.

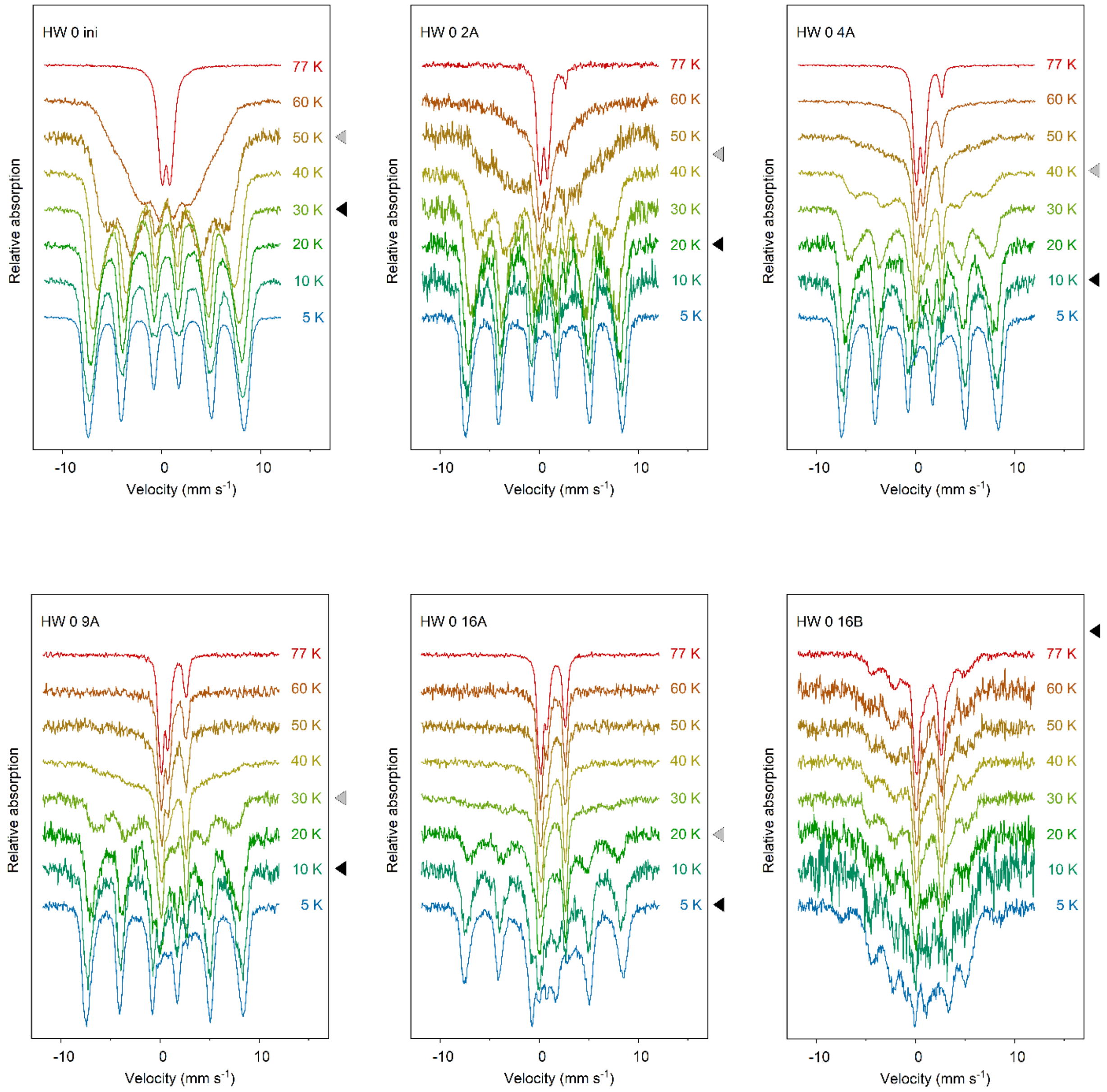

Figure S34 – Temperature profiles of Mössbauer spectra of transformed Fh-Zn samples initially containing 0 wt% Zn, reacted in sediments from Hollerwettern (HW 0) for 0 (ini), two (2A), four (4A), nine (9A) and sixteen weeks (16A and 16B). Each temperature of acquisition is represented by a given color, grading from red (77 K, all reacted samples) to blue (5 K). Arrows on the right-hand side indicate the temperature at which a sextet appears (gray arrow) and is fully ordered (black arrow).

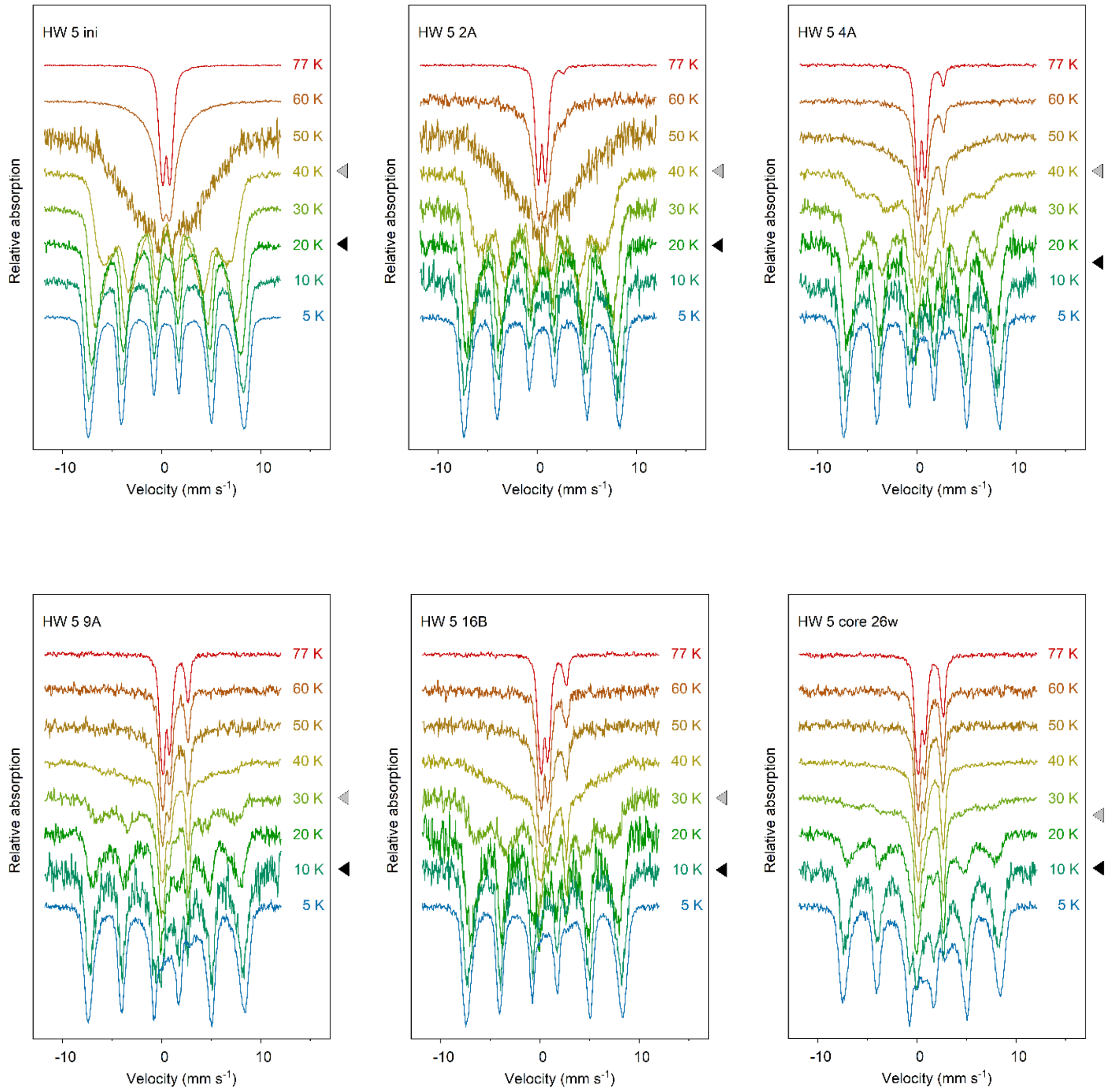

Figure S35 – Temperature profiles from 77 to 5 K of Mössbauer spectra of transformed Fh-Zn samples initially containing 5 wt% Zn, reacted in sediments from Hollerwettern (HW 5) for 0 (ini), two (2A), four (4A), nine (9A), sixteen (16B) and twenty-six weeks (core 26w). Each temperature of acquisition is represented by a given color, grading from red (77 K) to blue (5 K). Arrows on the right-hand side indicate the temperature at which a sextet appears (gray arrow) and is fully ordered (black arrow).

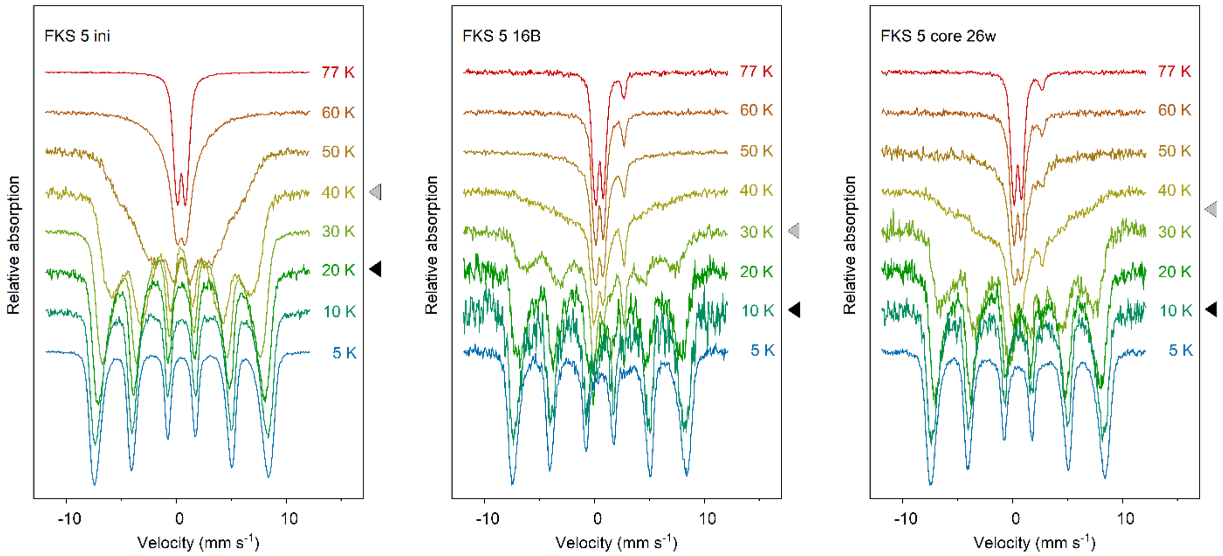

Figure S36 – Temperature profiles from 77 to 5 K of Mössbauer spectra of transformed Fh-Zn samples initially containing 5 wt% Zn, reacted in sediments from Friedrichskoog (FKS 5) for 0 (ini), sixteen (16B) and twenty-six weeks (core 26w). Each temperature of acquisition is represented by a given color, grading from red (77 K) to blue (5 K). Arrows on the right-hand side indicate the temperature at which a sextet appears (gray arrow) and is fully ordered (black arrow).

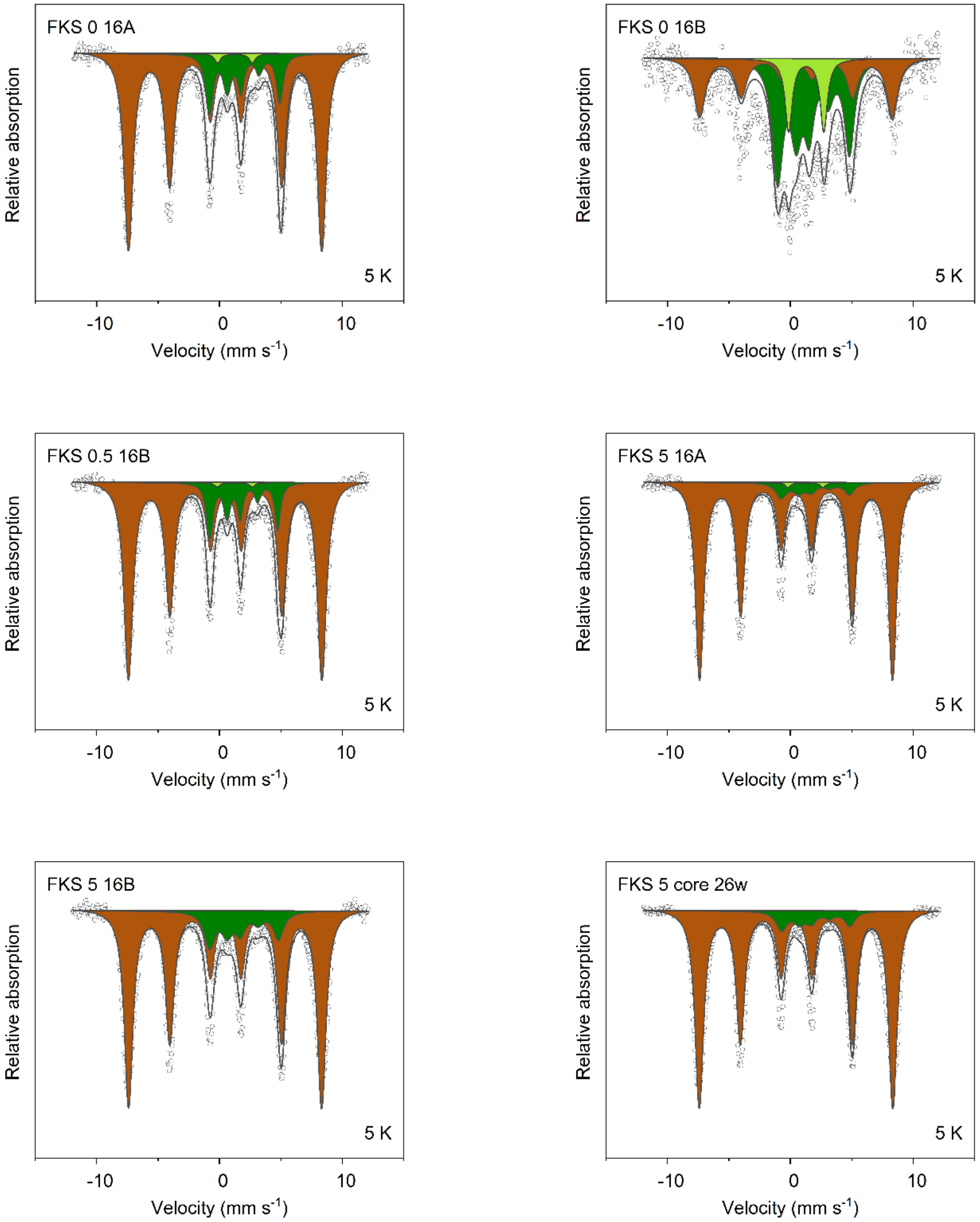

Figure S37 – Mössbauer spectra at 5 K of a selection of transformed Fh-Zn samples reacted in sediments from Friedrichskoog (FKS) for 16 weeks. The experimental data is shown as open circles, the calculated fit as a black line, and the fitting components as filled areas: brown for the Fe(III) sextet, light green for the Fe(II) doublet, dark green for the Fe(II) octet (Table S14).

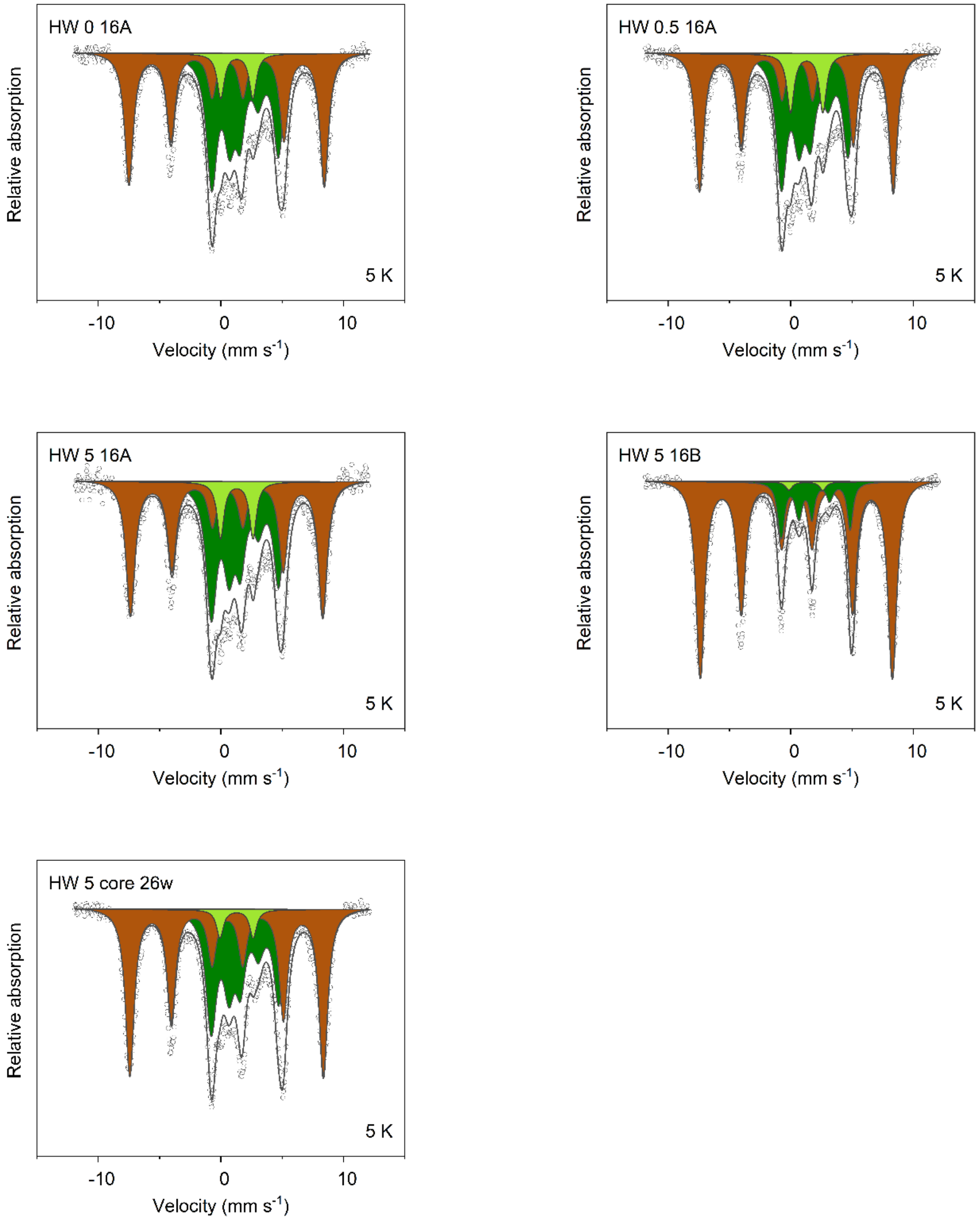

Figure S38 – Mössbauer spectra at 5 K of a selection of transformed Fh-Zn samples reacted in sediments from Hollerwettern (HW) for 16 weeks. The experimental data is shown as open circles, the calculated fit as a black line, and the fitting components as filled areas: brown for the Fe(III) sextet, light green for the Fe(II) doublet, dark green for the Fe(II) octet (Table S14).

Table S14 – Fitting parameters (FSH model) of Mössbauer spectra acquired at 5 K of transformed Fh-Zn samples reacted in the FKS and HW mesocosms. CS: center/isomer shift, H: Hyperfine field, $e^2qQ/2$: electric quadrupole interaction parameter, η: asymmetry parameter, w: half-line at half maximum, φ and θ: azimuthal and polar angle (respectively) between the electric field gradient (EFG) axis of symmetry and the hyperfine field H, χ^2_R: reduced chi-square. Fe(III) S1: sextet, Fe(II) D1: doublet.

Sample	Component	Contribution (%)	CS (mm/s)	H (T)	$e^2qQ/2$ (mm/s)	η	w (mm/s)	Φ (°)	Θ (°)	χ^2_R
**FKS 0 16A**	Fe(III) S1	82.4(8)	0.475	48.8	-0.04	-	0.44	-	-	3.10
	Octet	16.3(6)	1.61	12.2	-3.1	0.2*	0.33	84*	95*	
	Fe(II) D1	1.3(3)	1.26*	-	2.82*	-	0.29*	-	-	
**FKS 0 16B**	Fe(III) S1	34(3)	0.49*	48.6	-0.08	-	0.55	-	-	0.95
	Octet	52(2)	1.44	12.6	-3.1	0.2*	0.5*	84*	95*	
	Fe(II) D1	14(1)	1.29*	-	2.86*	-	0.35*	-	-	
**FKS 0.5 16B**	Fe(III) S1	85.5(6)	0.493	48.8	-0.05	-	0.43	-	-	5.09
	Octet	13.9(4)	1.56	11.7	-3.01	0.2*	0.28	84*	95*	
	Fe(II) D1	0.6(3)	1.24*	-	2.85*	-	0.26*	-	-	
**FKS 5 16A**	Fe(III) S1	92.8(7)	0.481	48.7	-0.03	-	0.40	-	-	6.19
	Octet	7.2(6)	1.61	12.0	-2.97	0.2*	0.5*	84*	95*	
**FKS 5 16B**	Fe(III) S1	84.8(7)	0.484	48.7	-0.04	-	0.42	-	-	4.17
	Octet	15.2(6)	1.58	11.9	-3.04	0.2*	0.5*	84*	95*	
**FKS 5 core 26w**	Fe(III) S1	91.3(4)	0.483	48.9	-0.03	-	0.41	-	-	12.9
	Octet	8.7(4)	1.64	12.1	-2.95	0.2*	0.5*	84*	95*	
**HW 0 26A**	Fe(III) S1	48.2(6)	0.506	49.4	-0.07	-	0.42	-	-	3.86
	Octet	45.1(5)	1.56	12.0	-2.83	0.2*	0.5*	84*	95*	
	Fe(II) D1	6.7(3)	1.30*	-	2.62*	-	0.35*	-	-	
**HW 0.5 16A**	Fe(III) S1	47.4(6)	0.497	49.0	-0.07	-	0.41	-	-	3.18
	Octet	44.0(5)	1.54	11.8	-2.89	0.2*	0.5*	84*	95*	
	Fe(II) D1	8.6(4)	1.30*	-	2.64*	-	0.35*	-	-	
**HW 5 16A**	Fe(III) S1	48.5(8)	0.505	48.6	-0.08	-	0.44	-	-	2.30
	Octet	43.7(7)	1.54	12.0	-2.89	0.2*	0.5*	84*	95*	
	Fe(II) D1	7.9(5)	1.29*	-	2.65*	-	0.34*	-	-	
**HW 5 16B**	Fe(III) S1	84.0(5)	0.479	48.6	-0.04	-	0.43	-	-	7.48
	Octet	14.6(4)	1.61	12.0	-3.04	0.2*	0.29	84*	95*	
	Fe(II) D1	1.4(2)	1.24*	-	2.77*	-	0.32*	-	-	
**HW 5 core 26w**	Fe(III) S1	57.2(5)	0.501	49.0	-0.07	-	0.43	-	-	4.40
	Octet	38.9(4)	1.55	12.0	-2.91	0.2*	0.5*	84*	95*	
	Fe(II) D1	3.9(3)	1.28*	-	2.71*	-	0.34*	-	-	
**Average**	Fe(III) S1		0.49(1)	48.8(2)	-0.05(2)	-	0.43(4)	-	-	
	Octet		1.57(5)	12.0(2)	-2.98(9)	0.2*	0.45(9)	84*	95*	

13. Fe bulk XAS

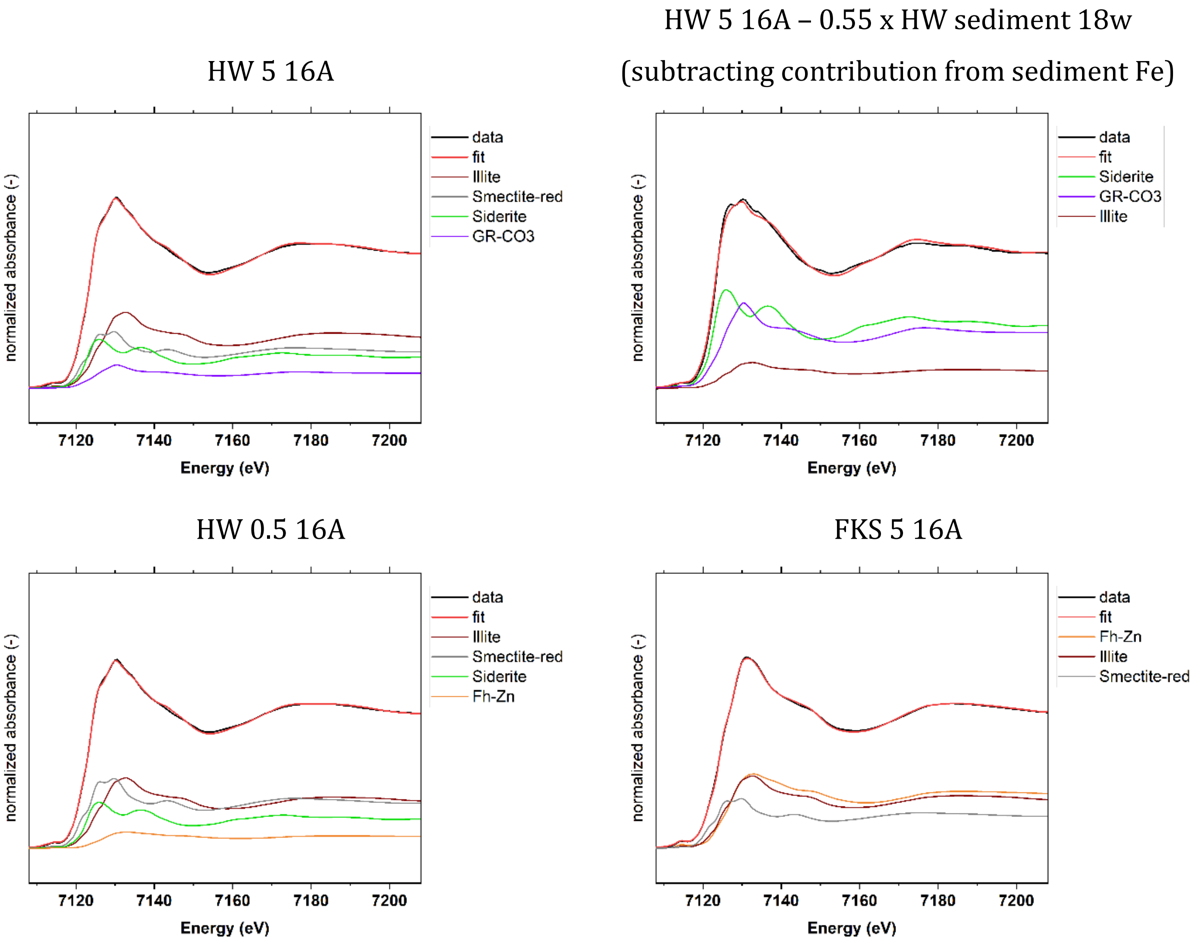

Figure S39 – Fe K-edge bulk EXAFS spectra of transformed Fh-Zn:sediment mixes HW 5 16A (top left), HW 5 16A after subtracting the contribution (55 %) from HW sediment (top right), HW 0.5 16A (bottom left) and FKS 5 16A (bottom right). The experimental data and corresponding LC fits are shown in black and red, respectively. The weighted spectra of fitted reference compounds are shown below in dark red for illite, gray for reduced smectite, green for siderite, orange for ferrihydrite (Fh-Zn) and purple for carbonated green rust (GR-CO3).

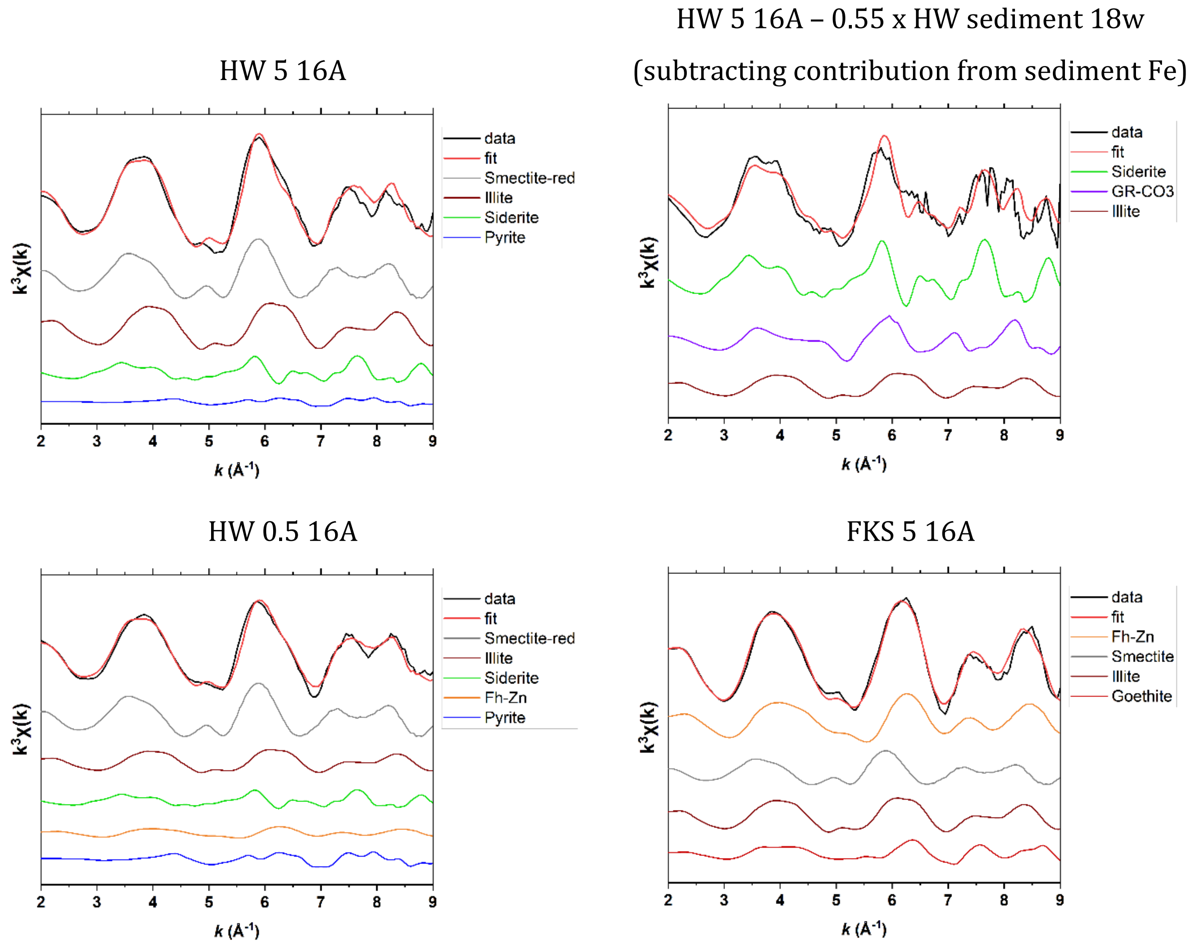

Figure S40 – Fe K-edge bulk EXAFS spectra of transformed Fh-Zn:sediment mixes HW 5 16A (top left), HW 5 16A after subtracting the contribution (55 %) from HW sediment (top right), HW 0.5 16A (bottom left) and FKS 5 16A (bottom right). The experimental data and corresponding LC fits are shown in black and red, respectively. The weighted spectra of fitted reference compounds are shown below in dark red for illite, gray for reduced smectite, green for siderite, orange for ferrihydrite (Fh-Zn), purple for carbonated green rust (GR-CO3), blue for pyrite and dark orange for goethite.

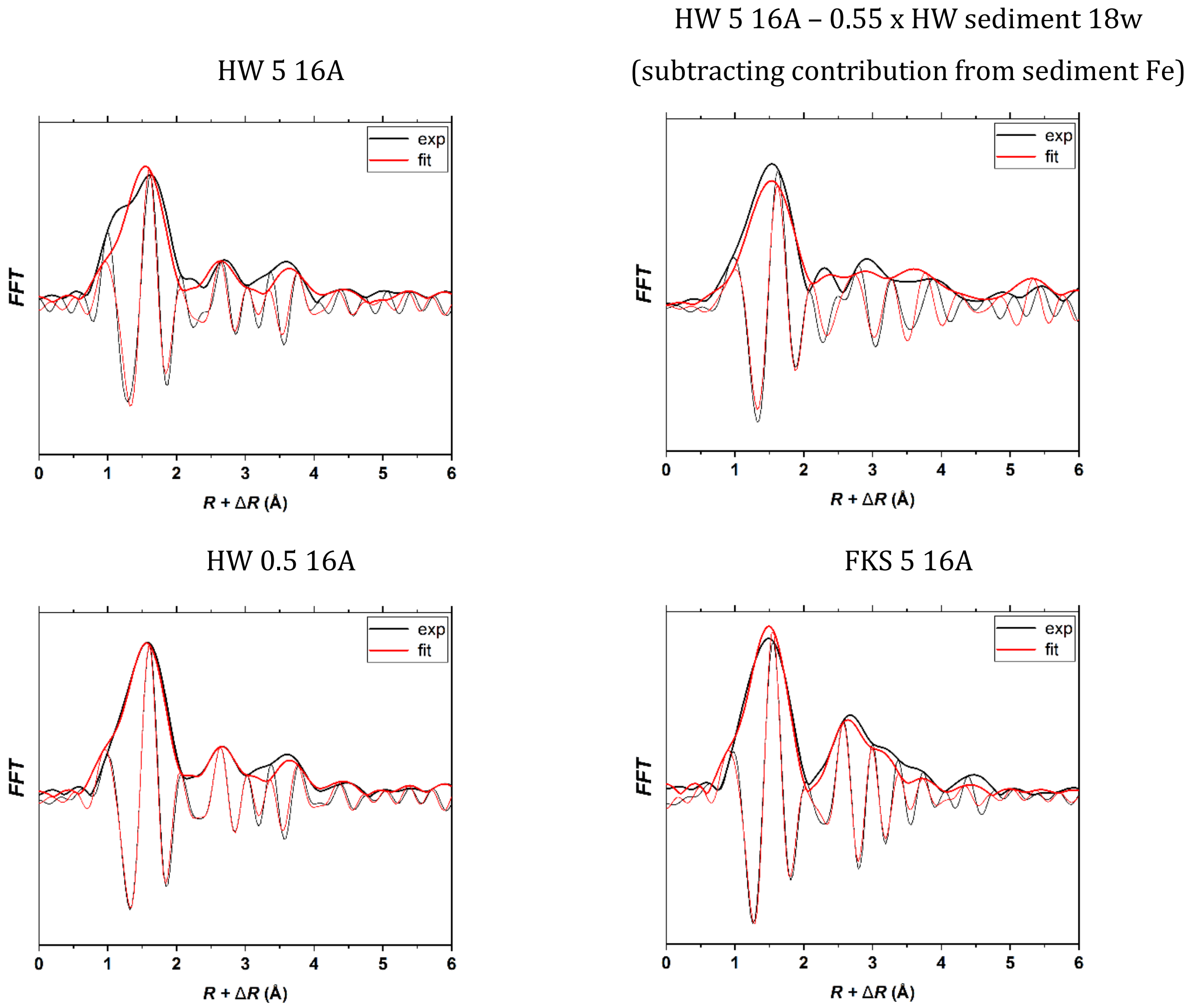

Figure S41 – Fast Fourier Transforms (FFT) of Fe K-edge bulk EXAFS spectra of transformed Fh-Zn:sediment mixes HW 5 16A (top left), HW 5 16A after subtracting the contribution (55 %) from HW sediment (top right), HW 0.5 16A (bottom left) and FKS 5 16A (bottom right). The experimental data and corresponding LC fits are shown in black and red, respectively.

Table S15 – Fe K-edge XAS fitting parameters of transformed Fh-Zn:sediment mixes HW 5 16A (before and after subtracting the contribution (55 %) from HW sediment), HW 0.5 16A and FKS 5 16A . Ill: illite, Smec: smectite (reduced), Py: pyrite, Fh: Fh-Zn 5%, Sid: siderite, GR: CO_3-Green rust, Gt: goethite. %Fe(II): percentage of Fe(II) over total Fe, calculated from the weighted Fe(II) content of fitting components. Rf: R-factor, χ^2_R: reduced chi-square.

		Components								Fit parameters	
**Sample**	**Fit**	**Ill.**	**Smec.**	**Py.**	**Fh**	**Sid**	**GR**	**Gt**	**% Fe(II)**	**R_f**	**χ^2_R**
**HW 5 16A**	**XANES**	38(1)	28(1)	-	-	23.4(7)	11(2)	-	62	$3.1 .10^{-4}$	$5.6 .10^{-5}$
	**EXAFS**	36(2)	47(2)	3(1)	-	14(1)	-	-	71	$5.1 .10^{-2}$	0.23
**HW 5 16A -HW sed**	**XANES**	13(2)	-	-	-	47(1)	40(3)	-	63	$21 .10^{-4}$	$40 .10^{-5}$
	**EXAFS**	25(4)	-	-	-	45(3)	30(4)	-	60	$24 .10^{-2}$	0.75
**HW 0.5 16A**	**XANES**	35(2)	34.2(8)	-	9(2)	22.1(6)	-	-	63	$3.0 .10^{-4}$	$5.3 .10^{-5}$
	**EXAFS**	22(6)	51(3)	6.0(9)	10(5)	12(1)	-	-	72	$3.4 .10^{-2}$	0.13
**FKS 5 16A**	**XANES**	36(2)	24.4(4)	-	40(1)	-	-	-	31	$2.0 .10^{-4}$	$3.7 .10^{-5}$
	**EXAFS**	26(4)	26(2)	-	36(3)	-	-	12(2)	31	$2.1 .10^{-2}$	0.10

14. Micro-XRF of transformed sample HW 5 16A

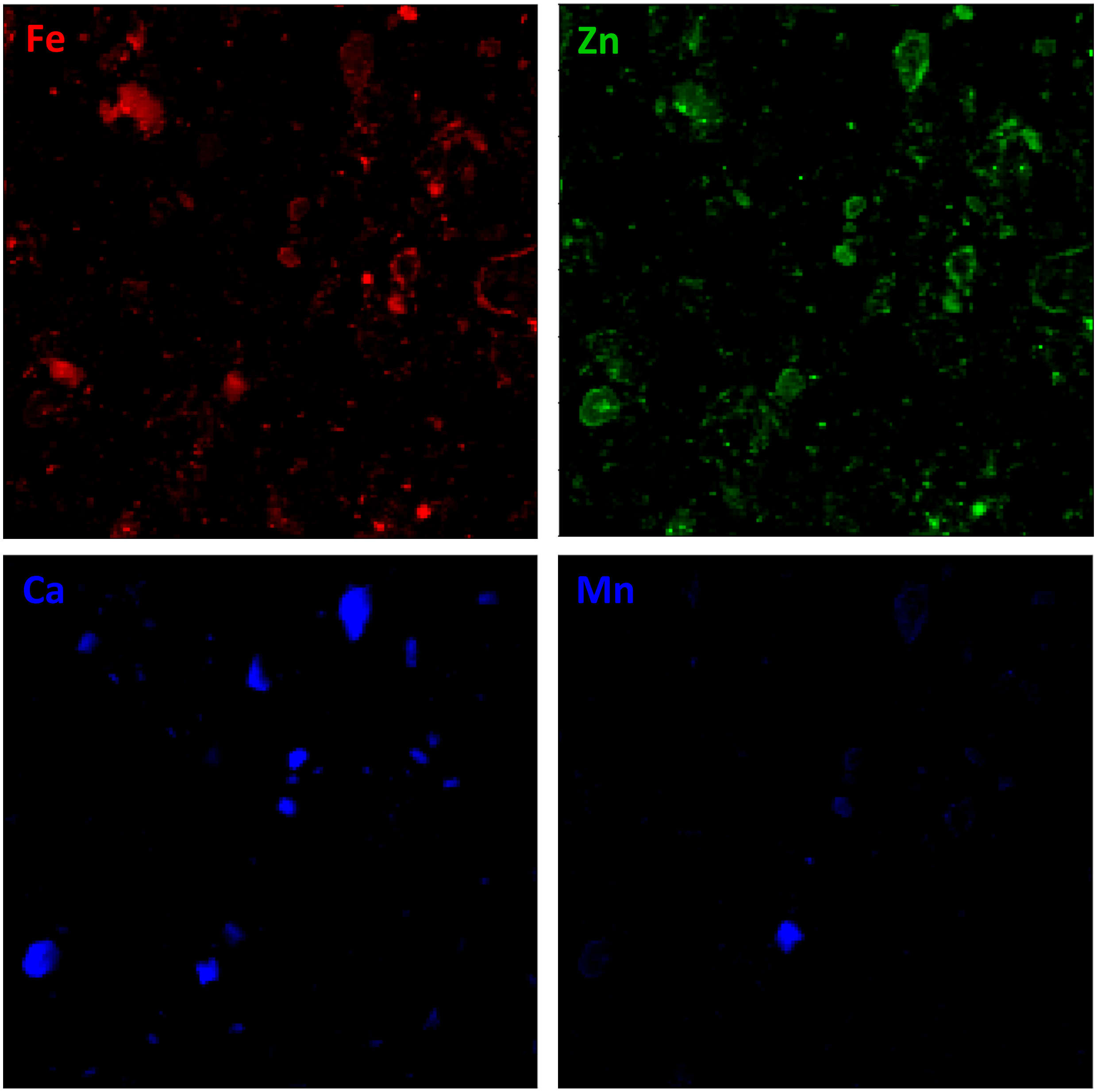

Figure S42 – Micro-X-ray-fluorescence maps of sample HW 5 16A (Fh-Zn 5 % in HW sediment reacted for 16 weeks): (top left) Fe in red, (top right) Zn in green, (bottom left) Ca in blue and (bottom right) Mn in blue.

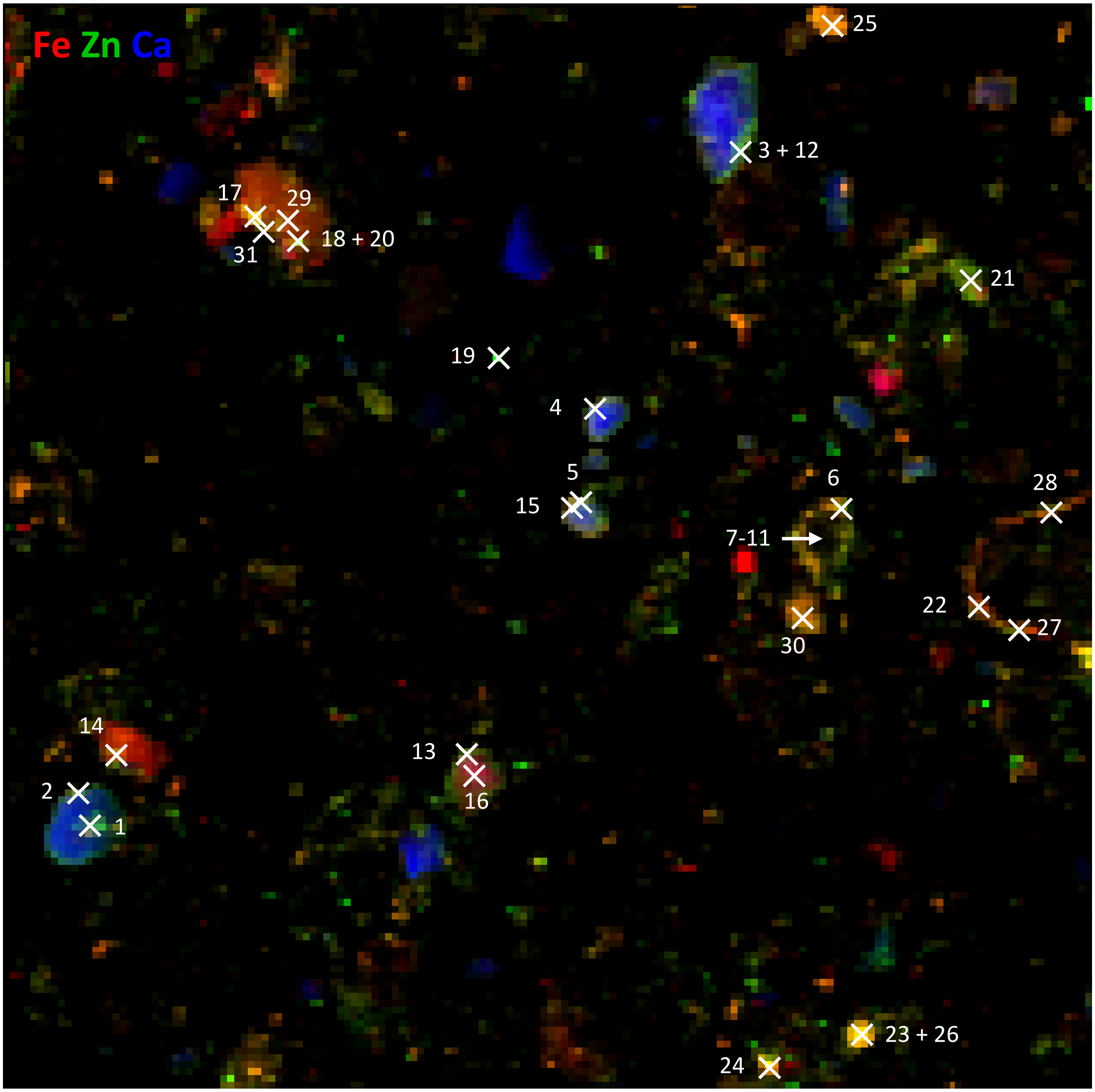

Figure S43 – Micro-XRF mapping of sample HW 5 16A (Fh-Zn 5 % in HW sediment reacted for 16 weeks), showing Fe fluorescence in red, Zn fluorescence in green and Ca fluorescence in blue. Numbered crosses show spots where Zn K-edge and/or Fe K-edge micro-XANES spectra were acquired. Spectra 7 to 11 were taken on a transect crossing a Zn- and Fe-rich coating with 5 µm steps.

15. Fe K-edge micro-XANES of transformed sample HW 5 16A

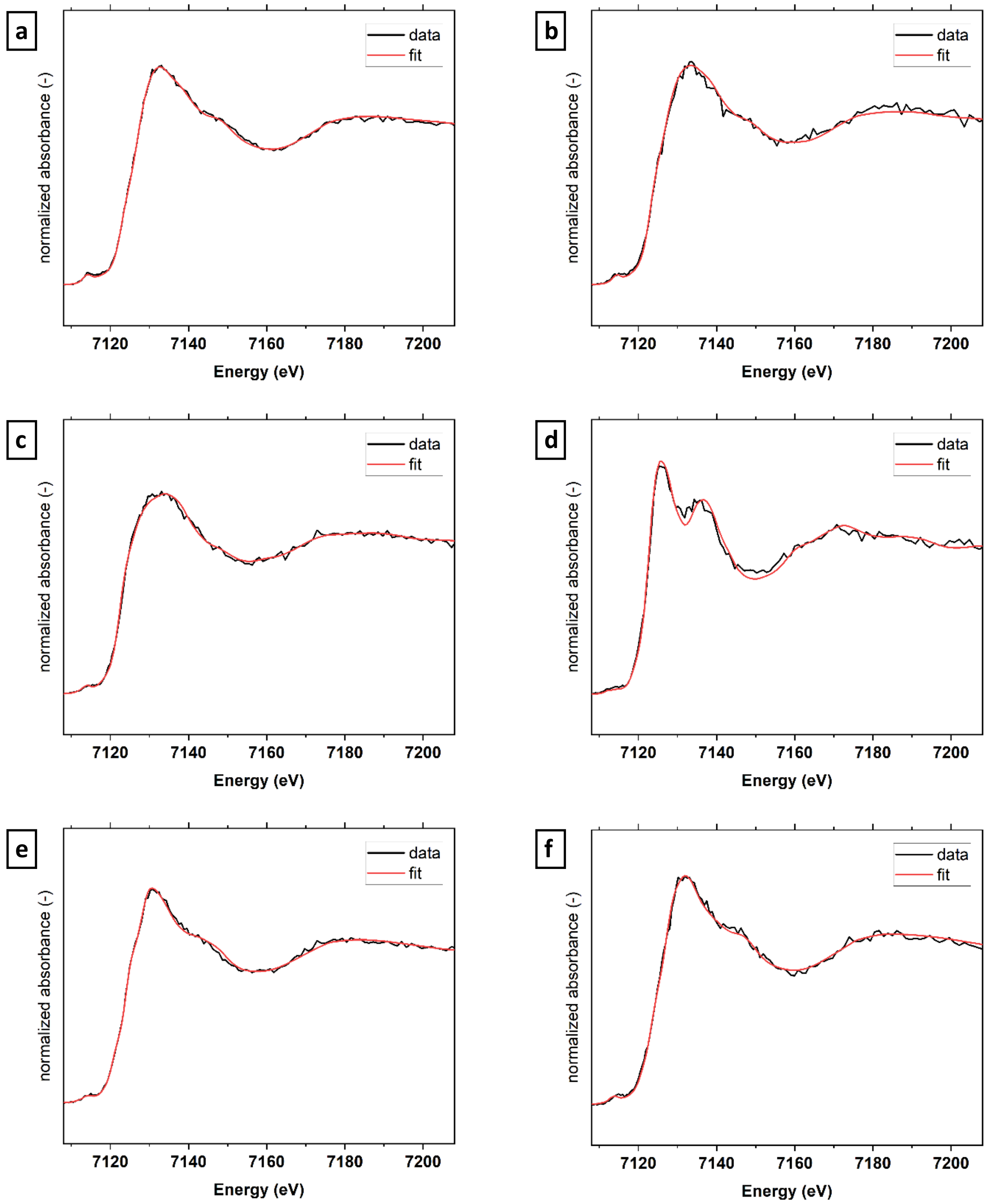

Figure S44 – Examples of Fe K-edge micro-XANES spectra (black) and corresponding LC fits (red) acquired on Fh-Zn:sediment sample HW 5 16A: (a) point 25b composed of Fh-Zn with additional 6 % of siderite, (b) point 27 fit with Fh-Zn with additional 20 % of siderite, (c) point 21a composed of Fh-Zn with additional 35 % of siderite, (d) point 15 consisting of siderite only; (e) point 30 fit with 37 % Fh-Zn, 30 % illite and 33 % reduced smectite; (f) point 22 composed of 49 % Fh-Zn and 51 % illite.

Table S16 – Fe K-edge micro-XANES LC fitting parameters of Fh-Zn:sediment sample HW 5 16A. Rf: R-factor; χ^2_R: reduced chi-square; ΔE_0: shift in E_0 applied to the experimental spectrum to compensate for the absence of energy calibration.

	Component proportions (%)					Fit parameters		
**Point**	**Fh-Zn**	**Siderite**	**Illite**	**Smectite reduced**	**Fe(II)-citrate**	R_f ($x10^{-3}$)	χ^2_R ($x10^{-4}$)	**ΔE0 (eV)**
**13**	-	100	-	-	-	10.9	17.2	-0.380(48)
**14**	-	-	72(3)	-	28(3)	5.0	9.4	-0.65(12)
**15**	-	100	-	-	-	6.1	10.3	-0.037(37)
**16**	-	100	-	-	-	10.9	17.5	-0.303(49)
**21a**	65(1)	35(1)	-	-	-	1.9	3.1	-0.480(59)
**21b**	70(1)	30(1)	-	-	-	2.3	3.8	-0.512(70)
**22**	49(6)	-	51(6)	-	-	2.1	4.2	-0.666(59)
**25a**	94(1)	6(1)	-	-	-	1.1	2.2	-0.065(49)
**25b**	94(1)	6(1)	-	-	-	0.7	1.4	-0.277(39)
**26**	94(1)	6(1)	-	-	-	1.0	1.8	-0.066(45)
**27**	80(2)	20(2)	-	-	-	3.2	6.3	0.036(90)
**30**	37(3)	-	30(4)	33(2)	-	1.2	2.1	-0.157(70)
**31**	38(3)	-	30(4)	31(2)	-	1.0	1.7	-0.047(63)

16. Zn bulk XAS

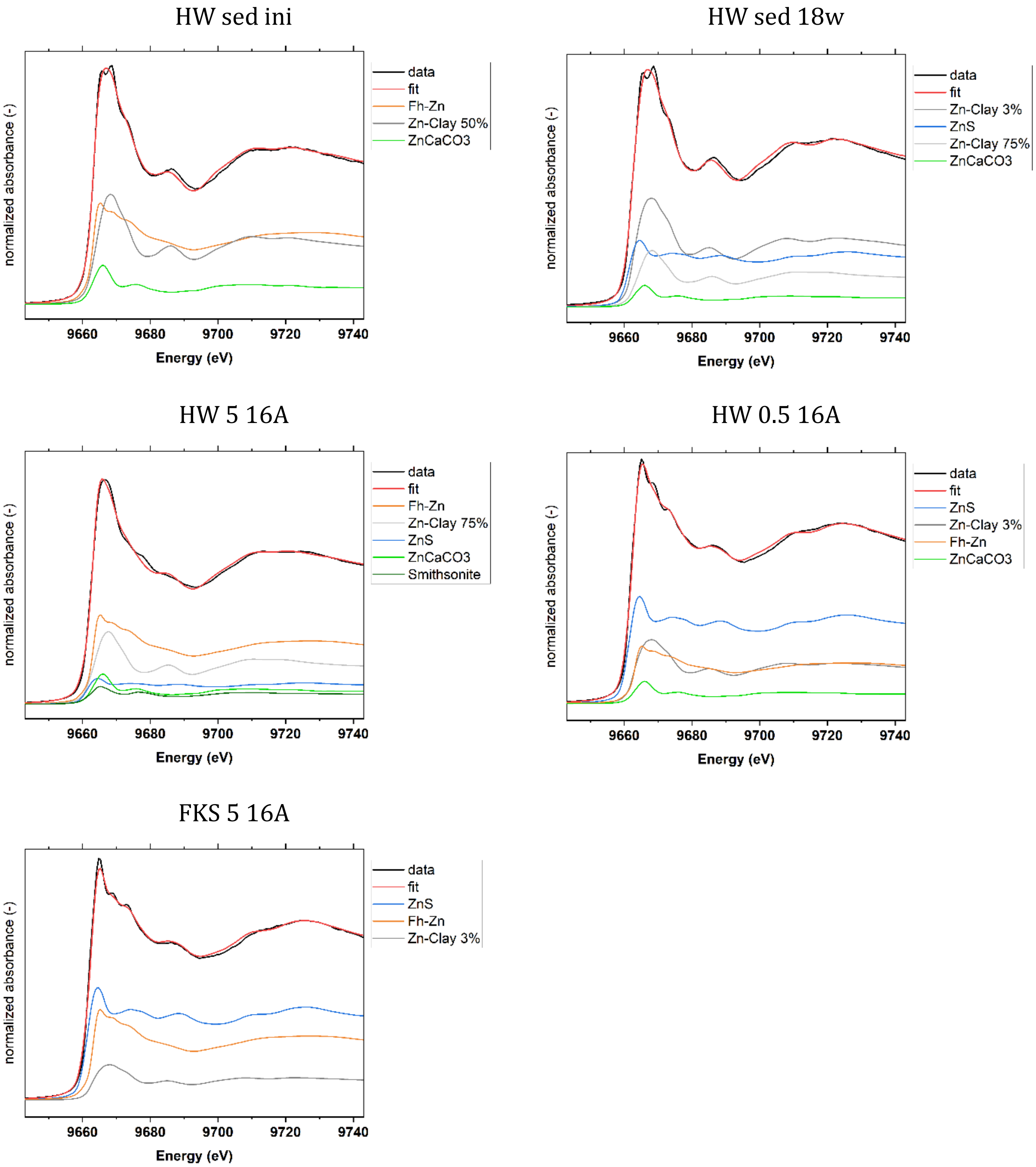

Figure S45 – Zn K-edge XANES spectra of bulk samples (black) and corresponding LC fits (red). The weighted fitting components (reference compounds) are shown with thin colored lines: ZnS in blue, Fh-Zn 5 wt% in orange, Zn-bearing calcium carbonate ("ZnCaCO3") in light green, Zn-Clay with 3, 50 or 75 wt% Zn loadings in gray, Smithsonite ($ZnCO_3$) in dark green.

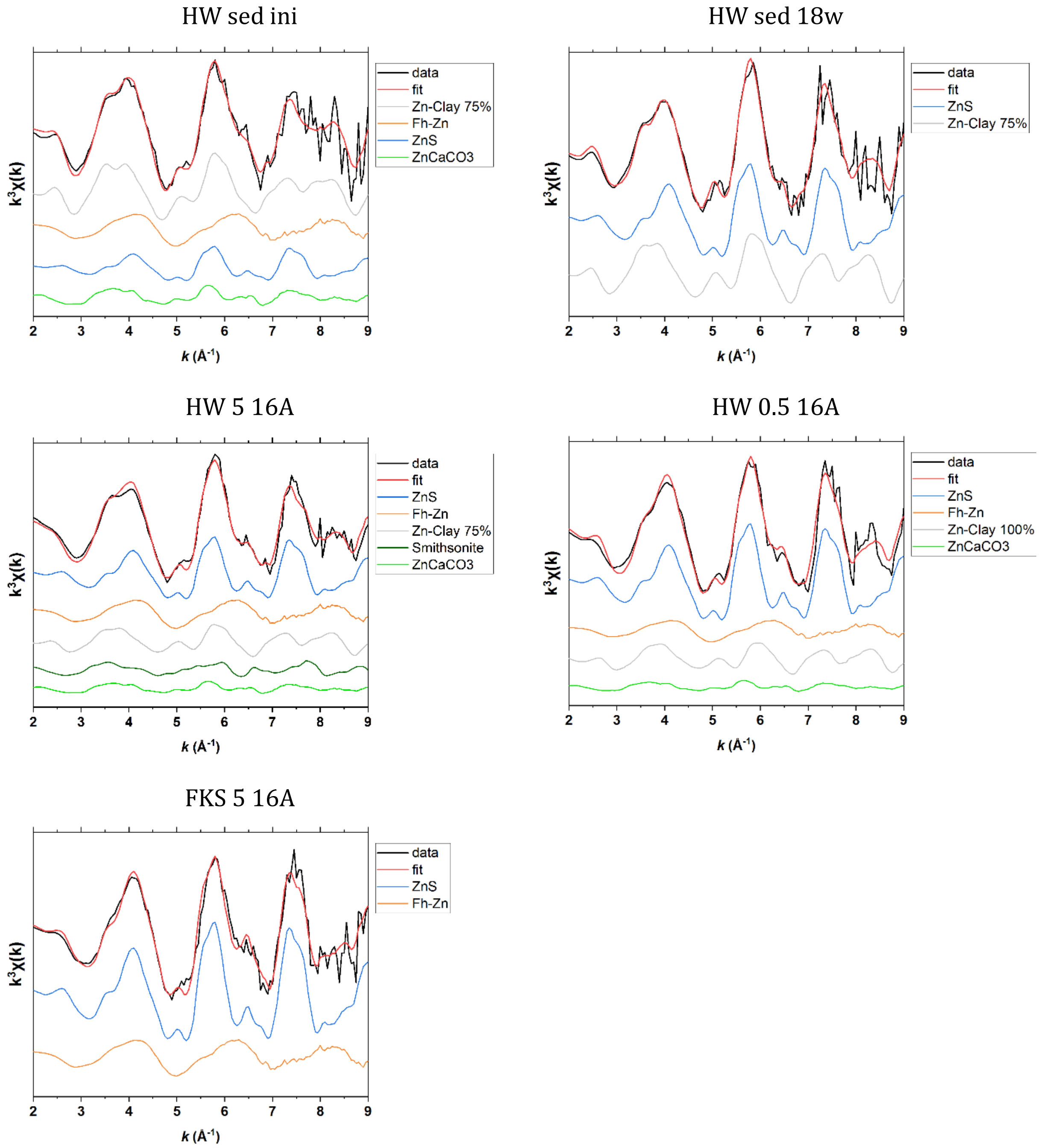

Figure S46 – Zn K-edge EXAFS spectra of bulk samples (black) and corresponding LC fits (red). The weighted fitting components (reference compounds) are shown with thin colored lines: ZnS in blue, Fh-Zn 5 wt% in orange, Zn-bearing calcium carbonate ("ZnCaCO3") in light green, Zn-Clay with 75 or 100 wt% Zn loadings in gray, Smithsonite ($ZnCO_3$) in dark green.

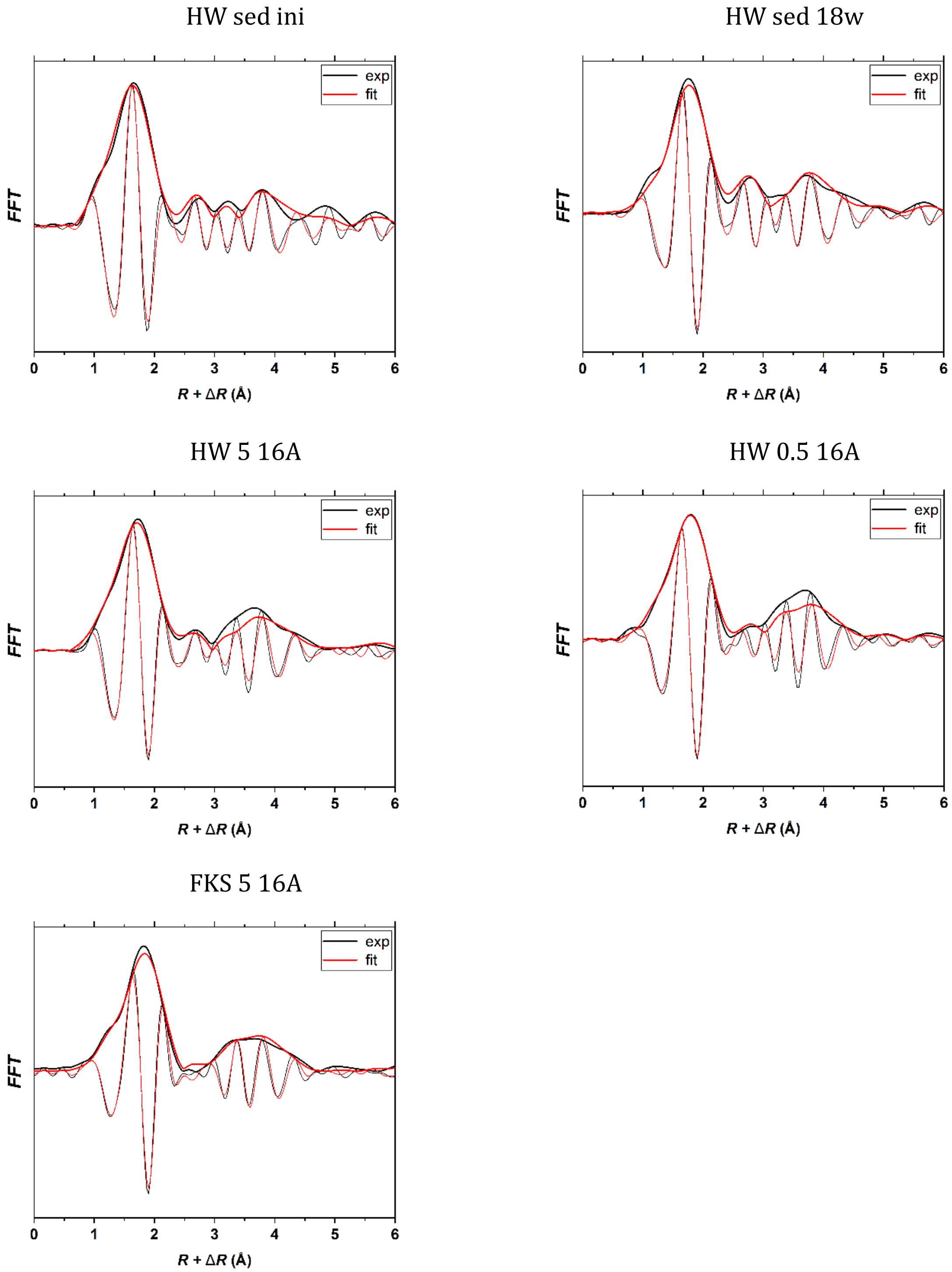

Figure S47 – Fast Fourier Transforms (FFT) of Zn K-edge EXAFS spectra of bulk samples (black) and corresponding LC fits (red).

Table S17 – Zn K-edge bulk XAS fitting parameters of HW initial dry sediment, HW sediment reduced after 18 weeks, and transformed Fh-Zn:sediment mixes HW 5 16A, HW 0.5 16A and FKS 5 16A . ZnS: sphalerite, Fh-Zn: Fh-Zn 5 %, $CaCO_3$: trace Zn in calcite (1500 ppm), $ZnCO_3$: smithsonite, Zn-Clay x% (x = 3, 50, 75 or 100): synthetic kerolite-type phyllosilicate with x % Zn and (100-x) % Mg. R_f: R-factor; χ^2_R: reduced chi-square.

		Components						Fit parameters	
**Sample**	**Fit**	**ZnS**	**Fh-Zn**	**$CaCO_3$**	**$ZnCO_3$**	**Zn-Clays (*Zn%*)**		**R_f**	**χ^2_R**
**HW sediment ini**	**XANES**	-	46(1)	12(1)	-	*50%*	42(12)	2.1 $.10^{-3}$	5.0 $.10^{-4}$
	**EXAFS**	20(3)	27(4)	11(4)	-	*75%*	42(4)	0.108	1.35
**HW sediment 18w**	**XANES**	32(1)	-	6(1)	-	*3%*<br>*75%*	41(5)<br>20(4)	2.1 $.10^{-3}$	5.0 $.10^{-4}$
	**EXAFS**	58(3)	-	-	-	*75%*	42(3)	0.092	1.75
**HW 5 16A**	**XANES**	14(2)	42(2)	9(2)	7(3)	*75%*	28(1)	1.5 $.10^{-3}$	3.3 $.10^{-4}$
	**EXAFS**	37(2)	25(2)	7(3)	11(2)	*75%*	19(2)	0.043	0.48
**HW 0.5 16A**	**XANES**	49(1)	23(2)	6(1)	-	*3%*	22(2)	1.2 $.10^{-3}$	2.5 $.10^{-4}$
	**EXAFS**	57(3)	19(3)	6(3)	-	*100%*	18(3)	0.062	0.99
**FKS 5 16A**	**XANES**	51(1)	36(3)	-	-	*3%*	12(2)	1.6 $.10^{-3}$	3.2 $.10^{-4}$
	**EXAFS**	69(2)	31(3)	-	-	-	-	0.072	1.06

17. Zn K-edge micro-XANES of transformed sample HW 5 16A

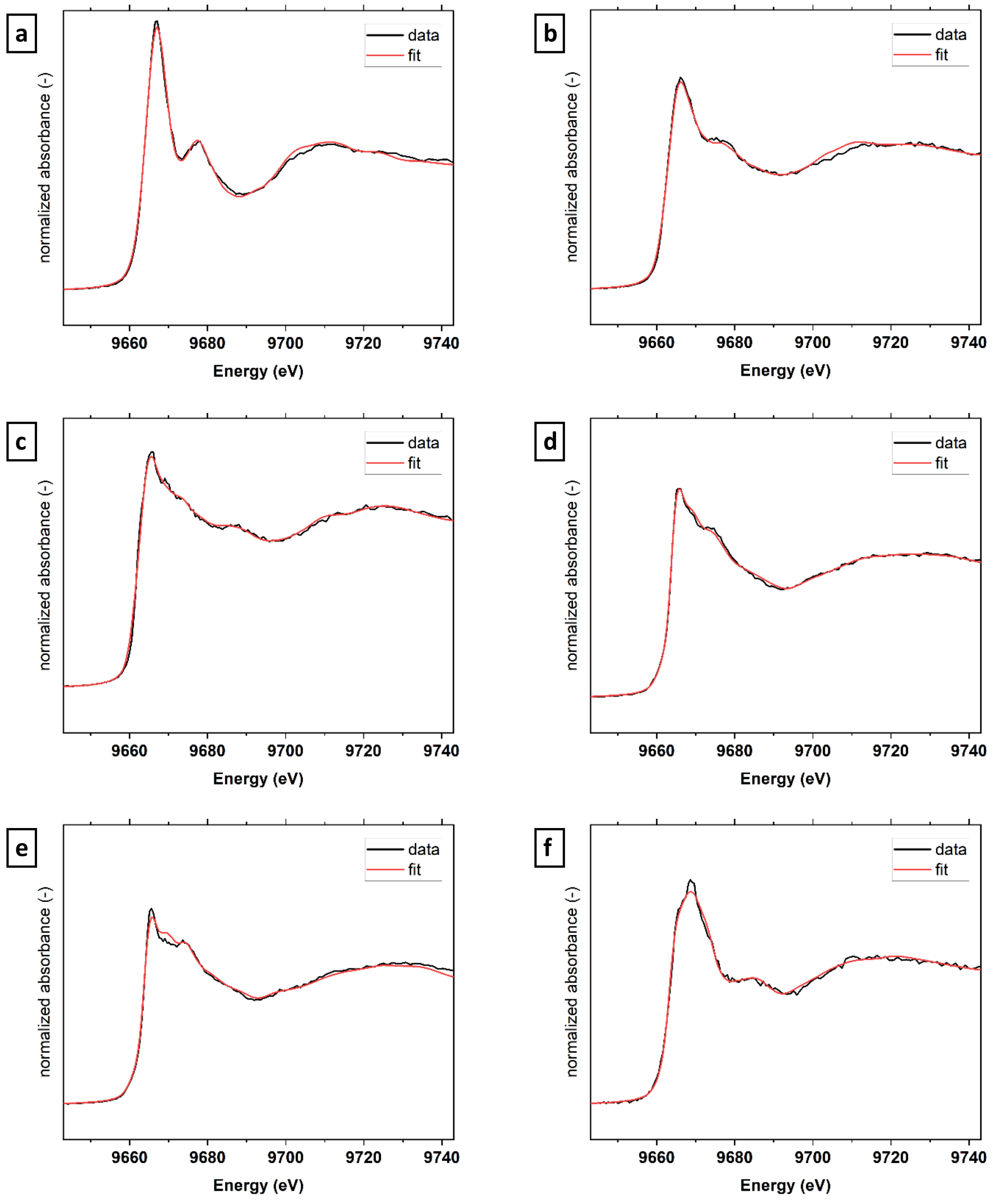

Figure S48 – Examples of Zn K-edge micro-XANES spectra (black) and corresponding LC fits (red) acquired on Fh-Zn:sediment sample HW 5 16A: (a) point 5 composed of zinc carbonates; (b) point 6 consisting of a mixture of Zn carbonates and ZnS; (c) point 18 composed of ZnS with Zn carbonate and Fh-Zn; (d) point 25a dominated by Fh-Zn with trace Zn carbonate; (e) point 24 with Zn in Fe (oxyhydr-)oxides, Fh-Zn and franklinite; (f) point 29 composed of LDH and Fh-Zn.

Table S18 – Zn K-edge micro-XANES LC fitting parameters of Fh-Zn:sediment sample HW 5 16A. The points are grouped depending on their main fitting reference compounds. $CaCO_3$: trace Zn in calcite (1500 ppm), $ZnCO_3$: smithsonite, Hydroz.: hydrozincite $Zn_5(CO_3)_2(OH)_6$; Frankl.: franklinite $ZnFe_2O_4$; Zn-Clay 50%: synthetic kerolite-type phyllosilicate with 50 % Zn and 50 % Mg; LDH: Zn layered double hydroxide. Rf: R-factor; χ^2_R: reduced chi-square; ΔE_0: shift in E_0 applied to the experimental spectrum to compensate for the absence of energy calibration.

	Component proportions (%)				Fit parameters		
**Point**	**$CaCO_3$**	**$ZnCO_3$**	**Hydroz.**	**ZnS**	**R_f** (x10^{-3})	**χ^2_R** (x10^{-4})	**ΔE0 (eV)**
**1**	46(1)	38(2)	17(2)	-	1.3	2.9	0.675(23)
**2**	25(2)	53(2)	22(2)	-	1.9	3.8	0.621(29)
**3**	100	-	-	-	6.2	15.7	0.956(28)
**4**	52(1)	30(2)	18(2)	-	1.7	3.9	0.881(25)
**5**	61(2)	39(2)	-	-	1.8	4.3	1.220(18)
**6**	-	36(2)	35(2)	29(1)	1.8	3.7	0.543(36)
**7**	-	57(6)	43(6)	-	5.5	10.3	0.699(62)
**8**	33(2)	51(3)	16(2)	-	2.9	7.7	1.800(36)
**9**	67(2)	33(2)	-	-	2.9	7.3	1.908(22)
**10**	59(2)	16(3)	25(2)	-	2.4	6.0	1.699(31)
**11**	25(2)	38(2)	37(2)	-	1.9	4.2	1.631(32)
**12**	100	-	-	-	5.4	11.3	1.138(36)
**13**	40(2)	60(2)	-	-	2.1	4.8	1.476(20)
**14**	23(2)	22(2)	56(2)	-	1.9	4.1	1.108(33)
**15**	60(2)	40(2)	-	-	2.0	5.0	1.732(19)
**16**	52(2)	48(2)	-	-	2.5	6.0	1.634(21)
	**Component proportions (%)**				**Fit parameters**		
**Point**	**Fh-Zn**	**Hydroz.**	**$CaCO_3$**	**ZnS**	**R_f**	**χ^2_R**	**ΔE0 (eV)**
**17**	35(5)	47(4)	-	19(3)	3.9	8.2	0.031(46)
**18**	16(3)	25(2)	-	59(2)	1.8	3.2	0.266(36)
**19**	22(4)	-	12(2)	66(3)	5.9	11.0	0.315(62)
**20**	43(4)	19(3)	-	38(2)	2.8	5.6	0.033(41)
**21**	-	22(2)	-	78(2)	4.0	7.6	0.400(44)
**22**	23(4)	61(3)	-	16(2)	2.6	5.8	0.263(38)

	Component proportions (%)				Fit parameters		
**Point**	**Fh-Zn**	**Frankl.**	**$CaCO_3$**	**Hydroz.**	**R_f**	**χ^2_R**	**ΔE0 (eV)**
**23**	61(4)	39(4)	-	-	3.4	6.3	0.152(43)
**24**	72(3)	28(3)	-	-	2.5	4.8	0.342(36)
**25a**	94	-	6(1)	-	0.9	1.9	0.526(16)
**25b**	93	-	7(1)	-	1.5	3.3	0.675(20)
**25c**	92	-	8(1)	-	1.6	3.4	0.776(21)
**26**	71(4)	29(4)	-	-	3.5	7.7	0.350(42)
**27**	-	41(1)	13(1)	46(2)	1.6	3.5	0.090(27)

	Component proportions (%)				Fit parameters		
**Point**	**Fh-Zn**	**Hydroz.**	**Zn-Clay 50%**	**LDH**	**R_f**	**χ^2_R**	**ΔE0 (eV)**
**28**	32(3)	-	-	68(2)	4.0	8.3	-0.113(36)
**29**	34(2)	-	-	66(2)	2.5	5.5	0.171(28)
**30**	-	-	100	-	4.4	10.8	-0.092(33)
**31**	38(3)	35(6)	27(3)	-	1.2	3.0	0.289(30)

*Table S19 – Comparison of average Zn and Fe speciation on micro-XANES points where spectra were acquired at both Zn and Fe K-edges. The contributions from LCF components (in italic) were grouped in main categories: Fe oxides, Carbonates, Clays and Sulfides. For points 21 and 25 where several spectra acquired on the same spot, the LCF results were averaged. The most likely interpretation of the observed speciation combined with the local spatial distribution of Fe and Zn is indicated below each point. * point 14 also contains 28 % of Fe fit as Fe(II)-citrate (see Table S16), not shown in this table.*

	**Fe oxides**		**Carbonates**		**Clays**		**Sulfides**	
	**Fe**	**Zn**	**Fe**	**Zn**	**Fe**	**Zn**	**Fe**	**Zn**
**Point**	*Fh-Zn*	*Fh-Zn, franklinite*	*siderite*	*(Ca,Zn)CO_3 hydroz.*	*illite, smectite*	*LDH, Zn-Clay 50 %*	**-**	*ZnS*
**13**	-	-	100	100	-	-	-	-
Interpretation: **Coating around siderite grain, fully carbonated**								
**14***	-	-	-	100	72	-	-	-
Interpretation: **Fe-rich clay with Zn-carbonate coating**								
**15**	-	-	100	100	-	-	-	-
Interpretation: **Coating around calcite grain, fully carbonated**								
**16**	-	-	100	100	-	-	-	-
Interpretation: **Siderite grain, fully carbonated – Zn likely in surface coating only**								
**21**	67	-	33	22	-	-	-	78
Interpretation: **Fh-Zn partly transformed into siderite, Zn mainly scavenged by sulfide**								
**22**	49	23	-	61	51	-	-	16
Interpretation: **Fh-Zn on clay partly transformed, Zn partitioning into carbonates and sulfides**								
**25**	94	93	6	7	-	-	-	-
Interpretation: **Not/slightly transformed Fh-Zn grain**								
**26**	94	100	6	-	-	-	-	-
Interpretation: **Not/slightly transformed Fh-Zn grain**								
**27**	80	41	20	59	-	-	-	-
Interpretation: **Fh-Zn partly transformed into siderite, Zn more carbonated than Fe**								
**30**	37	-	-	-	63	100	-	-
Interpretation: **Clay particle (likely from sediment)**								
**31**	38	38	-	35	62	27	-	-
Interpretation: **Fh-Zn on clay partly transformed, Zn partitioning into carbonates**								

18. HCl extraction of transformed Fh-Zn: Zn results

Some discrepancies were observed between the expected and measured amounts of Zn and Fe extracted with 0.5 M HCl from the sediments. The amount of Zn actually extracted from HW and FKS Control Core sediments was often higher than the expected maximal value measured by XRF (Figure S12c). In HW sediments, where the Zn concentration was measured at 76 µg/g, the amounts of extracted Zn were equivalent to actual minimal concentrations of 66 to 139 µg/g (if all Zn was HCl-extracted). In FKS sediments, where XRF indicates 45 µg/g Zn, the extracted Zn was equivalent to minimum 57-134 µg/g. These discrepancies may be attributed to propagated analytical uncertainties that could be larger than expected and/or to an undetected Zn contamination of the HCl solutions (not seen in analytical blanks).

To assess the extractability of Zn and compare it to that of Fe, we therefore used two hypotheses, referred to as “Measured” and “SedExt”. In the “Measured” hypothesis, we used only actually measured Zn concentrations in HCl solutions (even if apparently exceeding the XRF-measured sediment Zn) and the measured ratio of Fe coming from transformed Fh-Zn or from the sediment (measured with isotope ratios). In the “SedExt” hypothesis, we used the Zn and Fe values obtained from HCl extraction of the Control Core sediments. The initial Zn concentration in sediments was estimated with the maximal amount of extracted Zn, corresponding to 139 µg/g in HW sediments and 134 µg/g in FKS sediments. The fraction of Fe extracted from transformed Fh-Zn was estimated after subtracting the contribution from extracted sedimentary Fe, this time estimated using the initial sediment amount in Fh-Zn:sediment mixes and the fraction of Fe extracted from Control Core sediments as a background.

The results of Zn extraction by 0.5 M HCl depending on both hypotheses are presented in Figure S49. In the “Measured” hypothesis, extracted Zn ratios are higher than 100 % in transformed Zn-free Fh-Zn:sediment mixes, but < 100 % in Fh-Zn:sediment mixes with Fh-Zn 0.5 or 5 % (Figure S49a, b). The calculated proportions of Zn extracted from Fh-Zn transformation products (using mass balance equations) are comparable between both hypotheses (Figure S49c, d). However, the comparison of Fe and Zn extractabilities (ratio of Zn on Fe extracted proportions) gives opposite results. (Figure S49e, f) In the “Measured” hypotheses, this ratio is > 1, indicating that Zn was more easily extracted than Fe, while in the “SedExt” hypothesis, this ratio is < 1, meaning that Zn is less prone to HCl extraction than Fe. Overall, given that these ratios are anyway rather close to 1, we hypothesize that Zn and Fe are similarly prone to dissolution by HCl.

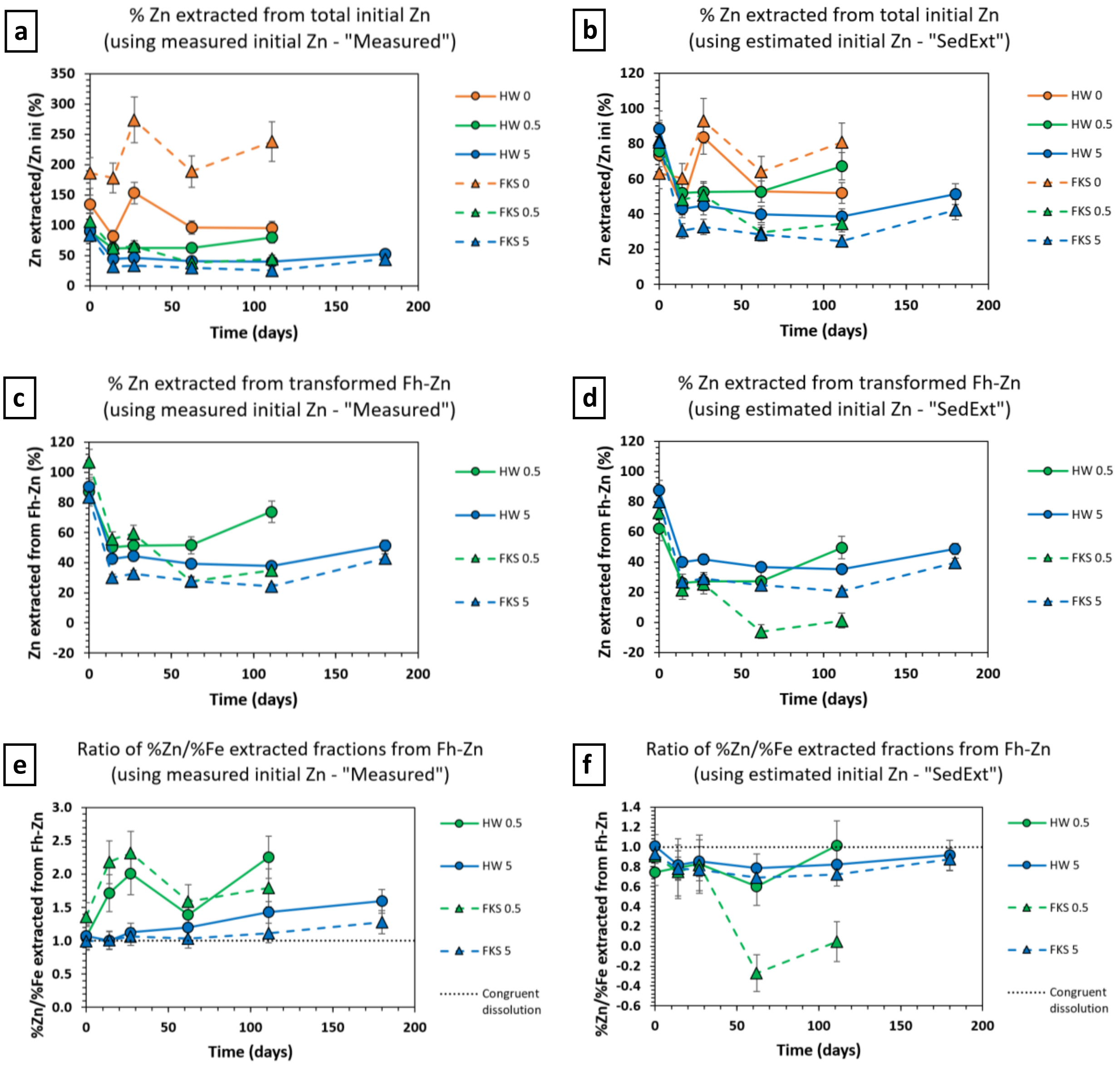

Figure S49 – Zn data from HCl extractions of transformed Fh-Zn:sediment mixes: (a) proportion of HCl-extracted Zn compared to total Zn in the Fh-Zn:sediment mix, using sediment Zn measured by XRF ("Measured" hypothesis); (b) proportion of HCl-extracted Zn compared to total Zn in the Fh-Zn:sediment mix, using HCl-extracted Zn from sediment ("SedExt" hypothesis); (c) proportion of total extracted Zn coming from transformed Fh-Zn ("Measured" hypothesis); (d) proportion of total extracted Zn coming from transformed Fh-Zn ("SedExt" hypothesis); (e) ratio of Zn proportions on Fe proportions HCl-extracted from Fh-Zn ("Measured" hypothesis); (f) ratio of Zn proportions on Fe proportions HCl-extracted from Fh-Zn ("SedExt" hypothesis). In (e) and (f), the horizontal black dashed line indicates a ratio of 1, which corresponds to "congruent dissolution", i.e., equivalent extractability towards HCl of Zn and Fe from transformed Fh-Zn.

References

Blaes, N., Fischer, H., Gonser, U., 1985. Analytical expression for the Mössbauer line shape of 57Fe in the presence of mixed hyperfine interactions. Nuclear Instruments and Methods in Physics Research Section B: Beam Interactions with Materials and Atoms 9, 201–208. https://doi.org/10.1016/0168-583X(85)90683-4

Cline, J.D., 1969. Spectrophotometric determination of hydrogen sulfide in natural waters 1. Limnology and Oceanography 14, 454–458.

EPA, 1996. Method 9034 - Titrimetric procedure for acid-soluble and acid insoluble sulfides.

Kubeneck, L.J., Notini, L., Rothwell, K.A., Fantappiè, G., Huthwelker, T., ThomasArrigo, L.K., Kretzschmar, R., 2024. Transformation of vivianite in intertidal sediments with contrasting sulfide conditions. Geochimica et Cosmochimica Acta 370, 173–187. https://doi.org/10.1016/j.gca.2024.01.020

Lagarec, K., Rancourt, D.G., 1998. Recoil - Mössbauer spectral analysis software for Windows. Ottawa, ON, Canada: Department of Physics, University of Ottawa version 1.0, 43.

Lagarec, K., Rancourt, D.G., 1997. Extended Voigt-based analytic lineshape method for determining N-dimensional correlated hyperfine parameter distributions in Mössbauer spectroscopy. Nuclear Instruments and Methods in Physics Research Section B: Beam Interactions with Materials and Atoms 129, 266–280. https://doi.org/10.1016/S0168-583X(97)00284-X

Lefebvre, P., Le Pape, P., Mangeret, A., Gourgiotis, A., Sabatier, P., Louvat, P., Diez, O., Mathon, O., Hunault, M.O.J.Y., Baya, C., Darricau, L., Cazala, C., Bargar, J.R., Gaillardet, J., Morin, G., 2022. Uranium sorption to organic matter and long-term accumulation in a pristine alpine wetland. Geochimica et Cosmochimica Acta 338, 322–346. https://doi.org/10.1016/j.gca.2022.10.018

Peiffer, S., Pecher, K., 1997. Experimentelle aquatische Chemie. Spektrum, Akad. Verl.

Ravel, B., Newville, M., 2005. ATHENA, ARTEMIS, HEPHAESTUS: data analysis for X-ray absorption spectroscopy using IFEFFIT. J Synchrotron Rad 12, 537–541. https://doi.org/10.1107/S0909049505012719

Schulz, K., Notini, L., C. Grigg, A.R., Joëlle Kubeneck, L., Wisawapipat, W., K. ThomasArrigo, L., Kretzschmar, R., 2023. Contact with soil impacts ferrihydrite and lepidocrocite transformations during redox cycling in a paddy soil. Environmental Science: Processes & Impacts 25, 1945–1961. https://doi.org/10.1039/D3EM00314K

Schulz, K., ThomasArrigo, L.K., Kaegi, R., Kretzschmar, R., 2022. Stabilization of Ferrihydrite and Lepidocrocite by Silicate during Fe(II)-Catalyzed Mineral Transformation: Impact on Particle Morphology and Silicate Distribution. Environ. Sci. Technol. 56, 5929–5938. https://doi.org/10.1021/acs.est.1c08789

Schwertmann, U., Cornell, R.M., 2000. Iron Oxides in the Laboratory: Preparation and Characterization. John Wiley & Sons.

Solé, V.A., Papillon, E., Cotte, M., Walter, Ph., Susini, J., 2007. A multiplatform code for the analysis of energy-dispersive X-ray fluorescence spectra. Spectrochimica Acta Part B: Atomic Spectroscopy 62, 63–68. https://doi.org/10.1016/j.sab.2006.12.002

Tamura, H., Goto, K., Yotsuyanagi, T., Nagayama, M., 1974. Spectrophotometric determination of iron(II) with 1,10-phenanthroline in the presence of large amounts of iron(III). Talanta 21, 314–318. https://doi.org/10.1016/0039-9140(74)80012-3
